# Human Sexual Cycles are Driven by Culture and Match Collective Moods

**DOI:** 10.1038/s41598-017-18262-5

**Published:** 2017-12-21

**Authors:** Ian B. Wood, Pedro L. Varela, Johan Bollen, Luis M. Rocha, Joana Gonçalves-Sá

**Affiliations:** 1 School of Informatics & Computing, Indiana University, Bloomington, IN USA; 2 Instituto Gulbenkian de Ciência, Oeiras, Portugal; 3 Wageningen University, Wageningen, The Netherlands

## Abstract

Human reproduction does not happen uniformly throughout the year and what drives human sexual cycles is a long-standing question. The literature is mixed with respect to whether biological or cultural factors best explain these cycles. The biological hypothesis proposes that human reproductive cycles are an adaptation to the seasonal (hemisphere-dependent) cycles, while the cultural hypothesis proposes that conception dates vary mostly due to cultural factors, such as holidays. However, for many countries, common records used to investigate these hypotheses are incomplete or unavailable, biasing existing analysis towards Northern Hemisphere Christian countries. Here we show that interest in sex peaks sharply online during major cultural and religious celebrations, regardless of hemisphere location. This online interest, when shifted by nine months, corresponds to documented human births, even after adjusting for numerous factors such as language and amount of free time due to holidays. We further show that mood, measured independently on Twitter, contains distinct collective emotions associated with those cultural celebrations. Our results provide converging evidence that the cyclic sexual and reproductive behavior of human populations is mostly driven by culture and that this interest in sex is associated with specific emotions, characteristic of major cultural and religious celebrations.

## Introduction

Human reproduction shows a yearly cyclical pattern and whether this periodicity is driven primarily by cultural or by biological factors has been an open question for several decades. In Western, Northern Hemisphere countries, births tend to peak in September, corresponding to early winter conceptions^1^. These conception dates are aligned with the December solstice which has been taken as evidence for the existence of an environment-induced biological clock that drives human reproduction cycles^2,3^. Proposed evolutionary explanations include temperature^4^, libido, or the availability of food^1,5^. However, this conception peak also coincides with religious celebrations, like Christmas, suggesting that culture drives the observed birth cycles. Culture and biology certainly influence each other, and it is very likely that both influence sexual drive. However, whether biological or cultural factors best explain the reproduction cycle has long been debated in the literature, with biological explanations dominating the argument^1^.

The biological hypothesis proposes that human reproductive cycles are an adaptation to the seasonal cycles caused by hemisphere positioning in the yearly orbit of the Earth around the Sun. If true, reproductive periodicity should be similar among Northern Hemisphere countries, less pronounced closer to the equator, and reversed in Southern Hemisphere countries^6^. On the other hand, the cultural hypothesis proposes that conception dates vary mostly due to cultural factors, such as holidays or seasonal marriage patterns^3^. If true, we should see similar sexual cycles in similar cultures independent of hemisphere. To study these hypotheses we need to measure sexual activity on a planetary scale. Common proxies for such measurements include birth records, incidence of sexually transmitted diseases, or condom sales^7^. However, for many countries these records are inaccurate with respect to the timing of sexual activity^8,9^ and a focus on hospital records (for births or sexually transmitted diseases) would largely restrict analysis to “Western” countries, where such data tends to be most commonly available. Thus, previous indicators do not offer sufficiently accurate data from across the globe to help distinguish between the two hypotheses.

The recent availability of large-scale population data from web searches and social media now allows us to study collective social behavior on a global scale. In this work, we gauge interest in sex directly from Google searches and characterize seasonal population sentiment from the analysis of Twitter feeds. We show that analysis of this large-scale online activity can be used as a proxy for real-life actions and help answer longstanding scientific questions about human behavior.

## Results

### Worldwide Variations in Sexual Interest

To measure interest in sex, for each country, we retrieved the frequency by which people searched for the word “sex” using Google Trends^tm^ (GT)^10^ (Methods 1–3); henceforth referred to as “sex-searches.” Interestingly, even in countries where English is not an official language, the English term “sex” is either more searched for than the corresponding word in the local languages or they are strongly correlated (Supplementary Table S1). Moreover, the terms most associated with searches for “sex” in GT refer to direct interest in sex and pornography (Supplementary Table S1). Therefore, GT searches for the term “sex” are a good proxy for interest in sexual behavior in the countries analyzed in this study.

**Figure 1:**
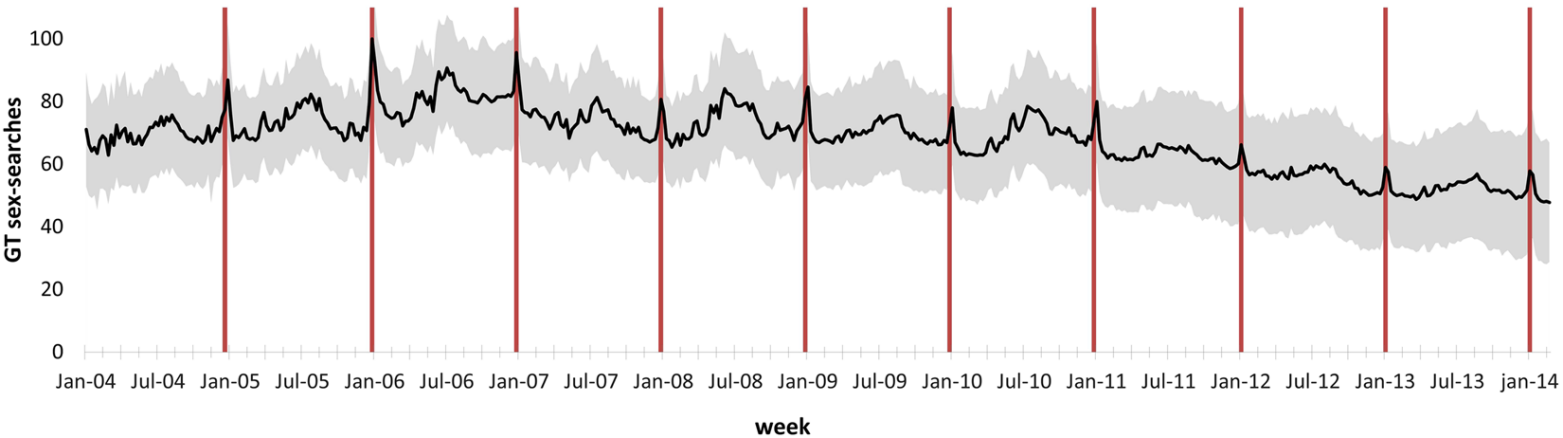
Weekly queries for the term “sex” for a group of representative western Northern countries. The black line represents the averaged queries in a 10-year period, obtained from Google Trends, which is normalized by overall search volume. These countries are: Austria, Canada, Denmark, Finland, France, Germany, Italy, Lithuania, Malta, Netherlands, Poland, Portugal, Spain, Sweden and the United States of America. Shaded grey represents the standard deviation. The red vertical line marks Christmas week.

Figure 1 depicts GT weekly sex-search data for 10 years from January 2004 to February 2014 for a set of Northern countries, which celebrate Christmas on December 25th. Yearly maximum peaks occur during Christmas week (red vertical lines), as previously observed for the USA^11^. While one may think that this increased interest in sex results simply from more free time during the holiday season, GT data is normalized by overall search volume^10^; even in a situation of increased general online activity, the increase in sexual interest is higher. Conversely, we could expect the holiday season to lead to a decrease in overall searches, led by school vacations for instance, originating an artificial peak for sex-related interest. However, we do not observe similar increases in weekly sex-searches for other widely observed holidays, such as Thanksgiving in the USA or Easter in France (Fig. S1A and B). Furthermore, a putative decrease in overall searches is unlikely, as a decrease in searches for school-related material can be compensated by a strong increase in searches for “presents” or “recipes”. In fact, when we control for search-volume of very common words, such as “on”, “and”, or “the”, there is some variation around the holiday period but it is in different directions for different search terms (Fig. S2A and B), probably resulting in an overall neutral change. Therefore, and although other dates lead to an increase in sex-searches (Fig. S1A and B), the Christmas holiday is uniquely associated with the highest peaks in sex-searches observed in these Northern countries. It is also known that, in Western Northern countries, conceptions peak around Christmas, in what some refer to as the “holiday effect”^12^. Indeed, the observed sex-search peaks match birth rate increases for this set of countries when shifted by nine months (Fig. S3A), which further confirms GT sex-searches as a good proxy for sexual activity.

Compared to the observation of sex-search peaks in Northern countries that celebrate Christmas on December 25th (and corresponding increase in September birth rates where such data is available), the two hypotheses outlined above would predict quite distinct observations for other cultures and hemisphere locations. If the biological hypothesis is correct, all Northern countries should have similar sex-search peaks around the same time, and these peaks should occur in a counter-phase pattern (six months later) in all Southern countries—irrespective of culture. On the other hand, if the cultural hypothesis is true, these peaks should appear anywhere Christmas is celebrated—irrespective of hemisphere—and other similar celebrations in different cultures should lead to sex-search peaks in other times of the year.

To test these predictions, we extracted GT sex-search time-series data for all 129 countries for which GT offered consistent data. Countries were categorized according to hemisphere (North or South) and their predominant religion^13,14^. Countries where at least 50% of the population self-identifies as Christian were considered culturally Christian countries, and similarly for Muslim countries. Other countries, where neither of these religions is dominant, were grouped separately; Supplementary Table S2 shows the complete list of countries and categorization.

Both Northern and Southern countries show a prominent peak in sex-searches around Christmas and we observe no counter-phase pattern corresponding to the southern hemisphere winter solstice of June 21st (see Figs S4A and S5C,D). In fact, there is a strong significant correlation (R2 = 0.54, p-value < 0.001) between the mean sex-search time series of Northern and Southern countries (Supplementary Table S3). Since most Northern and Southern countries for which we have data identify as Christian (80 of 129), the observed correlation suggests that a cultural effect, rather than hemisphere location, drives the Christmas sex-search peak. Indeed, the birth data available for Christian, Southern countries peaks with Christmas sex-searches when shifted by nine months in much the same way as for Christian, Northern Countries, even though it is summer in the former and winter in the latter (Fig. S3). Furthermore, there is neither a sex-searches increase in December nor a birth peak in September for Northern countries that do not celebrate Christmas on December 25th (Fig. S7). As reliable birth data is not generally available, particularly for Southern and Muslim countries, and is only available for four Southern countries, all of them predominantly Christian, (Methods 6, Supplementary Table S9 and Figs S3 and S6), we use GT sex-search data instead to observe many more countries and address the two hypotheses.

**Figure 2:**
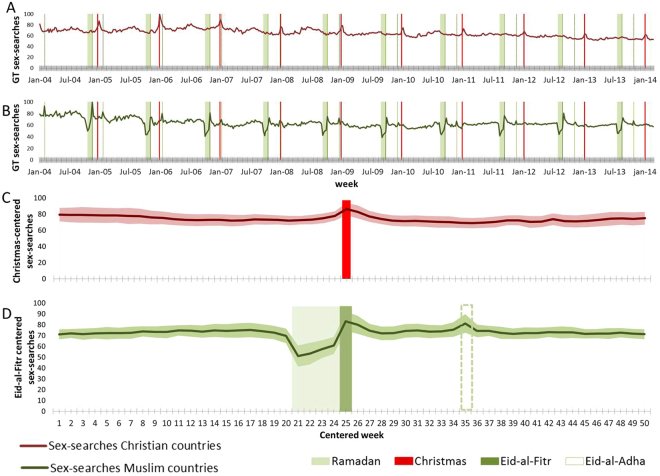
Weekly queries for the term “sex” in culturally different countries. (**A**) Normalized and averaged queries for all available countries identified as Christian (dark red line). (**B**) Normalized and averaged queries for all available countries identified as Muslim (dark green line). (**C**) Searches in all Christian countries centered around Christmas week^21^. (**D**) Searches in all Muslim countries centered around Eid-al-Fitr week^24^. See Supplementary Table 2 for country identification and availability on GT. The vertical red lines mark Christmas week, the shaded light green area represents Ramadan, with the darker green lines marking Eid-al-Fitr (solid) and Eid-al-Adha (dashed). Shaded areas around the lines in **C** and **D** show the standard deviation.

Parsing all countries by religion (Fig. 2A and B, Fig. S4 and Supplementary Table S3), it is clear that the mean sex-search time-series are periodic but uncorrelated between Christian and Muslim countries (R2 = 0.19, p-value < 0.001). The difference in sex-search behavior between these two sets of countries is further revealed in Fig. 2C and D, where we averaged the sex-search yearly time-series across all ten years centered on Christmas week (for Christian countries) or centered on Eid-al-Fitr, the major family holiday, that ends Ramadan (for Muslim countries). In Christian countries, the only clear peak occurs during the Christmas week. In contrast, in Muslim countries there is a peak during the week of Eid-al-Fitr and a second peak during the week of Eid-al-Adha, the other major religious and family celebration in Muslim culture; also noteworthy is a steep decrease during Ramadan, consistent with that period of general abstinence (as further discussed below). Both of these groups of countries clearly show sex-search peaks associated with distinct cultural celebrations, rather than with hemisphere. Indeed, it is worth noting that the Muslim calendar does not follow the solar calendar: every year Ramadan shifts by 10 days relative to its date during the previous Gregorian calendar year. Nevertheless, sex-searches peak during the moving week of Eid-al-Fitr (and Eid-al-Adha) in Muslim countries. The moving sex-search peaks associated with major religious events in Muslim countries further emphasizes the cultural driver behind such collective behavior.

**Figure 3:**
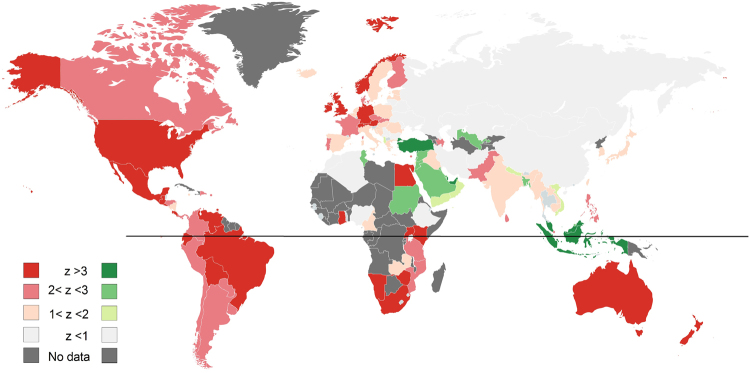
World-wide sex-search profiles. The world map is color-coded according to the z-score of each individual country’s sex-search time-series. Shades of red represent a higher z-score (larger increase in searches) during Christmas week (on Christmas-centered data). Shades of green represent a higher z-score (larger increase in searches) during Eid-al-Fitr week (on Eid-al-Fitr centered data). Light grey denotes countries with no significant variation above mean in either of these weeks. Dark grey countries are those for which there is no GT data available. Black line represents the equator separating the hemispheres. Built using: https://mapchart.net/.

To resolve the incompatible predictions of the biological and cultural hypotheses we made country-specific comparisons between hemisphere and culture, beyond the group-average behavior described above. We averaged the yearly sex-search time-series for each of the 129 individual countries across all years in four different ways: centered on Christmas week (fixed relative to the solar calendar), centered on Eid-al-Fitr week (moving relative to the solar calendar), and centered on each of the solstices, fixed on June 21st and December 21st (Methods 4, Supplementary Tables S4–6 and Fig. S5). We then measured the response of countries to a holiday as the sex-search z-score deviation above the mean at Christmas, Eid-al-Fitr and the two solstice weeks (Methods 5 and Supplementary Table S7). Figure 3 shows a world map with color-coded countries: shades of red indicate countries whose highest sex-search deviation from mean occurs during the Christmas week, and shades of green indicate countries whose highest sex-search deviation from mean occurs during Eid-al-Fitr week (Methods 7). It is clear that this response yields a map organized according to culture rather than hemisphere.

We then compared this new country classification (according to the individual countries’ sex-search profile, Supplementary Table S7 and Supplementary Methods S1) with our previous identification based only on the proportion of the population that self-identified as Christian, Muslim or Other (Supplementary Table S2)^13,14^. Out of the 30 countries originally identified as Muslim^14^, 77% show a significant increase (z > 1) in sex-searches during the week of Eid-al-Fitr, and out of the 80 countries originally identified as Christian^13^, 80% show a significant increase (z > 1) during the Christmas week, regardless of the hemisphere. It is important to note that this correspondence is even higher (91%) when we identify as “Other” the ten Christian countries that do not celebrate Christmas on December 25th. In fact, we do not see an increase in sex searches around December 25th in any of these Northern Russian and Serbian Orthodox Christian countries, which celebrate Christmas in early January, and this further supports the cultural hypothesis (Methods 2, Supplementary Methods S1, Supplementary Figure S7). Moreover, only 14% of Southern countries showed a significant increase in sex-searches during the June solstice (Supplementary Tables S7 and S8B), demonstrating that there is no significant counter-phase sex-search peak in the southern hemisphere, contradicting the biological hypothesis.

### Trends in Holiday Moods

The Christmas and Eid-al-Fitr holidays carry significant cultural and religious meaning, but they are not directly associated with sex. It is, in fact, very counter-intuitive to think of Christmas and Eid as the times of the year with the most online searches for sex. However, these events may trigger specific and collective moods, leading to a striking correspondence between these holidays and sexual interest. To investigate the emotional factors involved we measured changes in public sentiment on Twitter^15–17^. The analysis was performed before, during, and after Christmas and Eid-al-Fitr in a set of seven countries with sufficient Twitter traffic in our data: Australia, Argentina, Brazil, Chile, Indonesia, Turkey, and the USA (Methods 9 and Fig. S8). Although it is not possible to know whether the Google and Twitter populations are the same per country, given the large volume of Google searches and tweets, it is very likely that they provide a significant sample of the same populations.

Twitter sentiment was quantified by rating a random 10% sample of all tweets posted between September 2010 to February 2014 using the Affective Norms for English Words (ANEW) lexicon^18^ (Methods 8 and 9). The ANEW lexicon consists of 1,034 English words that carry a sentiment score along three dimensions: Arousal (a), Dominance (d), and Valence (v), corresponding respectively to whether the word makes human raters feel calm vs. excited, controlled vs. in-control, and sad vs. happy. The sentiment value of a single tweet is defined as the mean ANEW score of its words. We translated the lexicon to Spanish and Portuguese to capture public sentiment in those languages as well, but did not have the ability to translate into additional languages. To avoid bias from holiday-related language, we ignored all words used in traditional greetings for all known holidays in the World (Supplementary Table S13); we also removed the word “Christmas” and “valentine” from the lexicon, which does not include other holiday names.

We first observed that the weekly volume of sex-searches significantly correlates with the mean weekly sentiment derived from the three ANEW dimensions in a multiple linear regression (Supplementary Methods S2, Supplementary Table S10). In every country, valence yields a positive coefficient, while dominance a negative coefficient; thus the happier but less in-control the population mood is, the more sex-searches tend to increase in every country (Methods 10 and Supplementary Methods S2). Interestingly, while public sentiment displays a strong linear relationship with sex-search volume when all mood dimensions are considered, there is little correlation with each ANEW dimension on its own (Supplementary Table S11).However, the observed linear correlation does not allow us to characterize the population mood in the target cultural celebrations. To investigate if days that are similar in mood to Christmas in Christian Countries or to Eid-al-Fitr in Muslim Countries also tend to observe increased volume of sex-searches, we need a more nuanced characterization of the mood profile each week.

Because collective mood sentiment, as measured here, is derived from many tweets of large and diverse populations, it can contain distinct and informative components. Thus, we employed an eigenvector-based analysis^19^ to characterize the distribution of sentiment values, rather than just average sentiment. We thus obtain the components of public sentiment that explain most of the variance in the data not attributable to regular language use, hereafter referred to as “eigenmoods.” Specifically, an eigenmood is a small set of components (eigenvectors) of a matrix. In this matrix, the rows denote sentiment scores in a given range or bin, and the columns denote the weeks (Methods 11 and Supplementary Methods S3), and elements are the number of tweets during a week that fall in that bin. Thus, an eigenmood is not an average sentiment value (per week in our analysis), but rather a change in the distribution of sentiment that explains a significant proportion of the variation in the time-series data^20^.

**Figure 4:**
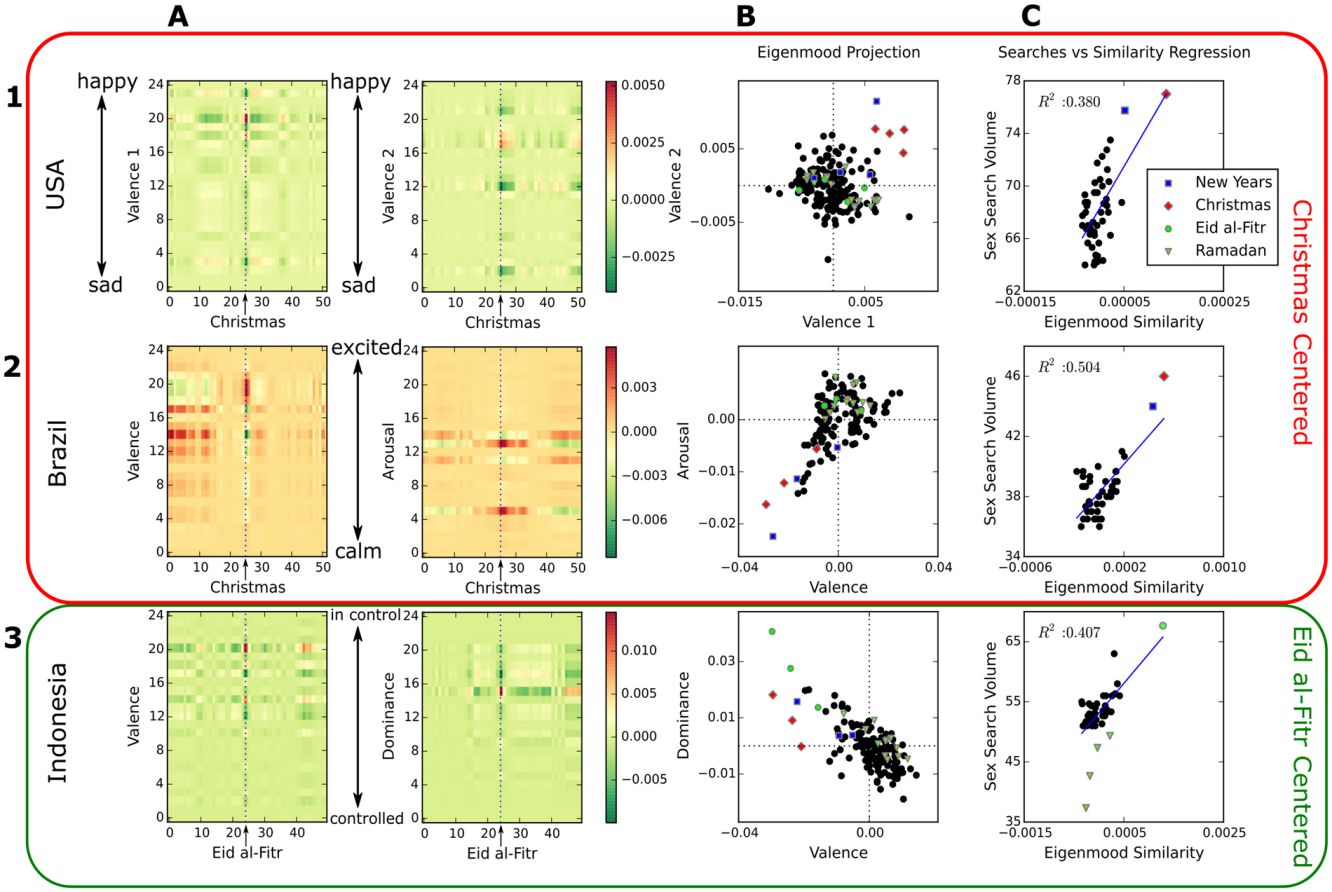
Mood distributions and their correlations with sex-searches. Rows: 1 - USA centered on Christmas, **2** - Brazil centered on Christmas, **3** - Indonesia centered on Eid al-Fitr. Columns: (**A**) Heatmaps of sentiment distribution reconstructed from selected eigenmoods. Vertical axis specifies the bins of the ANEW distribution for a given mood dimension, from low (bottom) to high (top) values. Eigenmood components were selected to best characterize the respective holiday and country (after removing the first component). In the case of the USA (Row 1), the two selected components both fall in the “valence” dimension and are labelled valence1 and valence2; for Brazil (Row 2) and Indonesia (Row 3) the first component also falls in the “valence” mood dimension, but the second falls in the “arousal” and “dominance” dimensions, respectively. Horizontal axis specifies the week of the centered, averaged year (52 weeks for the Gregorian calendar, 50 for the Muslim Calendar). The dotted line in the center marks the holiday of interest, on week 26 for Christmas, or week 25 for Eid al-Fitr. Color represents the weight of the eigenmood per bin per week. (**B**) Projections of weeks into the space formed by the selected eigenmood components. Each axis specifies the projection of week onto each component that defines the eigenmood. See text for details and supplemental materials for more information on component selection. (**C)** Linear regressions between GT sex search volume (vertical-axis) and similarity to holiday center in the Twitter eigenmood space depicted in column B (horizontal-axis) for averaged weeks. The weeks of Ramadan are shown with increasing color intensity from more yellow to more green as they approach Eid-al-Fitr. The R^2^ values for the regressions are 0.380 for Christmas in the USA, 0.504 for Christmas in Brazil, and 0.407 (0.637 without the Ramadan weeks) for Eid-al-Fitr in Indonesia.

We found that two components were sufficient to describe public sentiment associated with each holiday and country – a characterization that is independent of sex-search volume, and relies only on measurement of sentiment on Twitter (Methods 10–12, Supplementary Methods S3–5, and Supplementary Figs S10 and S11). Figure 4 (Column A), Figs S9 and S14 show the sentiment distribution of the selected eigenmoods that best characterize the holidays of interest, per every week of the year; redder (greener) colors represent increased (decreased) numbers of tweets falling in the respective mood dimension bins – e.g., for valence, upper bins on vertical axis denote increased happiness and lower bins denote increased sadness. The sentiment distributions of rows 1, 2, and 3 in Fig. 4 column A are centered on Christmas for USA (Northern, Christian) and Brazil (Southern, Christian), and Eid-al-Fitr for Indonesia (Southern, Muslim). While the eigenmood that describes Christmas in the USA uses only the valence dimension of ANEW, the best eigenmood for Christmas in Brazil requires valence and arousal, and for Eid-al-Fitr in Indonesia requires valence and dominance. The sentiment distribution of these eigenmoods per week clearly shows that significant and unique changes in sentiment occur during the target holidays. In all these cases, the public mood of the holiday in question generally shifts to “happy” bins (more red in higher valence) and away from “sad” bins (more green in lower valence). In Brazil, the mood also shifts to more “calm” bins during Christmas week (more red in lower arousal), and in Indonesia it also shifts to neither “in-control” nor “controlled” bins during the Eid al-Fitr week (more red in mid dominance). More detailed characterization of eigenmoods and their selection for each country is provided in Supplementary Material (Supplementary Methods 3–5 Fig. S12 and 13).

Figure 4, column B, shows all weeks in the data projected onto the selected eigenmood space of two components for each country. It is clear that in this space Christmas weeks (red diamonds) cluster together for the USA and Brazil, and Eid-al-Fitr weeks (green circles) cluster together for Indonesia, demonstrating that the eigenmoods are consistent in different years for each holiday in each country. Figure 4 column C depicts the linear regression between sex-search volume as calculated before (vertical axis), and mood similarity to the target holiday in the respective eigenmood space (horizontal axis) for all weeks in the data set denoted by black circles in the plot (Methods 14 and Supplementary Methods S6). We observe a significant correlation for all countries studied, with R2 ≥ 0.38 for Christmas in all Christian Countries and R2 ≥ 0.34 for Eid-al-Fitr in all Muslim Countries, irrespective of hemisphere (Supplementary Table S12). Thus, in Christian countries we can say that the more the public mood of any week resembles the Christmas eigenmood, the larger the volume of observed sex-searches tends to be. Similarly, in Muslim Countries the more public mood is similar to the Eid-Al-Fitr eigenmood, the larger is the volume of sex-searches. In the case of both Muslim Countries studied (Indonesia and Turkey), there is a striking result pertaining to Ramadan: those 4 weeks (4 lowest green triangles in Fig. 4C, bottom right, for Indonesia), have the lowest sex-search volume by far in the data, consistent with the period of abstinence that marks Ramadan (see also Fig. 2B,D). The public mood during these weeks of Ramadan is also quite distinct from the Eid-al-Fitr mood (horizontal axis in Fig. 4C, bottom right), but, becomes more similar the closer the week is to Eid-al-Fitr in time; and as the mood becomes closer to the Eid-al-Fitr mood as Ramadan approaches its end, the sex-search volume also increases. Naturally, due to the low, outlier sex-search volume during Ramadan weeks, the linear regression is much stronger if those weeks are removed, with R2 ≥ 0.64 (Supplementary Table S12).

Thus, not only there are specific moods associated with Christmas and Eid-al-Fitr, the eigenmoods that best characterize these holidays significantly correlate with increased interest in sex throughout the calendar. This is true in all countries studied, in both hemispheres and cultures. Moreover, and although these moods occur at different times in different cultures, they seem to be similar in essence and, in general, the “happier” the mood, the more it associates with sex interest.

## Discussion

Taken together, our analyses provide strong converging evidence for the cultural hypothesis: human reproductive cycles are driven by culture rather than biological adaptation to seasonal cycles. Furthermore, the observed peaks of interest in sex occur around family-oriented religious holidays, across different hemispheres and cultures, and the measured collective mood on these holidays correlates with interest in sex throughout the year, beyond these holidays. This correlation suggests that the cultural driver of reproductive cycles depends on the collective mood of human societies, though establishing such causality warrants further study. It is also worth noticing that while other major holidays in each country lead to increased sex-search volume (e.g. Eid-al-Adha), not all holidays exhibit this effect (e.g. Easter and Thanksgiving), suggesting that certain holidays have unique eigenmoods which lead to increased interest in sex at the population level. Thus, specific mood states−typically happier, calmer, and neither in-control nor controlled−are associated with interest in sex, and this collective emotion is universal and maximized during cultural celebrations such as Christmas and Eid-al-Fitr. The fact that the Muslim holidays do not follow a solar calendar, with the interest in sex varying according to the religious calendar, provides additional support for the cultural hypothesis.

It is clear from this work that culture (particularly the religious calendar) best explains the pattern of sexual interest. Naturally, it is important to stress that if collective mood states drive interest in sex at the individual level, there must ultimately be a common biological response to the cultural, contextual driver. Several hypotheses can be entertained − though not adaptation to seasonal cycles. For instance, some studies show that depressed people lose interest in sex and that “happy moods,” such as those uncovered for Christmas and Eid-al-Fitr, are usually more conducive to sexual arousal^21,22^. Increased food consumption has also been shown to have a relationship with sexual maturation and interest^23,24^, however, we do not see similar increase in sex-searches during other holidays associated with high food intake, such as Thanksgiving in the USA or Easter in France. And given the children and family focus of both Christmas and Eid-al-Fitr, it is reasonable to consider psychological and symbolic triggers to the observed behavior, but the neurological and biochemical pathways involved in such responses are as yet unknown.

That the culturally motivated surge in sexual interest can be so easily anticipated and measured has implications for public health and policy. Hospitals should be prepared for an increase in STD testing and possibly even abortions in the weeks following such holidays and when the corresponding collective mood is observed at other times of the year.

Overall, this work emphasizes the need for more world-scale studies and the importance of a better understanding of global collective behaviors at the level of individual countries. These will enable better-informed decisions and the more effective fine-tuning of policy towards the distinct needs of countries, cultures, and communities.

## Methods

### Google Trends Data

Google Trends (GT) provides a time series index of the search volume of a given Google query^10^. GT allows for searches in a selected region (country, state, city, etc.) and for a selected time range starting in January 2004 for most countries. Google normalizes the resulting query index relative to the total amount of query volume for a search term in the chosen area, per week, so that the maximum query share of the time series is set to be 100. GT queries are also broad matched, meaning that queries such as “sex videos” are counted in the calculation of the query index for “sex”.

### Country Selection and Categorization

We considered all countries for which GT is available and for which a search for “sex” had a least two contributing cities and had enough time points to analyze at least four consecutive holiday seasons (Christmas and Ramadan), thus starting at least in the last week of 2009. This was the case for 129 countries in all continents. In the paper these countries are identified either by their name or by the country code, as in Supplementary Table S2.

Countries were categorized according to their major religion and geographical location (continent and Northern or Southern Hemisphere according to Wikipedia) and this categorization is referred to “identification” in the main manuscript. A country was considered “culturally Christian” when at least half of its population identified as Christian (Catholic, Protestant, Orthodox, or other)^13^. A country was considered “culturally Muslim” when at least half of its population identified as Muslim^14^. A country was labeled as “Other” when the majority of its population didn’t identify as either Christian or Muslim. In the case of countries that have parts of their territory in both hemispheres, we used the location of the capital as the deciding criteria. Out of the countries identified as Christian, eleven have a majority that follow either the Russian or Serbian Orthodox Churches (namely: Belarus, Bosnia and Herzegovina, Bulgaria, Georgia, Macedonia, Moldova, Montenegro, Serbia, Slovenia, Russia and Ukraine). In ten of these countries (Bulgaria being the exception), Christmas is celebrated in early January (of the Gregorian Calendar) and they could have been labeled as Other for the proposes of this analysis.

### Searches for “sex”

We downloaded the time-series corresponding to searches for”sex” for each of the available countries in GT as long as they had at least two cities contributing data, and had enough time points to analyze at least four consecutive holiday seasons (Christmas and Ramadan), thus starting at least in the last week of 2009. Supplementary Table S2 shows all countries included in the analysis. Because Google does not provide the absolute number of searches and we do not have access to the normalization algorithm, all the analyzed data is relative to the total search volume and it has been noticed by ourselves and by others that there is some variation the output GT provide, from week to week. To limit this variation all of the analyzed data was downloaded on the same week.

For a subset of 50 countries (on all continents) we downloaded GT data for 2 search queries^1^: for the term “sex” and^2^ for its translation in the local language. We compared the volume of searches between the two queries and calculated their correlation over time. Supplementary Table S1 shows the 25 countries and languages that retrieved a sufficiently significant search volume in the local language to support our analysis. We then calculated the “Search Volume Ratio”, as the number of searches for “sex” divided by the number of searches for the corresponding translation. We also calculated the Correlation between the two time series (“sex” and the translated word) as the Pearson´s R.

GT also provides and ranks the top words associated with the search term and these are also shown on Supplementary Table S1.

### Centered Calendars

Data were organized into yearly “calendars” centered around the holidays of interest in order to better compare time series across cultures, and to create better summaries of averaged yearly time-series. Five “yearly calendars”, or sets, were constructed:

1. The first, a “Civil Calendar” starts on the first week that includes January 1st and ends on the following December 31st.
2. The second was centered around the weeks that contain Christmas. In this paper we refer to it as the “Christian Calendar”.
3. The third was centered around the weeks that contain the Eid-al-Fitr celebrations. In this paper we refer to it as the “Muslim Calendar”.
4. The fourth was centered around June 21st and is referred to as the “June Solstice Calendar”;
5. The fifth was centered around December 21st and is referred to as the “December Solstice Calendar”.

Each week of each calendar was given an index ranging from 1 to the maximum number of weeks in that year. The first week GT indexes starts at the Jan 1 2004, so all remaining weeks will start seven days from this first index. In our centered calendars, the week containing Christmas and the solstices becomes week 26 and the week containing Eid-al-Fitr becomes week 25. This is because both the “Civil”, “Solstices” and”Christmas” calendars follow the Gregorian Calendar with 52.177457 weeks per year, but the”Muslim Calendar” follows a lunar calendar with 29.53 days per month, leading to 354 or 355 days per year. Since the “Muslim Calendar” is consistently shorter than the solar year, it shifts with respect to the Gregorian calendar, necessitating the removal of these extra weeks as they contained no major event or holiday. Thus, Christmas was specified as week 26 in a 52 week calendar (starting from week 1), and Eid-al-Fitr as week 25 in a 50 week calendar. Occasional exception weeks were dropped from analysis if they did not fit into these calendars, without greatly altering the analysis; see Supplementary Tables 4–6 for the complete list. Supplementary Figure S5 shows the plot of all countries, centered around the weeks that contain Christmas, Eid-al-Fitr or January 1st, averaged according to their cultural identification (see above).

### Country Classification from sex-searches

If sex searches correspond to countries’ self-reported religions or locations (as described in the Country Selection and Categorization section), we can use sex searches as a feature to classify countries. Here we describe the process by which sex searches were used to measure a country’s response to events: Eid al-Fitr, Christmas, the December Solstice, and the June Solstice. These responses were used to evaluate sex searches as a feature in a classification task. The centered time series described before were calculated for all countries in Supplementary Table S2. For each country we obtained between 4 and 9 yearly time series for all years for which data is available. These yearly time-series were averaged in five different ways per country: one following the civil Gregorian calendar, one centered on Christmas week, one centered on Eid- al-Fitr week, one centered on June 21st, representing the June solstice, and the last centered on December 21st, representing the December solstice. Average yearly time-series were created by first normalizing the data by year, such that the highest valued week each year was given a value of 1, and other weeks were expressed as a proportion of that maximum, in order to correct for bias towards years with more searches. To identify weeks with peak sex-search behavior, z-scores for each of these averaged time series were calculated as

$${\rm{z}}=({\rm{x}}-{\rm{\mu }})/{\rm{\sigma }}$$

where µ is the mean and σ is the (population) standard deviation.

We then pursued a simple classification of countries according to their behavior on the Christmas and Eid-al-Fitr weeks. When the averaged Christmas-centered (Eid-al-Fitr-centered) time-series for a country yields z > 1 on the Christmas (Eid-al-Fitr) week, the country was classified as a Christian Country (Muslim Country). If z < 1 for both the Christmas- and Eid-al-Fitr-centered time-series, then such a country is classified as Other. If z > 1 for both Christmas- and Eid-al-Fitr-centered time-series, the country was culturally associated with largest z. Results can be seen in Supplementary Table S7. A similar procedure was followed to compare countries according to geographical location. See also Supplementary Methods S1.

### Birth Data

There are biases and problems with birth data. This data is particularly uncommon in Muslim and Southern countries and is further confused in Muslim countries both by the fact that religious events do not follow the solar calendar and that registration dates do not accurately match actual birth dates (see Supplementary Materials Fig. 6). Nevertheless, if online sex-searches correspond to an actual increase in sexual activity, it should be possible to see an increase in births for countries where good records exist.

Monthly birth rates were collected from the United Nations Database^25^ (except for South Africa, retrieved from http://www.statssa.gov.za/publications/P0305/P03052012.pdf), See Supplementary Table S9 for data.

For each country, each month was divided by the number of days in the month (February months were divided by 28.25), then each year was normalized to its maximum value. This removes any bias towards years with more births.

To compare monthly birth rates with GT results we were restricted by the time range constraints of both data sets. We only have GT results from 2004 onwards and we rarely have birth data beyond 2012. Supplementary Table S9 shows the availability of birth data for all countries used in this study.

There is also no increase in sex-searches or September births in Northern countries that do not celebrate Christmas on December 25th (Supplementary Figures S7). In addition, there is independent evidence that, even within the same country, religiously distinct populations−such as the Muslim and Jewish populations of Israel−have different conception patterns that correlate with their religious holidays^26^.

### World Map

Countries were color coded according to the z-scores presented in Supplementary Table S7. The World Map was built using the online tool: https://mapchart.net/.

### ANEW

The sentiment in tweets was quantified according to the Affective Norms for English Words (ANEW) lexicon^18,27^. The ANEW assigns a number between 1 and 9 along three dimensions to 1034 words. These dimensions are arousal (a), dominance (d), and valence (v). The scores were determined through a survey as the mean score participants assigned each word. The valence scores correspond to whether (from 1 to 9) the word made participants feel sad to happy, arousal from calm to excited, and dominance from controlled to in-control For example, the word “laughter” has a valence score of 8.5, while “leprosy” has a score of 2.1. A basic translation to Spanish and Portuguese was performed through Google Translate and refined by speakers.

### Twitter Data

The source of the twitter data used comes from IU’s twitter garden hose feed, a 10% sample of all tweets. Geo-location data in combination with shape objects^28^ allowed the country from which a tweet came to be determined for many tweets. We focus on tweets collected between September 2010, when the collection stabilized, and February 2014, when the tweet collection dropped, complicating homogeneous analysis of the data. We analyzed seven countries that yielded a sufficiently large number of tweets per week (about ten thousand): Argentina, Australia, Brazil, Chile, Indonesia, Turkey, and the USA. This includes countries in both hemispheres, both culturally Christian and Muslim, and with both English and Other official languages. Individual country’s tweets were only examined after their collection had stabilized, starting in September 2010 for the US, Australia, and Chile; May 2011 for Indonesia and Brazil; June 2011 for Argentina, and September 2011 for Turkey. Days were defined according to Greenwich Mean Time, and weeks from Sunday to midnight Saturday. The overall number of weekly collected tweets are shown in Supplementary Fig. S8, ranging from nearly a million scored tweets per week from the USA and Brazil, to only about ten thousand scored tweets from Turkey and Australia. The proportion of scored tweets to all collected tweets is usually quite small, usually below 5%.

An individual tweet’s sentiment score was determined by finding all words within the tweet that matched the ANEW lexicon, and taking the average of their scores in each dimension. In the case that multiple languages were matched, the scores from the language with the most matched words were used. In case of a tie, the average scores over the tying languages were calculated. To better find the actual sentiment during the holidays without generic seasonal greetings, we don’t score words if they appear in generic holiday greetings, such as “happy holidays”, and we remove the ANEW words Christmas and Valentine from the lexicon entirely. The list of holidays whose greetings we removed were collected from http://www.officeholidays.com/. The complete list of phrases we removed from score calculation is included in Supplementary Table S13.

### Mean Sentiment Correlations with Sex-Search Volume

To see if sentiment in tweets correlates with sex search volume we computed the ordinary least squares estimate of a multiple linear regression for each country, using the time series of mean tweet sentiment each week along the three ANEW dimensions as independent variables, with the weekly volume of sex searches as the dependent variable. To compute the weekly mean sentiment time series for ANEW dimension, we first calculated the mean tweet sentiment score for each day and then calculated the mean sentiment of the week such that each day has an equal weight in the weekly average.

### Singular Value Decomposition for Eigenmood Analysis

Aggregating all sentiment in tweets into a mean value discards information in the distribution of sentiment across tweets. Therefore, we use binned distributions of sentiment across tweets in the following analysis. We focus on a 25-binned distribution of tweet sentiment between the lowest and highest possible ANEW score as a moderately-grained distribution, with fine enough resolution to capture some detailed structure while aggregating an adequate number of tweets per bin, 400 on average for a collection of 10^4^ tweets.

We applied a singular value decomposition (SVD)^19^ to the binned distribution of ANEW scores over time. Our matrix M has columns representing bins, and rows representing weeks. The left and right singular vectors then have an interpretation as the “eigenbins” and “eigenweeks” respectively. We will also refer to the singular vectors as components. The first component explains the vast majority of the variance, and is similar to the base distribution of the language, as expected from the Brown corpus, shown in^20^. The second component explains a trend over time, while further components correspond to other fluctuations, including yearly variations for holidays. For more information see also Supplementary Methods S3.

### Data Reconstruction

To analyze how sentiment varies, rather than its basic distribution in language use, we reconstructed the original data without the first component. After recalculating the relative variances, we can remove noise by also removing the components explaining the least variance. Reconstruction then includes only those components that explain 95% of the remaining variance after the first component is removed. This leaves cyclic patterns and outlier weeks deviating strongly from the baseline sentiment distribution, which we visualize as a heatmap of the distribution over time. We average over all full years in the data for multiple countries, centered on the week of a strong cultural holiday, to emphasize the change in these distributions, as shown in Supplementary Fig. 14. For more information see also Supplementary Methods S4.

### Eigenmood Selection

To investigate the distribution of sentiment in a country during a holiday, we selected an *eigenmood* composed of the two components that best characterized the mood distribution on the holiday. Supplementary Figure S15 and Supplementary Methods S5^29^. These two components were selected to describe a country’s twitter sentiment on a holiday in the following way. First, the average projection of the holiday was found over all years of the data, as well as the standard deviation. The two eigenweeks with the highest absolute value of the holiday’s projection minus its standard deviation were selected. The standard deviation is calculated over very few points, but subtracting it from the mean allows us to know how small the magnitude of the projected vector we may expect. This way, the mood of the holiday of interest can be expected to have a strong correlation with the selected components and cluster closely together.

### Eigenmood correlations to Sex-search volume in target Holidays

As a measure of mood similarity between weeks in a space defined by a selected eigenmood, we use the dot product between their coordinates in this space^19^. See Supplementary Methods M6 for more information^30^.

### Data Availability

Twitter data is subject to contractual limits and the original content of the tweets cannot be shared. However, all quantitative details that support the analysis are included in the Supplementary Materials, as is the dataset created from the Google Trends searches^31^.

## Electronic supplementary material

Supplementary Materials

Dataset 1
