# Author Correction: Human Sexual Cycles are Driven by Culture and Match Collective Moods

**DOI:** 10.1038/s41598-018-22522-3

**Published:** 2018-03-02

**Authors:** Ian B. Wood, Pedro L. Varela, Johan Bollen, Luis M. Rocha, Joana Gonçalves-Sá

**Affiliations:** 1 School of Informatics & Computing, Indiana University, Bloomington, IN USA; 2 Instituto Gulbenkian de Ciência, Oeiras, Portugal; 3 Wageningen University, Wageningen, The Netherlands

Correction to: *Scientific Reports* 10.1038/s41598-017-18262-5, published online 21 December 2017

In Figure 3, one of the countries is incorrectly coloured. The correct Figure 3 appears below as Figure 1.

**Figure 1:**
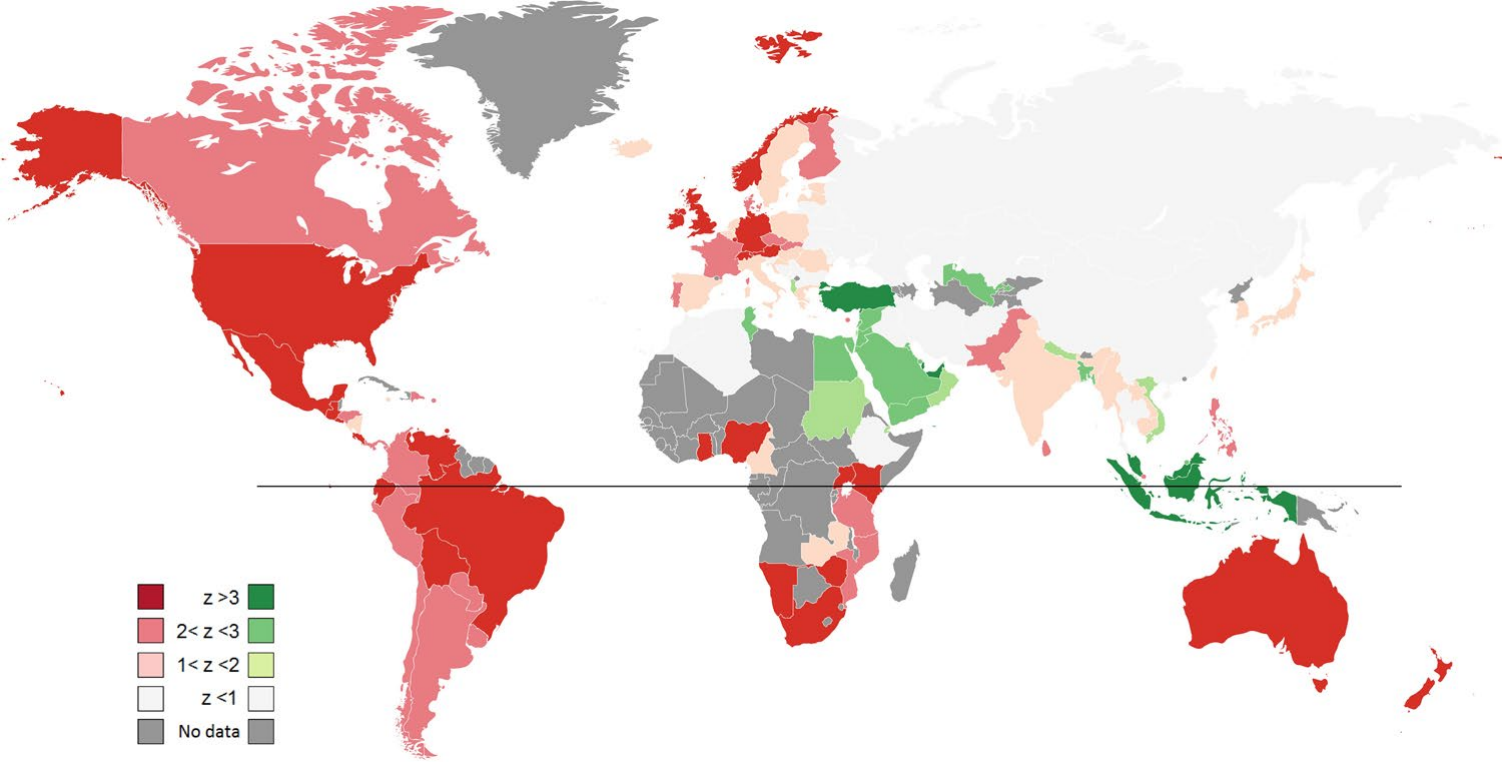
World-wide sex-search profiles. The world map is color-coded according to the z-score of each individual country’s sex-search time-series. Shades of red represent a higher z-score (larger increase in searches) during Christmas week (on Christmas-centered data). Shades of green represent a higher z-score (larger increase in searches) during Eid-al-Fitr week (on Eid-al-Fitr centered data). Light grey denotes countries with no significant variation above mean in either of these weeks. Dark grey countries are those for which there is no GT data available. Black line represents the equator separating the hemispheres. Built using: https://mapchart.net/.

The Acknowledgements section in this Article is incomplete.

“For this work, LMR and IBW were partially funded by the National Institutes of Health, National Library of Medicine Program, grant 01LM011945-01. JGS was partially supported by grant PTDC IVC ESCT 5337 2012, from the Fundação para a Ciência e a Tecnologia (FCT), and by the Welcome DFRH WIIA 60 2011, co-funded by the FCT and the Marie Curie Actions. JB was partially supported by grants from the Defense Advanced Research Projects Agency (DARPA) - NGS2 program (#D17AC00005) and the Economic Development Agency (EDA) ED17HDQ3120040. Twitter data collection was supported by NSF Award No. IIS-0811994. Google Trends10 and WorldBank data31 are publicly available. The authors also thank D. Rocha of Proposal Development Services at Indiana University for scientific editing and D. Junk for his work on data processing and collection. R. Correira, A. Gates, A. Kolchinsky, and other members of the CASCI group and CNetS at Indiana University and P. Almeida, M.M. Pita and other members of the S&P group at Instituto Gulbenkian de Ciência for their comments and assistance with this work.”

should read:

“For this work, LMR and IBW were partially funded by the National Institutes of Health, National Library of Medicine Program, grant 01LM011945-01. JGS was partially supported by grant PTDC IVC ESCT 5337 2012, from the Fundação para a Ciência e a Tecnologia (FCT), and by the Welcome DFRH WIIA 60 2011, co-funded by the FCT and the Marie Curie Actions. JB was partially supported by grants from the Defense Advanced Research Projects Agency (DARPA) - NGS2 program (#D17AC00005) and the Economic Development Agency (EDA) ED17HDQ3120040. Twitter data collection was supported by NSF Award No. IIS-0811994. Google Trends10 and WorldBank data31 are publicly available. The authors also thank D. Rocha of Proposal Development Services at Indiana University for scientific editing and D. Junk for his work on data processing and collection. R. Correira, A. Gates, A. Kolchinsky, and other members of the CASCI group and CNetS at Indiana University and P. Almeida, M.M. Pita and other members of the S&P group at Instituto Gulbenkian de Ciência for their comments and assistance with this work. The collaboration between JGS and LMR was partially supported by a Fulbright Commission fellowship to LMR.”

