# Supplementary material for: Human Sexual Cycles are Driven by Culture and Match Collective Moods
Source: Sci Rep. 2017 Dec 21;7:17973. doi: 10.1038/s41598-017-18262-5 (PMC5740080; doi:10.1038/s41598-017-18262-5)
Supplement: Supplementary file 1 — Supplementary Materials [file 41598_2017_18262_MOESM1_ESM.pdf]

Title: Human Sexual Cycles are Driven by Culture and Match Collective Moods

Authors: Ian B. Wood[1], Pedro Leal Varela[2], Johan Bollen[1,3], Luis M. Rocha[1,2]*, and Joana Gonçalves-Sá†[2]

Affiliations:

[1] School of Informatics & Computing, Indiana University, Bloomington, IN, USA.

[2] Instituto Gulbenkian de Ciência, Oeiras, Portugal.

[3]Wageningen University, The Netherlands.

Correspondence to: † mjsa@igc.gulbenkian.pt and *rocha@indiana.edu

Supplementary Materials

Supplementary Methods

S1. Notes on "misclassifications" for Country Classification from sex-searches
S2. Mean Sentiment Correlations with Sex-Search Volume
S3. Singular Value Decomposition
S4. Data Reconstruction
S5. Eigenmood Selection and Characterization
S6. Eigenmood correlations to Sex-search volume in target Holidays

Supplementary Figures

Fig. S1. GT query [sex] results for the USA.
Fig. S2. GT query [sex] results for France.
Fig. S3. Monthly birth data shifted by nine months and weekly averaged Google Trends results for "sex-searches"
Fig. S4. Averaged sex-searches
Fig. S5. Averaged holiday-centered results
Fig. S6. Monthly birth data for Turkey and Egypt
Fig. S7. Monthly birth data for Russian and Serbian Orthodox Countries, and South Korea
Fig. S8. Total number of weekly geolocated tweets matching ANEW for countries selected for Eigenmood analysis.
Fig. S9. Reconstructed valence heatmaps for multiple countries, centered on cultural holidays.
Fig. S10. ANEW component response to Christmas by country.
Fig. S11. ANEW component response to Eid-al-Fitr by country.
Fig. S12. Linguistic Variable value membership functions over 25 bins.
Fig. S13. Linguistic Variable Response to relevant holidays selected for each country.
Fig. S14. Average year reconstructed heatmaps.
Fig. S15. Eigenmood projections and regressions.

Supplementary Tables

Table S1. Searches for "sex" in select countries.
Table S2. Countries analyzed and categorized according to religion and geographical location (hemisphere).
Table S3. Correlation Table for the averaged time series of all countries grouped either by hemisphere (Northern or Southern) or by religion (Muslim or Christian).
Table S4. The three major Muslim holidays, in regard to the Gregorian calendar, for the period under analysis.
Table S5. Starting day of the "Christian Calendar", starting day of the weeks that included December 25th – Christmas (always on week 26), the last week of each centered year and the discarded exception weeks after centering.
Table S6. Weeks that included Eid-al-Fitr and the discarded exception weeks after centering.
Table S7. Z-scores on the corresponding centered week for all countries in the dataset, calculated from the each country's average for each week, as detailed in the Methods.
Table S8. Correlation between the Z-scores' time series for all countries in the data set.
Table S9. Monthly birth data available for countries.
Table S10. Multiple linear regression statistics with all three ANEW dimensions.
Table S11. Linear regression statistics for individual ANEW dimensions.
Table S12. Ordinary least squares linear regression statistics for sex-searches v.s. proximity in eigenmood to Christmas.
Table S13. List of words and expressions removed from the Twitter/ANEW analysis.

Supplementary Methods

S1. Notes on "misclassifications" for Country Classification from sex-searches

Some of the countries identified as Christian celebrate the nativity according to Julian calendar, with Christmas falling on January 7th or January 14th of the Gregorian calendar. Such is the case of the Christian countries: Belarus, Bosnia and Herzegovina, Georgia, Macedonia, Moldova, Montenegro, Serbia, Slovenia, Russia and Ukraine. Neither of these countries has a national holiday on December 25th nor shows an increase in sex-searches around December 25th. Had these countries been labeled as "Other", the percentage of countries identified as Christian for which we see a significant increase (z-score>1) in sex-searches would have been of 91%. In addition to not celebrating the Christmas on December 25th, some of these countries also have a sizeable percentage of population that self-identifies as Muslim. Such is the case of Montenegro (29%), Macedonia (39%) and Bosnia and Herzegovina (45%).

From the 30 Muslim countries, Pakistan was classified as Christian and 6 other countries didn't make the threshold. Pakistan is highly related to Christmas, probably due to the fact that there is a public holiday on 25th December, which coincidentally celebrates the birthday of Muhammad Ali Jinnah, founder of Pakistan. The other six countries also correspond to the ones for which the quality of the sex-search data was the poorest.

Keeping in mind that we were looking for countries that culturally relate to a Christian or Muslim religious background, all countries that didn't make the threshold to be labelled as either are classified as Other. Unsurprisingly, there are many countries who are originally labelled as Other and end up classified as either Christian or Muslim. European countries, such as the Czech Republic, Estonia and the Netherlands, whose majority does not identify as religious are classified as Christian, most likely due to the fact that these populations celebrate the holiday as well, even if secularly.

S2. Mean Sentiment Correlations with Sex-Search Volume

As shown in Supplementary Table S9A, there is a highly significant, moderate fit ($R^2 > 0.1$) across all countries, demonstrating a significant correlation between volume of sex-searches and mean sentiment as measured by the three ANEW dimensions. The coefficient of determination is generally stronger for Christian countries than Muslim Countries. Similarly to the GT data, the multiple linear regression models can be improved by averaging sentiment and sex-search volume across years using the 52-week Christmas centered calendar for the USA, Australia, Brazil, Argentina, and Chile, , and the 50-week Eid-al-Fitr centered calendar for Indonesia and Turkey. This smooths out extraordinary events that are picked up by sentiment analysis. The results of this centered-data regression are presented in Supplementary Table S9B. The fit is highly significant for all countries, and improves for all countries, ($R^2 > 0.26$). In every case, valence is yields a positive coefficient, while dominance a negative coefficient; so the happier but less dominant the sentiment expressed by a country, the more sex-searches tend to increase. As far as significance is concerned, t-tests reveal that the valence dimension is most often significant, followed by dominance, with arousal the least likely to be a significant factor.

Interestingly, as shown in Supplementary Table S10, when we computed the ordinary least squares estimate of a standard linear regression on each ANEW dimension independently, we obtained very poor (but significant) goodness of fit, as measured by R^2. Therefore, the mean value of each ANEW dimension on its own is a poor predictor of sex-search volume in all countries (with few exceptions such as Arousal in Brazil). We can thus say that mean sentiment correlates with sex-search volume (Supplementary Table S9) but the timeseries of mean weekly values of each ANEW dimension do not yield a nuanced characterization of sentiment correlated with interest in sex.

S3. Singular Value Decomposition

Singular value decomposition (SVD) is a method by which a matrix can be linearly decomposed into ordered orthonormal components, each explaining as much of the linear variation as possible, after the

components that came before it. The SVD of any m × n matrix M of real or complex numbers can represented as follows in Equation 2:

$$M=USV^T$$

Where U is an m× n matrix with orthonormal columns, V is an n× n matrix with orthonormal columns, and S is an n× n diagonal matrix. The columns of U and V are referred to as the left and right singular vectors of M respectively. These singular vectors are eigenvectors of the matrices MM^T and M^TM respectively. The diagonal entries of S, called the singular values of M, are the square roots of the eigenvalues of the matrices MM^T and M^TM. By convention, the singular values are ordered from greatest to least. The columns of U form a basis for the column space of M and the columns of V form a basis for the row space of M. The right singular vectors are also known in principal component analysis (PCA) as the loadings of the original variables (bins) onto the new coordinate system. The relative variance explained by each component can then be calculated for each component k as $s_k^2 / \sum_i (s_i^2)$ where s_k is the kth diagonal component of S. It is important to note that matrices can be reconstructed with a lower rank by setting elements of S to zero. Typically only the top *l* singular values are kept in order to reduce noise and create the closest rank-*l* approximation of the original matrix[19].

S4. Data Reconstruction

It can be clearly seen from the data reconstruction averages in Extended Data Fig. 8 and Supplementary Fig. S6, that the distribution of sentiment shifts towards higher bins during holidays, represented by redder high bins and greener low bins on holidays. Christmas stands out in the USA (US), Australia (AU), and Brazil (BR). Eid-al-Fitr stands out in both Turkey (TR) and Indonesia (ID), and in Turkey the beginning of Ramadan is emphasized a few weeks before. The centering performed only looks at weeks within the surrounding cultural year, such that Christmas is week 26 of a 52 week year (starting with a first week 1), while Eid-al-Fitr is week 25 of a 50 week year. Other weeks are averaged in this range according to their displacement from the holiday week (e.g., a week two weeks before the Christmas week in 2012 is averaged with weeks two weeks before Christmas in all other years). This obscures the emphasis on holidays using another calendar, such that Indonesia also has a strong signal on Christmas, but these signals are averaged over multiple weeks when the calendars are misaligned. The heatmaps for all countries centered on all holidays are included in Supplementary Fig. S6.

S5. Eigenmood Selection and Characterization

The mean value of a holiday’s projection on various components for different countries are shown in Supplementary Figures S2 and S3 for Christmas and Eid-al-Fitr respectively, with the two components selected for each country highlighted in red. As described, since the first component corresponds to the basic distribution of sentiment in the language and overwhelms projections because of how much it explains, and the last few components are mostly noise, we only look at the components explaining 95% of the variance after the removal of the first. The second component usually describes a variation over the whole time series of out data, thus it tends to have a large standard deviation.

To better understand how the selected components describe the mood, we define an interpretable linguistic variable[29]. The linguistic variable can take five fuzzy values, ”low”, ”medium-low”, ”medium”, ”medium-high”, and ”high” with membership functions defined over the 25 bins of the original twitter sentiment distribution. These membership functions are shown in Supplementary Fig. S4 and were chosen such that each original bin’s membership in all values sums to one, and the area under each membership function is the same.

The response of the linguistic variable to the holiday in each selected eigenmood is shown in Supplementary Figure S5 for the selected relevant holiday for each country. These responses were calculated by reconstructing the distribution bins with only the eigenmood selected for the country and holiday, multiplying the reconstructed bin value by its memberships, and summing over all bins for each linguistic value. These responses can be interpreted as the change from the language’s base sentiment distribution on the holiday contributed by the selected eigenmood. The response characterized by the Christmas eigenmood in the USA is an increase in

medium-high happiness, with decreases in other levels of happiness, low and medium happiness in particular. How mood changes on a major holiday varies between countries but generally we see that the selected eigenmood describes increases medium-high or high valence on the holidays, with decreases in low, medium-low, and medium valence, as well as lower or more moderate dominance and arousal. The behavior of the dominance mood dimension in the week of Eid-al-Fitr in Indonesia highlights the importance of the more nuanced mood measurement that eigenmoods afford. While the ANEW mean value measurement above suggested a dominance decrease towards a less “in-control” mood, what we have at Eid-al-Fitr is a shift away from the extremes to a collective mood state that is neither very “in-control” nor very “controlled” – coherent with a happier and calmer mood scenario typically found in these holidays for all countries. In other words, during most weeks of the year, there is increased bimodal dominance activity in higher and lower bins (simultaneously high “in-control” and “controlled”, respectively), but in the week of Eid-al-Fitr, the dominance mood converges to a mid-level dominance (Figure 4 column A, row 3, dominance panel).

S6. Eigenmood correlations to Sex-search volume in target Holidays

As a measure of mood similarity between weeks in a space defined by a selected eigenmood, we use the dot product between their coordinates in this space[20]. This measure increases between weeks with similar (positive or negative) projections onto the eigenweeks forming the space, becomes negative with opposite projections, and decreases in magnitude with weeks that are not correlated with the eigenweeks and are thus projected near the origin. Due to the properties, it is important to select an eigenmood that strongly corresponds to a week or weeks of interest, by containing high-magnitude values in the corresponding eigenbins. The similarity can then be expressed as $w \cdot c$ where w and c are weeks projected into the eigenmood, which is equivalently the vector of corresponding weighted eigenbin values. In comparison between weeks and a holiday averaged over years, these vectors are the element-wise averages of the week’s projection coordinates over the years. We report results with these averages, but these results are robust to yearly, non-averaged data, as well as different selection criteria for the eigenmoods (for example, allowing a greater number of components). The projection spaces for each eigenmood are shown in Supplementary Fig. S7.

In general, weeks close in proximity in time will be more similar in eigenmood, but certain weeks, often other holidays, more distant in time can have a high similarity in eigenmood to the selected holiday. In the USA, for example, the weeks closest in eigenmood to Christmas are, in order, the week of New Year’s Day, the other weeks of December, and the weeks following July 4th, Father’s Day, and Memorial Day. National Day in Chile is similar in eigenmood and sex searches to Chile’s Christmas. New Year’s Day and Christmas in Indonesia are similar to Eid-al-Fitr’s eigenmood and high sex searches. In Turkey, weeks in late June, early July, and the week following Eid-al-Fitr are the most similar in terms of eigenmood and sex search volume to Eid-al-Fitr.

To investigate the relationship between a week’s similarity in eigenmood to a holiday and the number of sex searches, we perform an ordinary least squares regression between sex searches as the dependent variable, and similarity as the independent variable. Displayed in Figure 4 and reported in Extended Data Table 2 are the results of this regression as well as Brownian distance correlation statistics, a nonlinear measure of correlation[30]. The plots of all linear regressions are included in Supplementary Fig. S7.

There is a fairly strong correspondence ($R^2 \geq .380$) between similarity in eigenmood to Christmas and sex searches in the C countries: the US, Brazil, Australia, Argentina, and Chile. The southern hemisphere Christian countries Brazil, Argentina, and Chile also have a noticeable correlation with Eid-al-Fitr, however, the slope of the regression is negative, implying that the less like the mood during the winter week of Eid-al-Fitr, the more sex searches are conducted.

In Muslim countries Turkey and Indonesia, we were limited by having less Twitter data and fewer tweets that match. However, there are significant correlations between similarity to Eid-al-Fitr and increased sex searches. The linear correlation is reduced compared to Christmas in Christian countries, since over time the weeks of Ramadan become more similar in eigenmood to Eid-al- Fitr, the festival at Ramadan’s conclusion, while

the cultural pressure is one of abstinence, such that these weeks have unusually low sex searches. In the case of Turkey in particular, the holiday of Eid-al-Adha, or the Sacrifice Feast, also has high sex searches, but is different in eigenmood from Eid-al-Fitr. The positive correlation between sex searches and Christmas eigenmood in Indonesia is likely caused by the sizable Christian population living there and effects due to summer.

Turkey is an interesting case, since it has a very strong negative correlation between sex searches and similarity to Christmas although the response to Eid-al-Fitr is smaller. In part, this may be due to limitations in our data gathering and method application, since our ANEW is only available in English, Spanish, and Portuguese. However, we still have a good number of tweets from Turkey, so we look more closely at its eigenmood. The projection of all weeks into its eigenmoods for Christmas and Eid-al-Fitr is shown in Supplementary Fig. S7, which happen to be same in this case. The regressions between sex searches and the similarity of averaged weeks to Christmas and Eid-al-Fitr are shown in Supplementary Fig. S7. The mood associated with Eid is also associated with Ramadan, which emphasizes abstinence. During the weeks of Ramadan, there are much fewer sex searches than usual, although the weeks are not too far different in mood. In addition, there is a separate holiday, Eid-al-Adha, that is associated with a second peak in sex searches, but with a different mood. Perhaps due to Turkey's small Christian population and winter timing, Christmas and weeks like it in eigenmood have low sex searches and averaging over years decreases the effects of holiday traditions (like Eid-al-Fitr) due to misaligned calendars.

Supplementary Figures

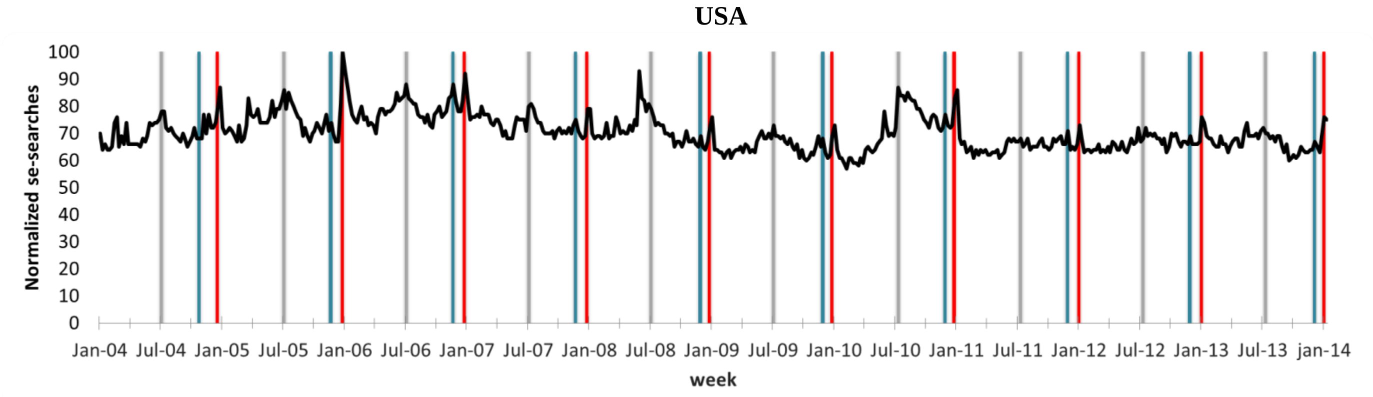

Fig. S1A**. GT query [sex] results for the USA.** The weeks containing Thanksgiving day, Christmas and the 4th of July are highlighted in blue, red and grey, respectively.

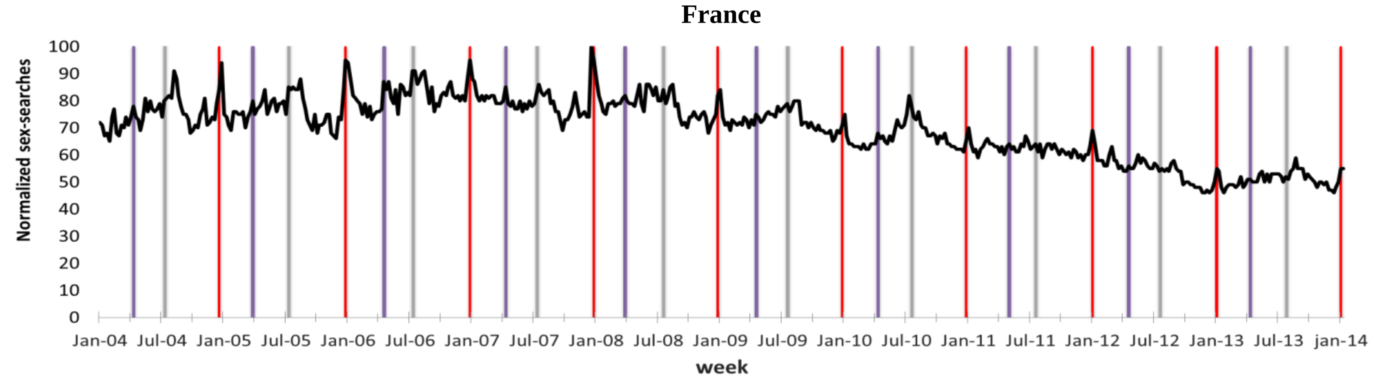

Fig. S1B**. GT query [sex] results for France.** The weeks containing Easter Sunday, July 14th and Christmas are highlighted in purple, grey and red, respectively.

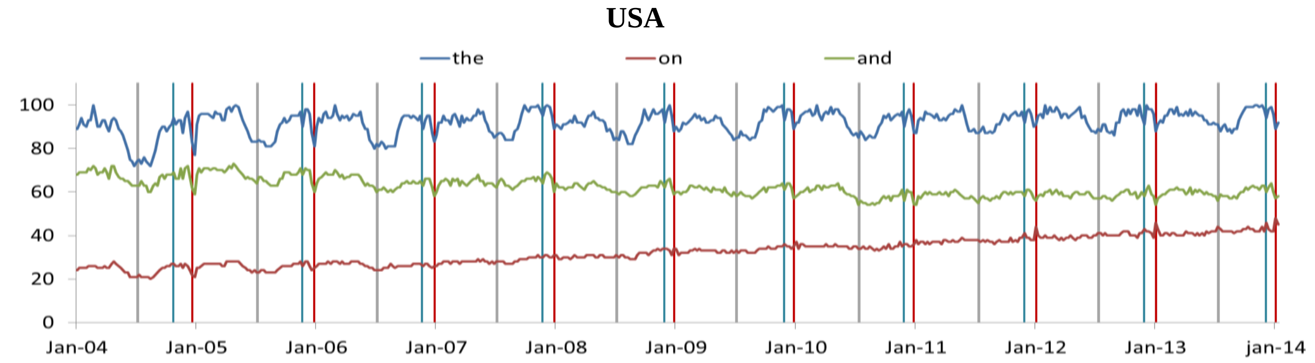

Fig. S2A**. GT queries for "the", "on" and "and", in the USA.** The weeks containing Thanksgiving day, Christmas and the 4th of July are highlighted in blue, red and grey, respectively.

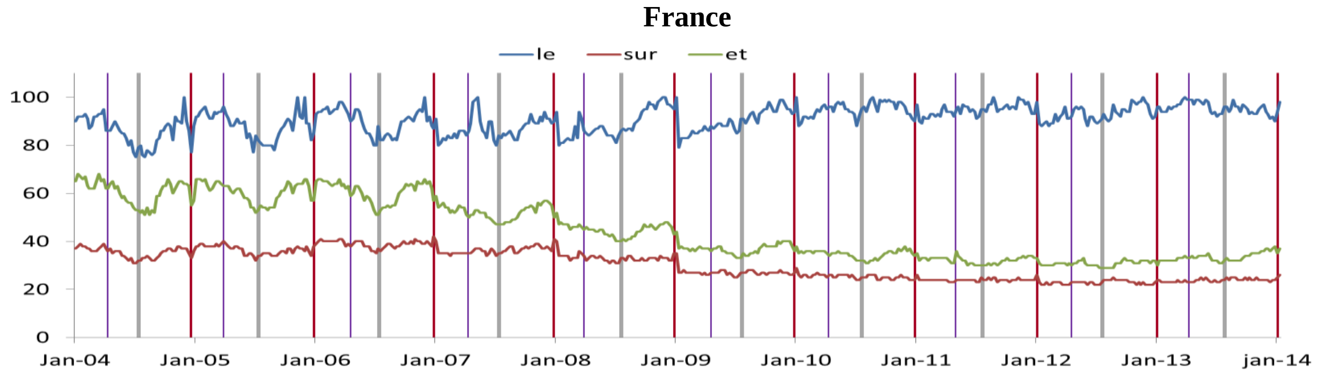

Fig. S2B**. GT queries for "le", "sur" and "et", in France.** The weeks containing Easter Sunday, July 14th and Christmas are highlighted in purple, grey and red, respectively.

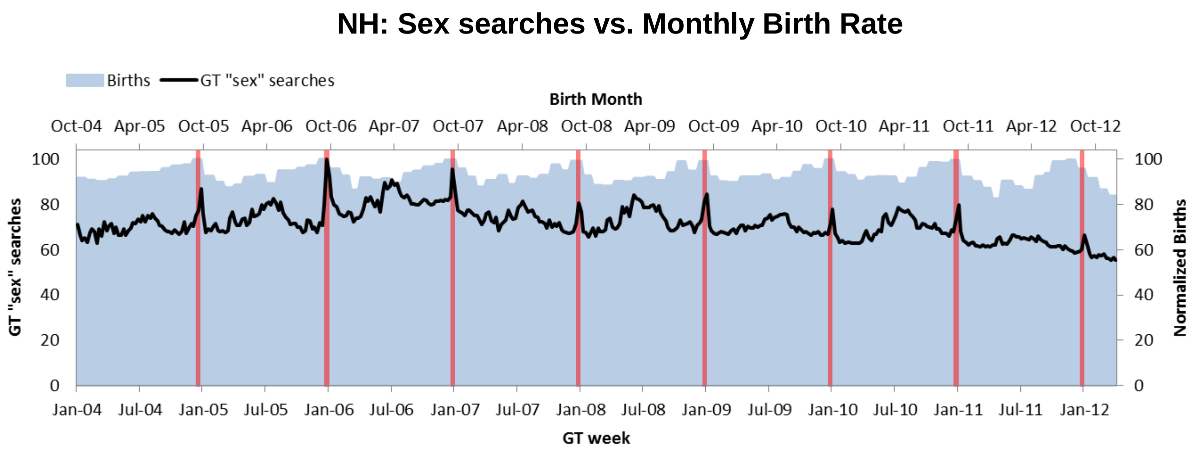

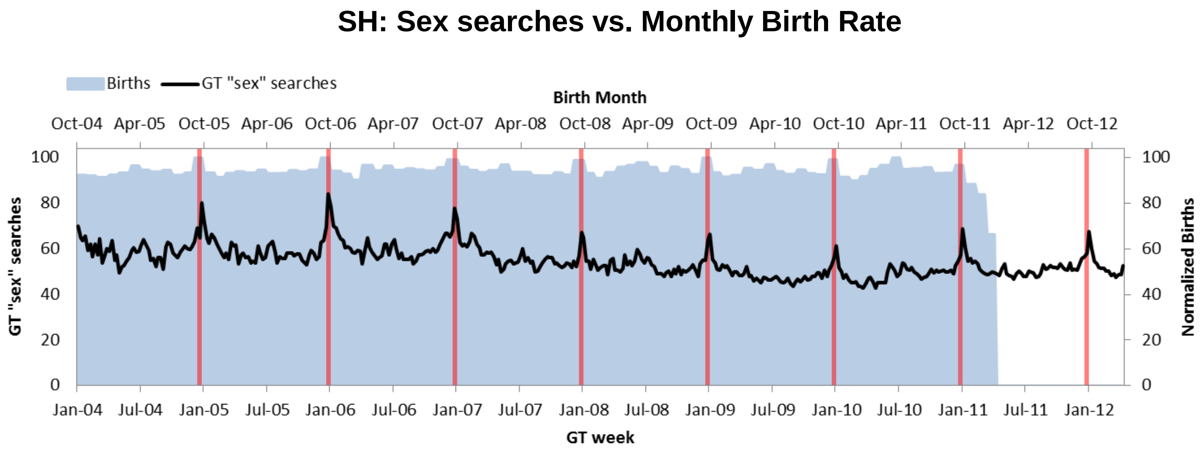

Fig. S3. Monthly birth data shifted by nine months (blue shaded area, top and right axis) and weekly averaged Google Trends results for “sex-searches” (black line, bottom and left axis) plotted for:
A) All Western Northern countries for which both birth and GT data exist (Austria, Canada, Denmark, Finland, France, Germany, Italy, Lithuania, Malta, Netherlands, Poland, Portugal, Spain, Sweden and United States of America), also represented in Fig. 1 in the main paper. Births in September are higher than the yearly average in all countries but Lithuania and Sweden, with an average variation of 6%).

B) All Southern countries for which both birth and GT data exist (Australia, New Zealand, Chile and South Africa). Births in September are higher than yearly average in all countries (average variation 5.5%, with the difference being as high at 10% in South Africa and New Zealand.)

Births were shifted nine months to match probable conception month. The red line marks Christmas week.

Sex searches by hemisphere classification

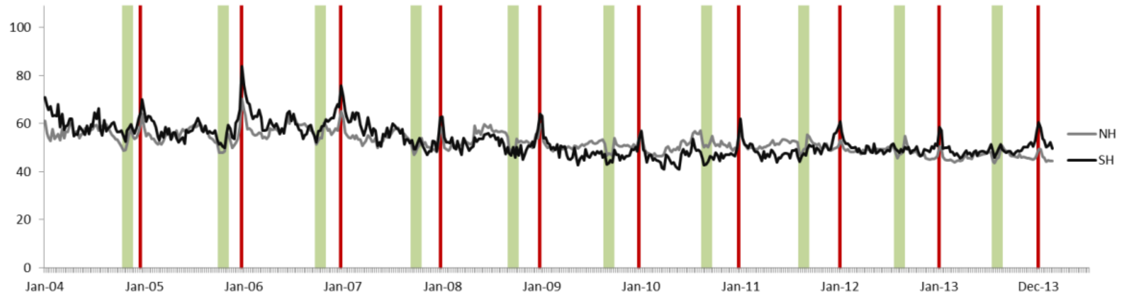

Fig. S4A. Averaged sex-searches for Northern and Southern countries. R^2 is 0.54 with a p-value of 2E-41. The weeks containing Ramadan and Christmas Day are highlighted in green and red, respectively.

Sex searches by cultural classification

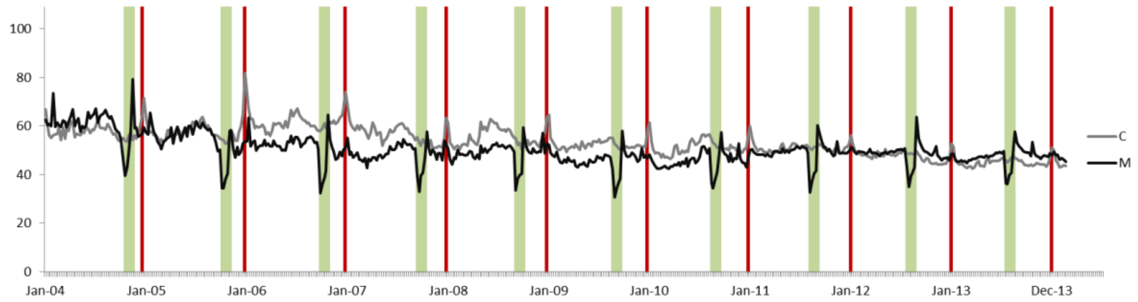

Fig. S4B. Averaged sex-searches for all Christian and Muslim countries. R^2 is 0.19 with a p-value of 3E-26. The weeks containing Ramadan and Christmas Day are highlighted in green and red, respectively.

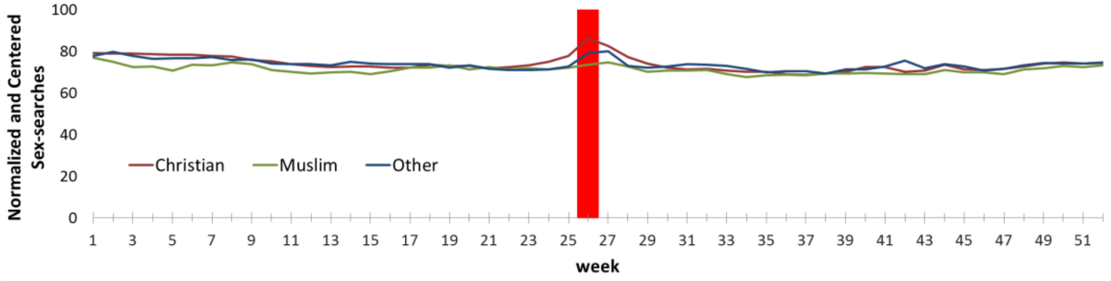

Fig. S5A. Averaged Christmas-centered results for the Christian (red), Muslim (green) and Other (dark blue) country sets. The red vertical bar represents the Christmas week, centered on week 26.

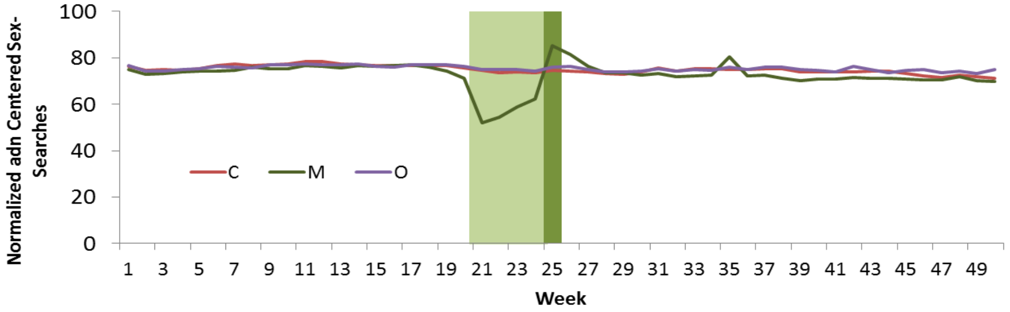

Fig. S5B. Averaged Eid-al-Fitr-centered results for the Christian (red), Muslim (green) and Other (dark blue) country sets. The darker green vertical bar represents the Eid-al-Fitr week, centered on week 25. The light green area represents the remaining Ramadan weeks.

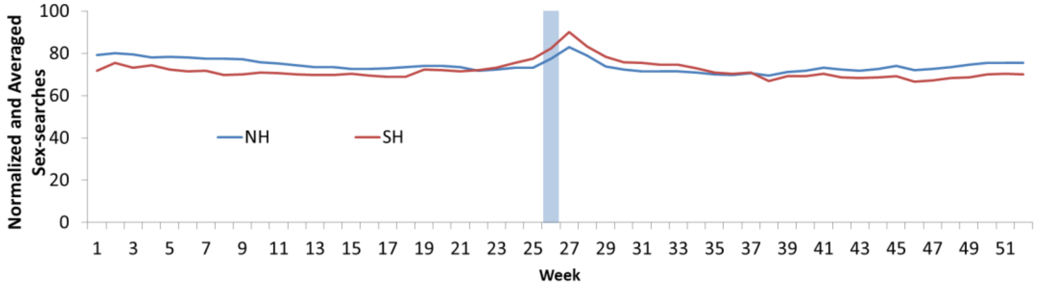

Fig. 5C. Averaged December Solstice-centered results for the Northern Hemisphere (blue) and Southern Hemisphere (red) country sets. Light blue vertical bar represents the week of the December-Solstice.

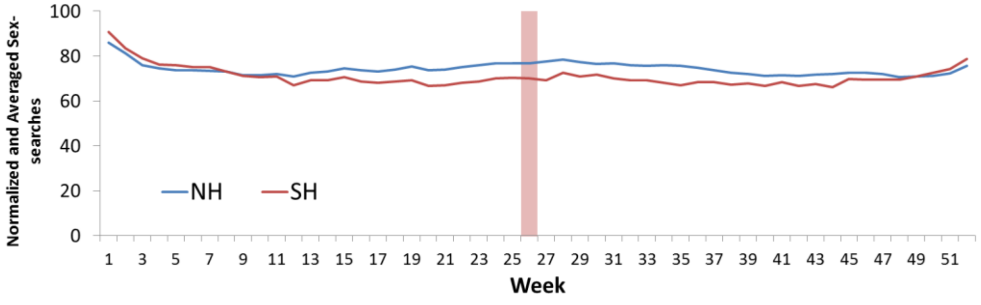

Fig. S5D. Averaged June Solstice-centered results for the Northern Hemisphere (blue) and Southern Hemisphere (red) country sets. Light pink vertical bar represents the week of the June-Solstice.

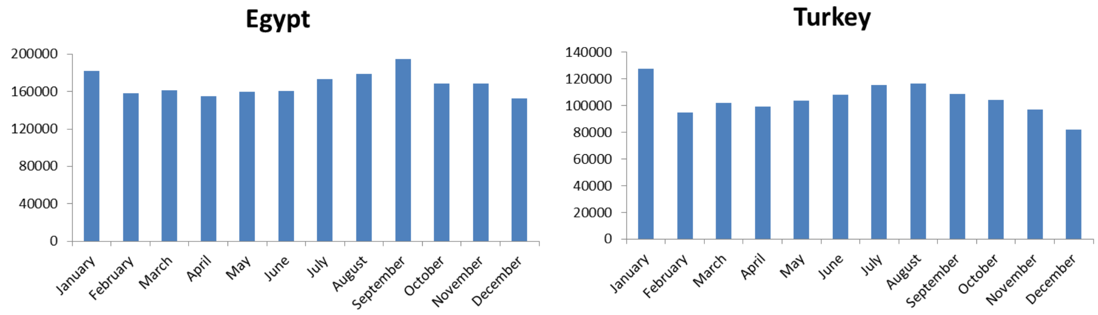

Fig. S6A. Averaged monthly births (for all available years) for Turkey and Egypt. In some Muslim countries, as in these examples, birth records are artificially at their lowest in December (in the case of Turkey, 22% below average) and peak in January (in the case of Turkey, 202% above average) , as parents prefer to have their children registered in the New Year.

Turkey

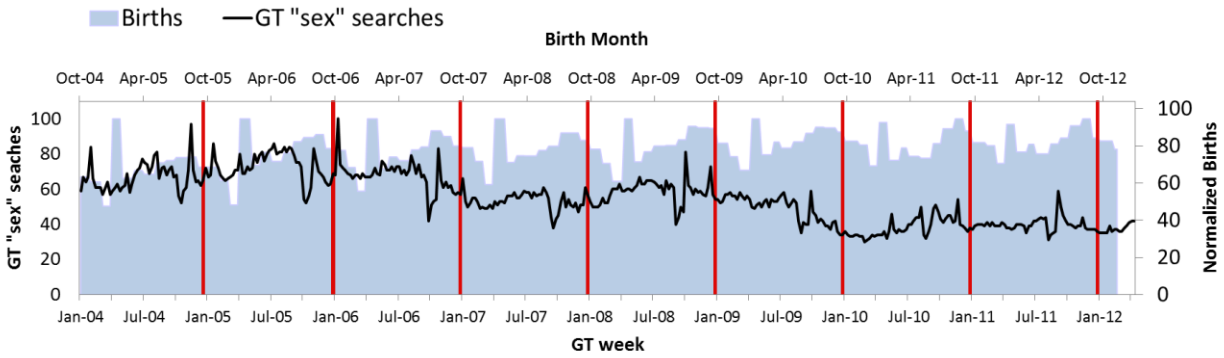

Fig. S6B Normalized monthly birth data (shaded blue, top and right axis) and Google Trends results of “sex”-searches (black line, left and bottom axis) for Turkey. Births were normalized so that each year’s maximum becomes 100 and shifted nine months to match with probable conception month. The red line represents Christmas week, which was very close to Eid-al-Ada in 2005, 2006 and 2007. (It is obvious that the major registration peak happens in January of each year and it’s not matched by an increase in sex-searches).

A) Russian and Serbian Orthodox Countries

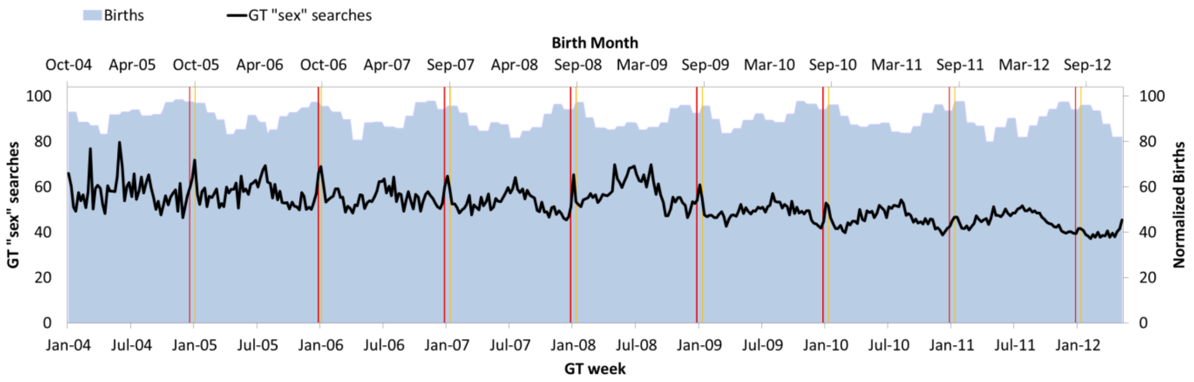

B) South Korea

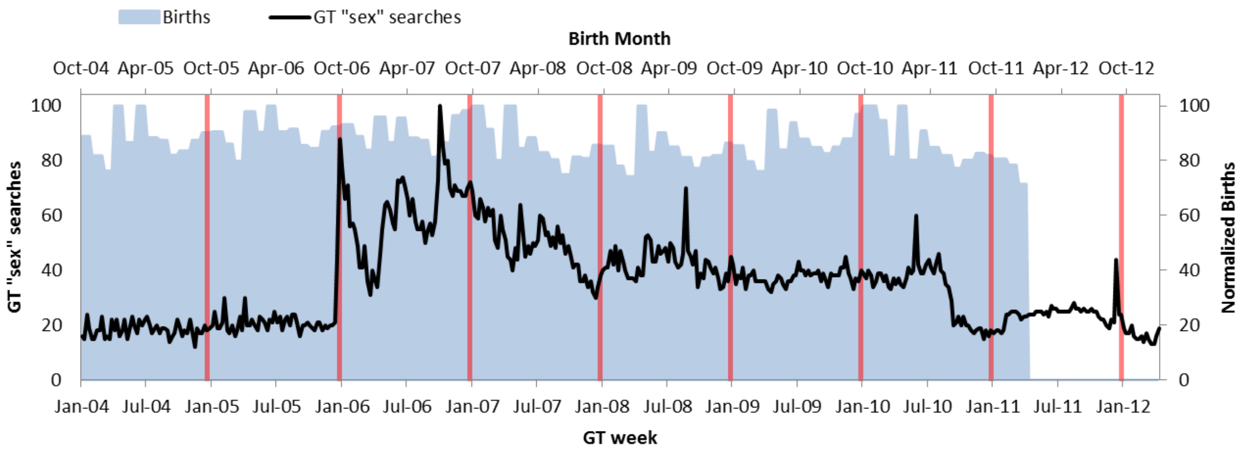

Fig. S7. Normalized monthly birth data (shaded blue, top and right axis) and Google Trends results of ”sex”-searches (black line, right and bottom axis) for
A) All Northern and Christian countries for which both birth and GT data exist that Celebrate Christmas on January 6th (Macedonia, Moldova, Serbia, Slovenia, Russia and Ukraine).
B) South Korea, as an example of a Northern Other country, for which both birth and GT data exists.

Births were shifted nine months to match with probable conception month. Vertical lines represent Christmas week with red marking the week of December 25th and orange marking the week of January 6th.

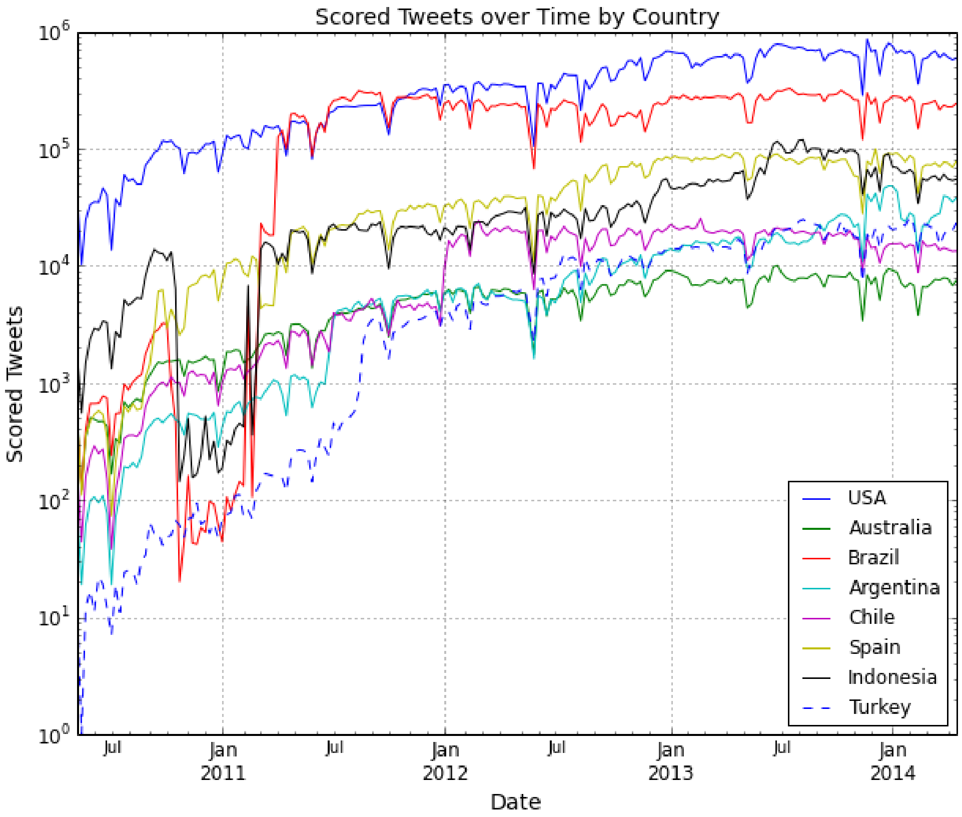

Fig. S8. Total number of weekly geolocated tweets matching ANEW for countries selected for *Eigenmood* analysis.

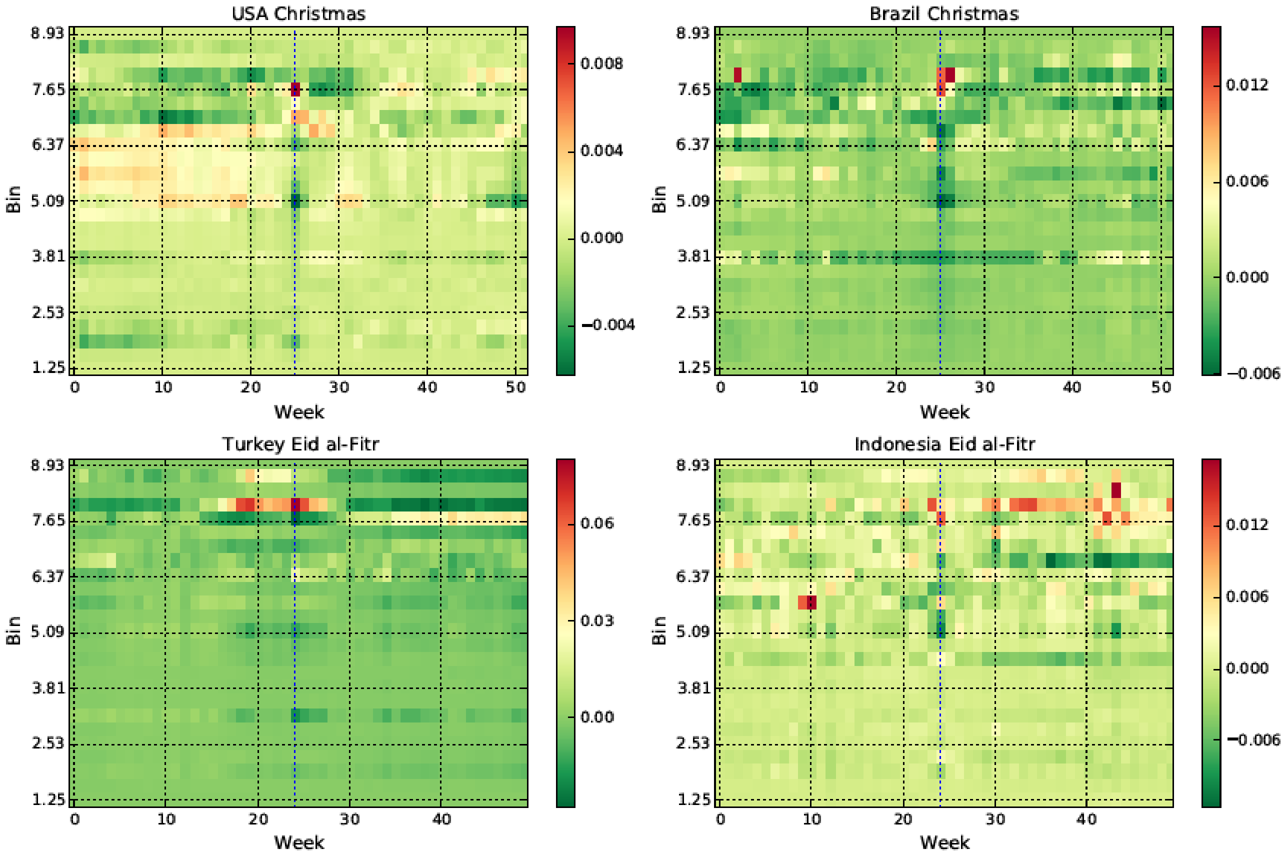

Fig. S9. Reconstructed valence heatmaps for multiple countries, centered on cultural holidays. Probability distributions of tweet valence were arranged in 25 bins (y-axis) each week (x-axis) for each country. Years were centered on a chosen holiday, marked by a central, vertical line. These data were averaged over all years, so each cell contains the average probability of a tweet's valence falling into a bin during a week. The data were reconstructed by removing the first component and components explaining less than 95% of the remaining variance.

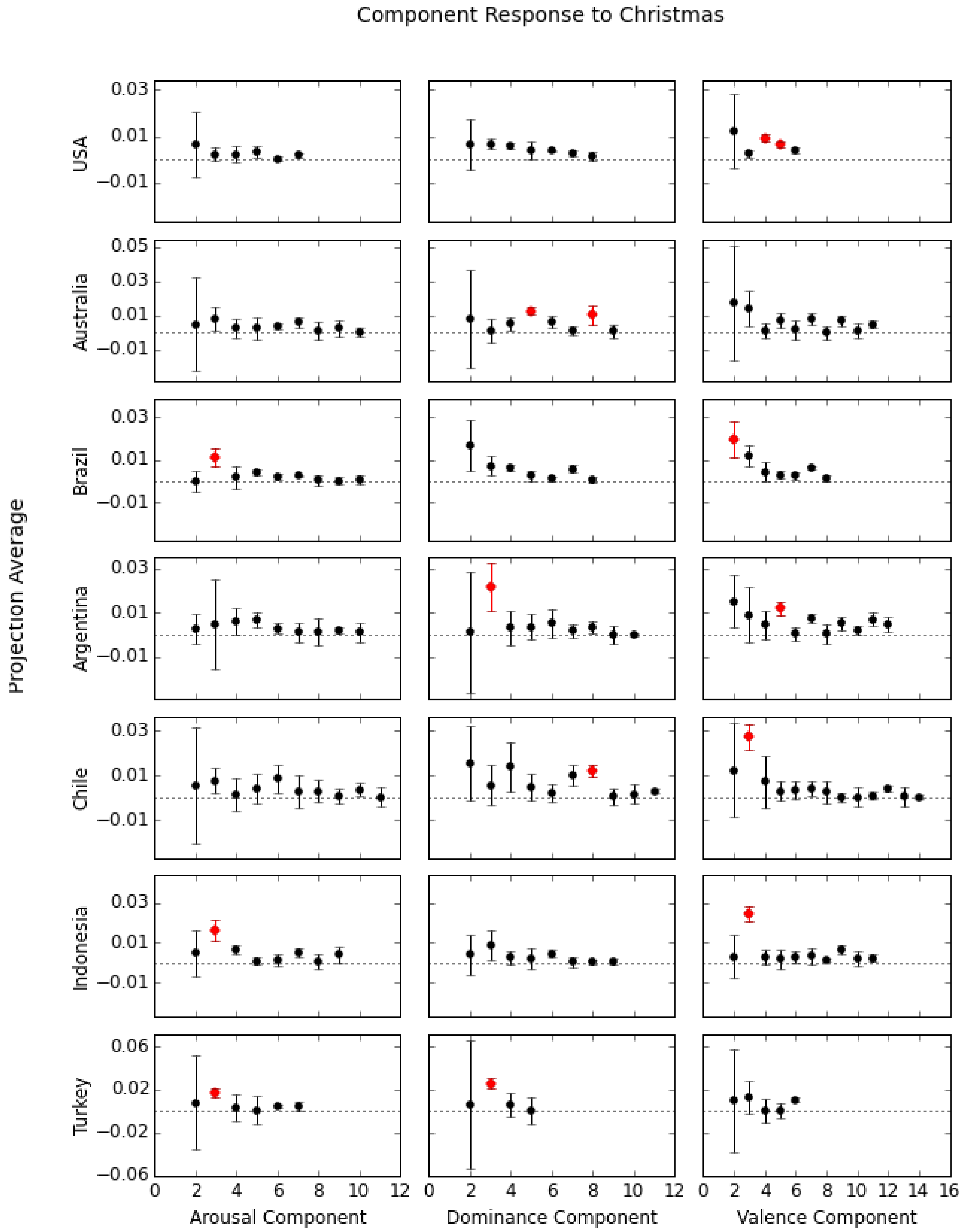

Fig. S10. ANEW component response to Christmas by country. Selected components highlighted in red.

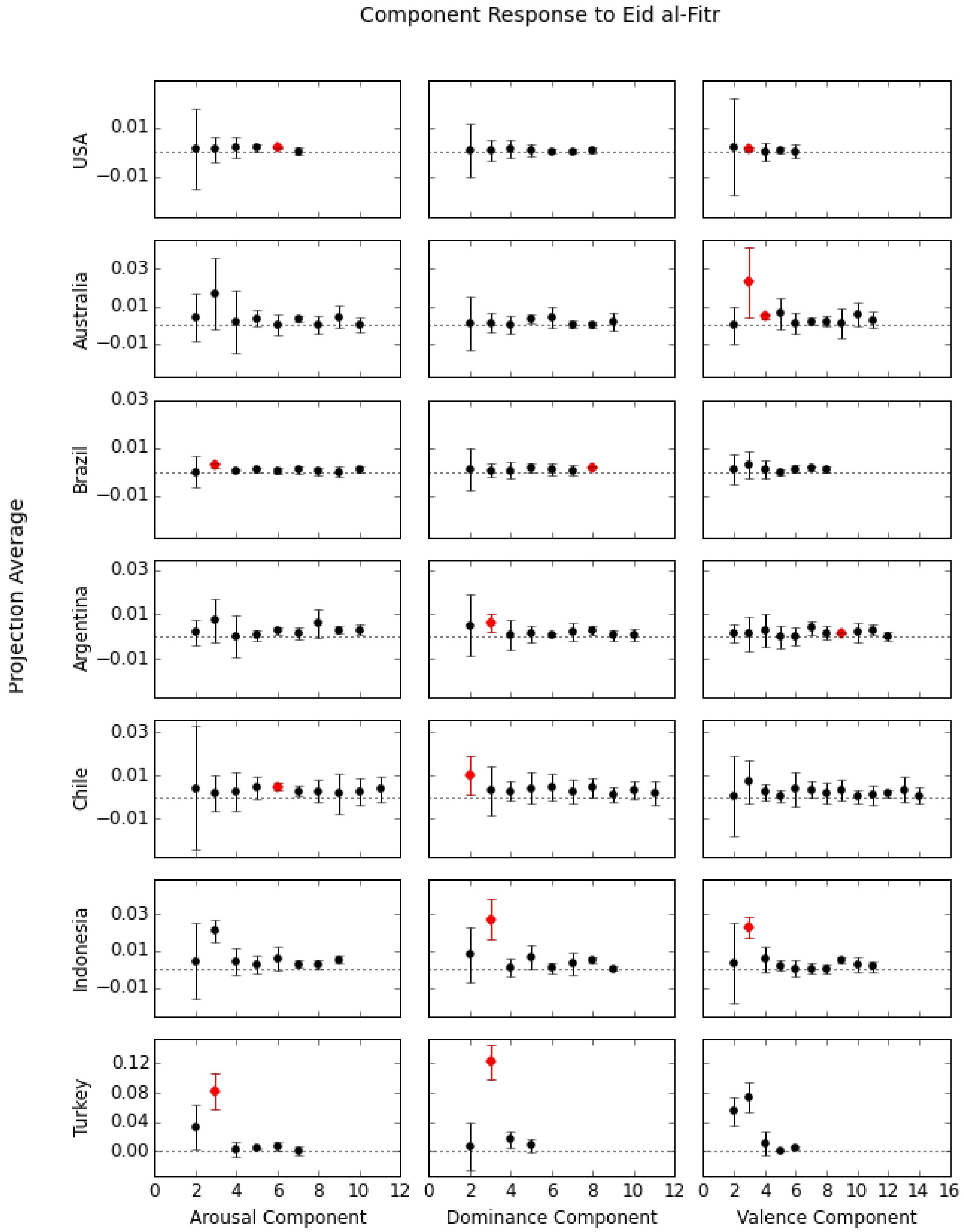

Fig. S11. ANEW component response to Eid-al-Fitr by country. Selected components highlighted in red.

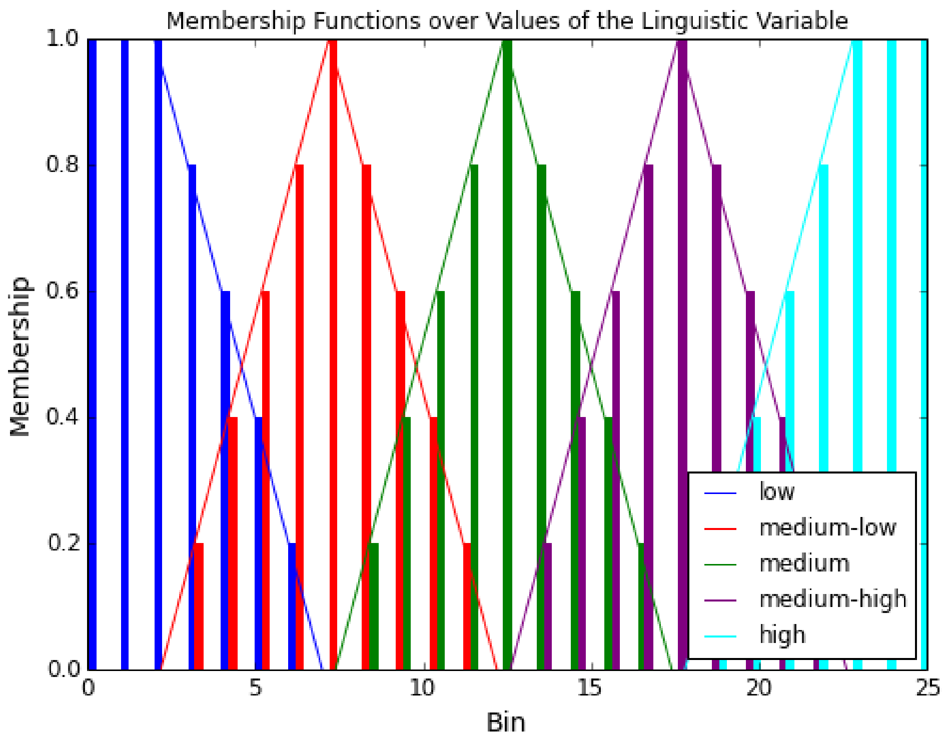

Fig. S12. Linguistic Variable value membership functions over 25 bins. The original bins belong to the values of the linguistic variable ("low", "medium-low", "medium", "medium-high", "high") to different extents. The membership functions are mappings from the original bins to a value between 0 and 1, representing membership fuzzy value of that the linguistic variable can take. The membership functions were chosen such that the sum of a bin's membership across all functions is 1, and the area under each membership function's curve is equal.

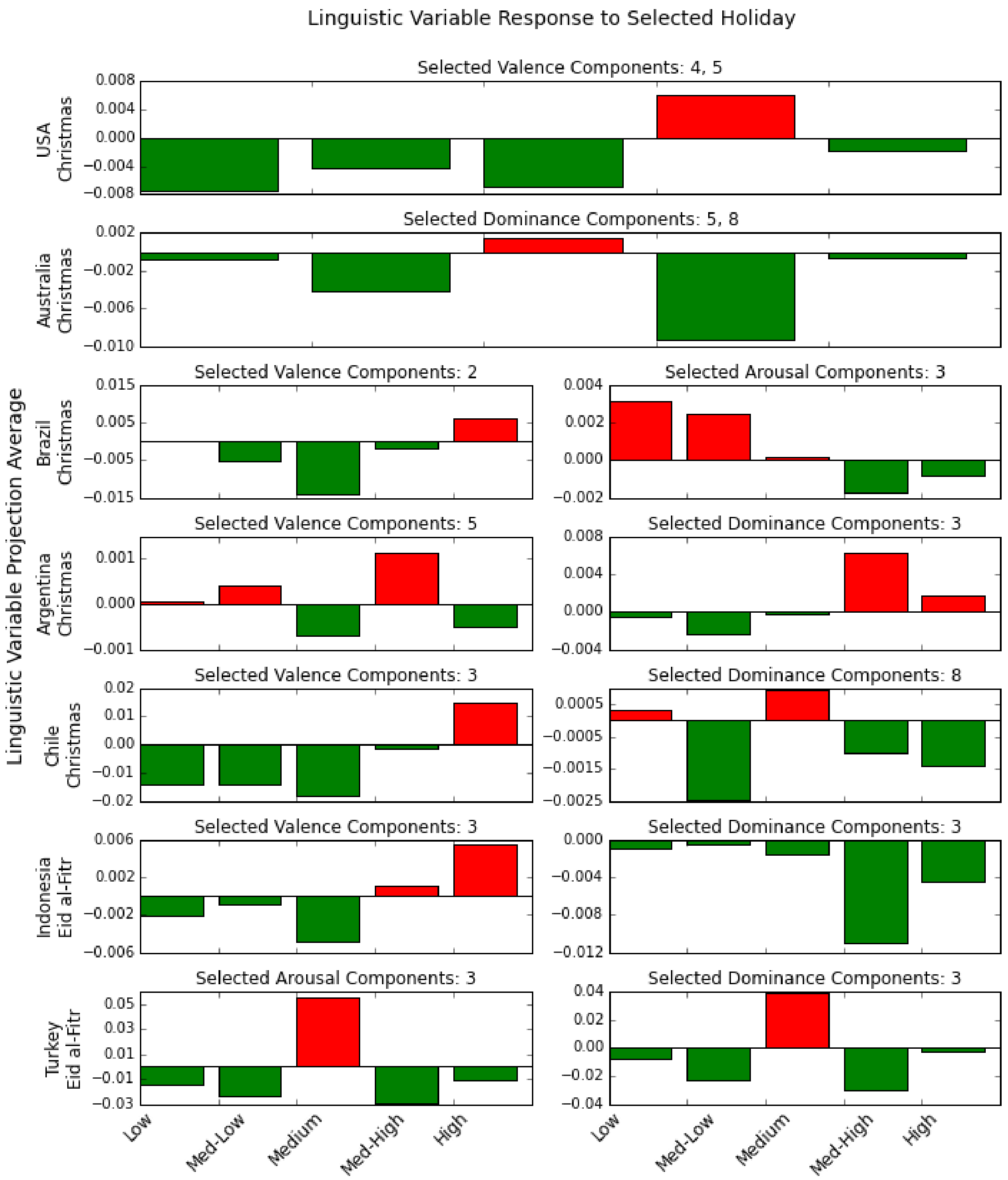

Fig. S13. Linguistic Variable Response to relevant holidays selected for each country, as an aid to interpret the effect of chosen *eigendays* during the holidays. A positive value (in red) means that the members of that value of the linguistic variable had increased weight on the holiday, while negative (in green) means they had decreased weight on the holiday.

Fig. S14. Average year reconstructed heatmaps. Reconstructed valence heatmaps for each country's average year centered on different holidays. Distributions over time are reconstructed from the components that explain 95% of the variance in the data after the first component is removed. Green represents a decrease in the bin compared to the full distribution, red represents an increase, and yellow represents no change. Center dotted line is the holiday of interest. Left: Christmas, Right: Eid-al-Fitr. Countries top to bottom: USA, Australia, Brazil, Argentina, Chile, Indonesia, Turkey

Arousal

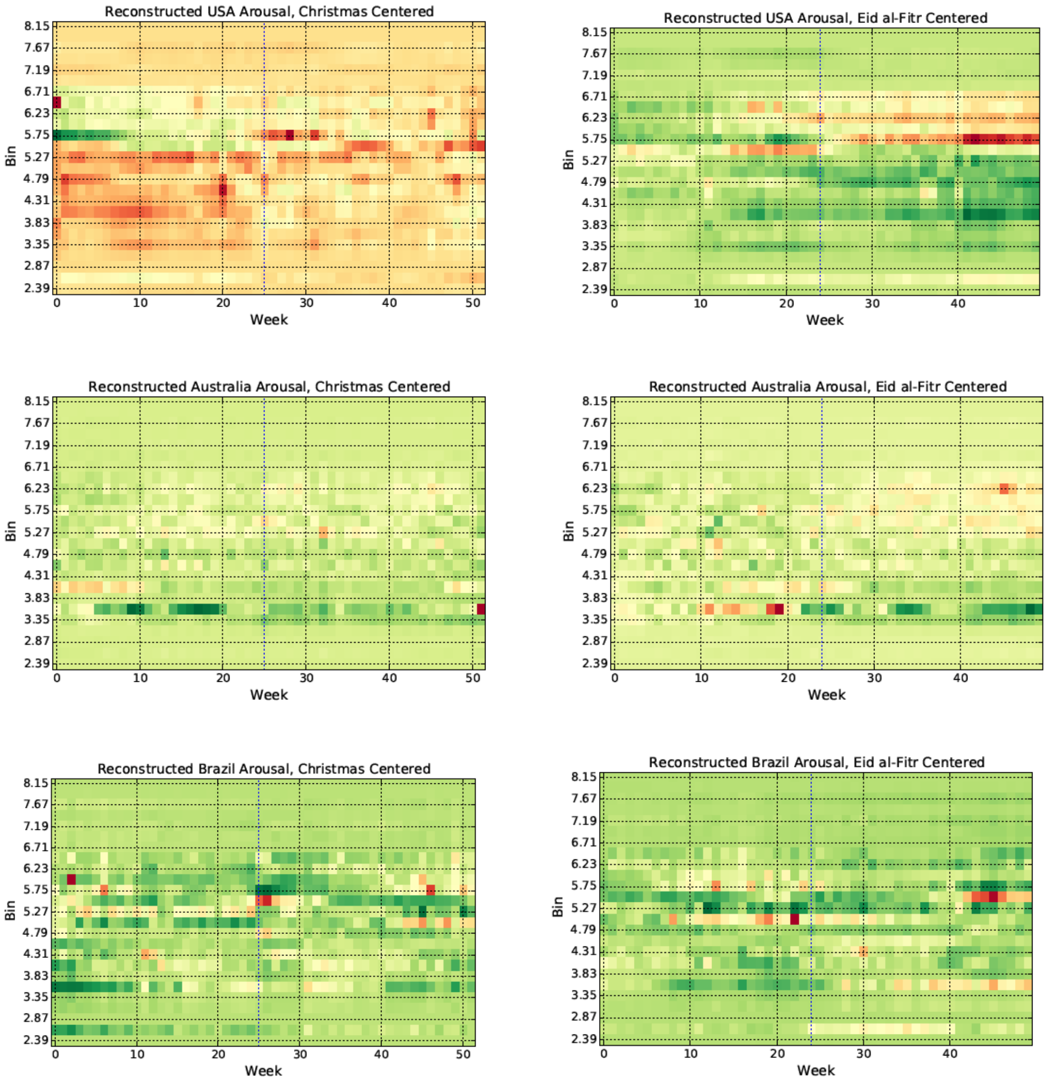

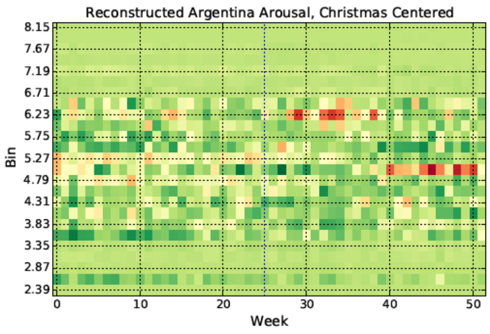
Reconstructed Argentina Arousal, Christmas Centered
Bin
Week

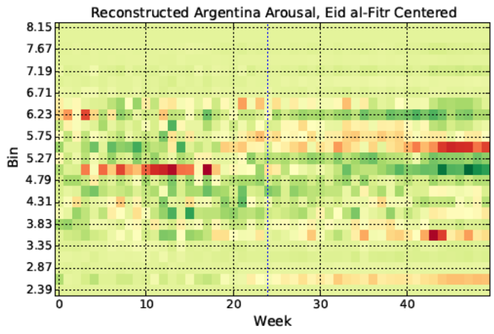
Reconstructed Argentina Arousal, Eid al-Fitr Centered
Bin
Week

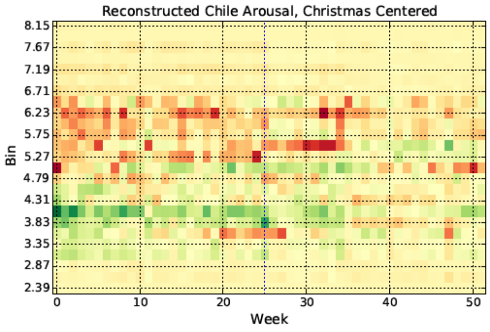
Reconstructed Chile Arousal, Christmas Centered
Bin
Week

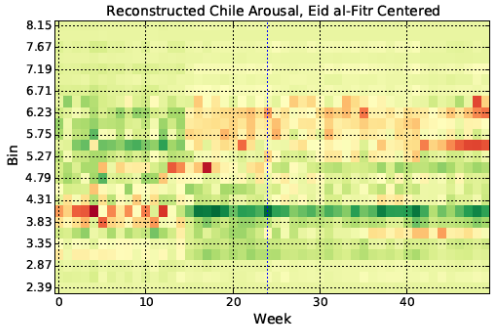
Reconstructed Chile Arousal, Eid al-Fitr Centered
Bin
Week

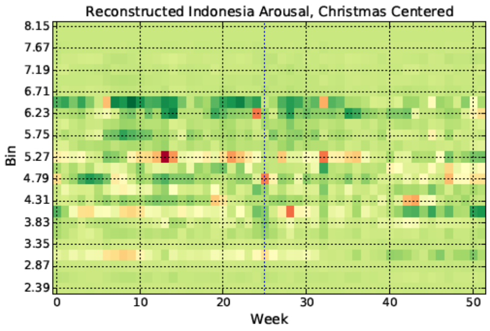
Reconstructed Indonesia Arousal, Christmas Centered
Bin
Week

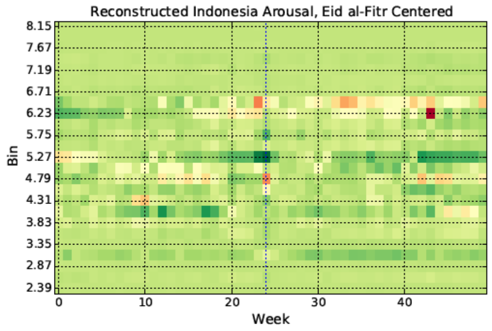
Reconstructed Indonesia Arousal, Eid al-Fitr Centered
Bin
Week

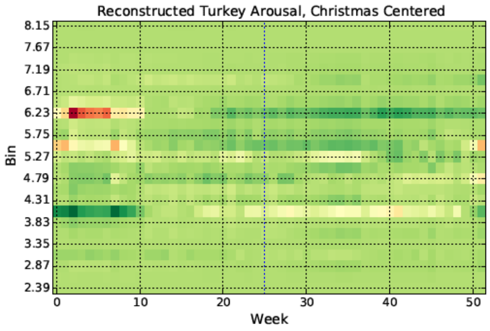
Reconstructed Turkey Arousal, Christmas Centered
Bin
Week

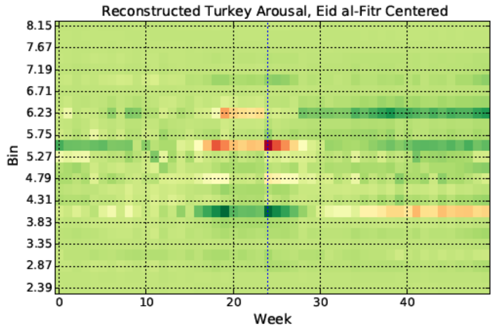
Reconstructed Turkey Arousal, Eid al-Fitr Centered
Bin
Week

Dominance

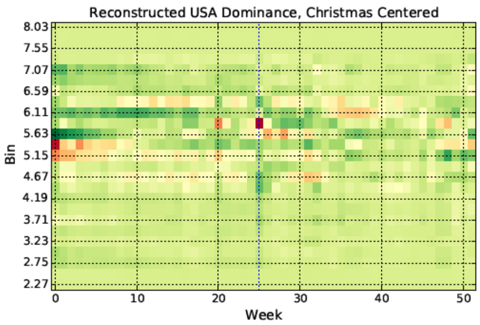
Reconstructed USA Dominance, Christmas Centered
Bin
Week

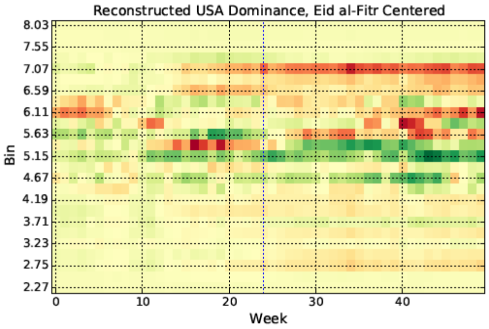
Reconstructed USA Dominance, Eid al-Fitr Centered
Bin
Week

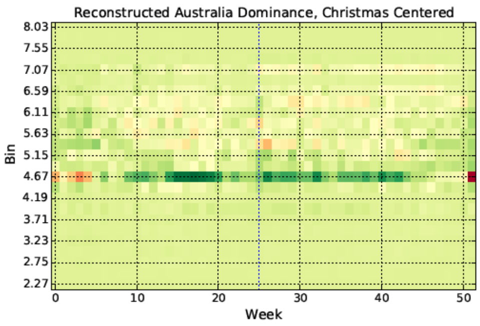
Reconstructed Australia Dominance, Christmas Centered
Bin
Week

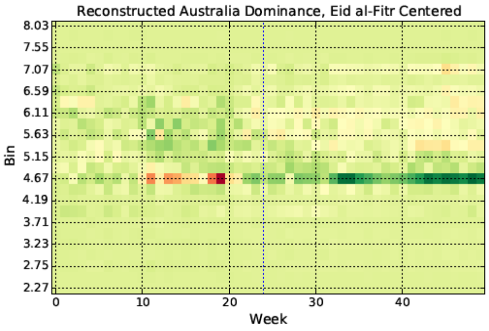
Reconstructed Australia Dominance, Eid al-Fitr Centered
Bin
Week

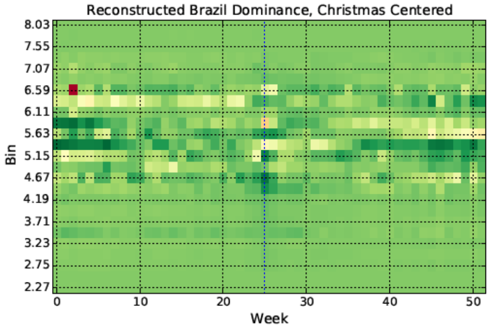
Reconstructed Brazil Dominance, Christmas Centered
Bin
Week

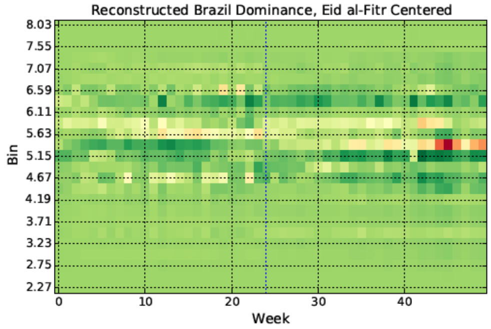
Reconstructed Brazil Dominance, Eid al-Fitr Centered
Bin
Week

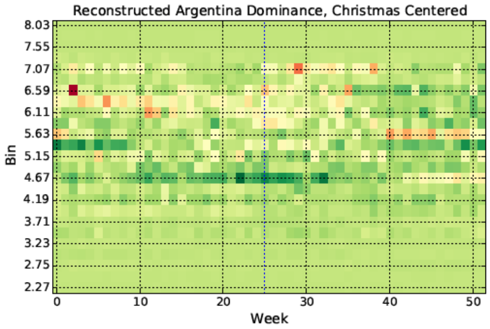
Reconstructed Argentina Dominance, Christmas Centered
Bin
Week

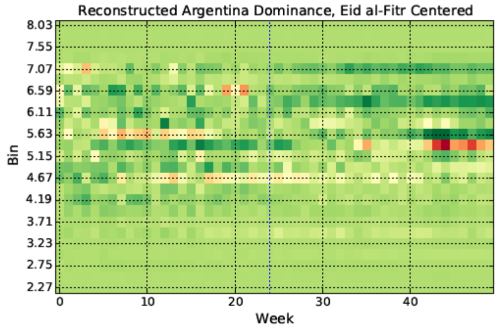
Reconstructed Argentina Dominance, Eid al-Fitr Centered
Bin
Week

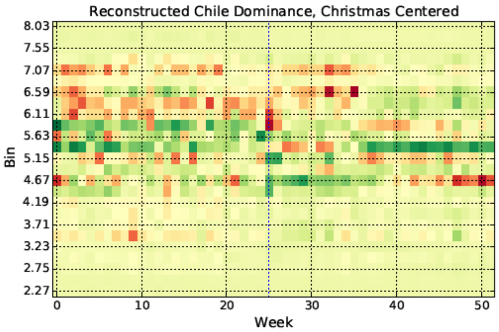
Reconstructed Chile Dominance, Christmas Centered
Bin
Week

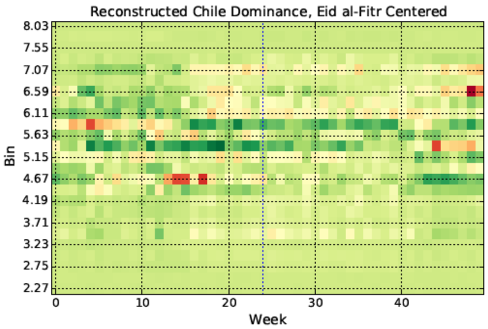
Reconstructed Chile Dominance, Eid al-Fitr Centered
Bin
Week

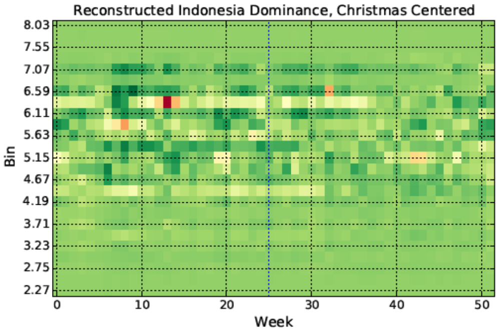
Reconstructed Indonesia Dominance, Christmas Centered
Bin
Week

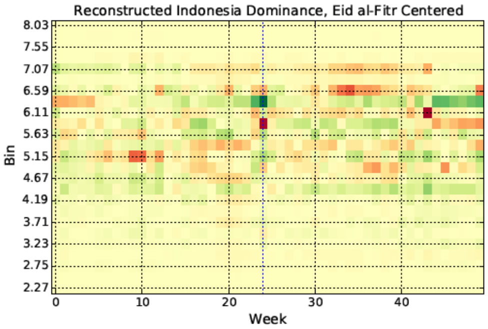
Reconstructed Indonesia Dominance, Eid al-Fitr Centered
Bin
Week

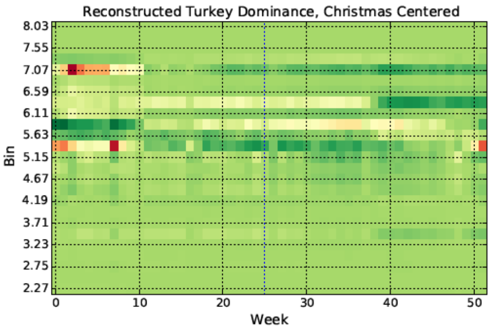
Reconstructed Turkey Dominance, Christmas Centered
Bin
Week

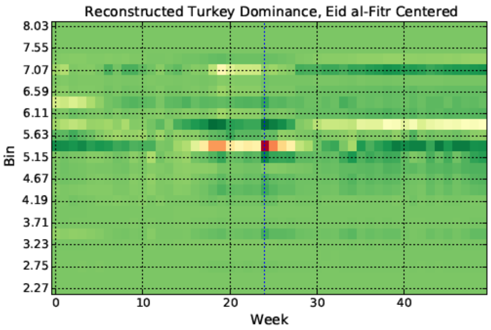
Reconstructed Turkey Dominance, Eid al-Fitr Centered
Bin
Week

Valence

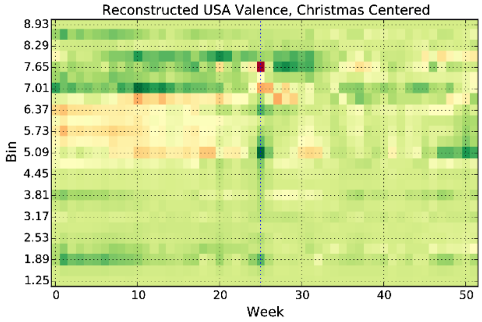
Reconstructed USA Valence, Christmas Centered
8.93 8.29 7.65 7.01 6.37 5.73 5.09 4.45 3.81 3.17 2.53 1.89 1.25
Bin
0 10 20 30 40 50
Week

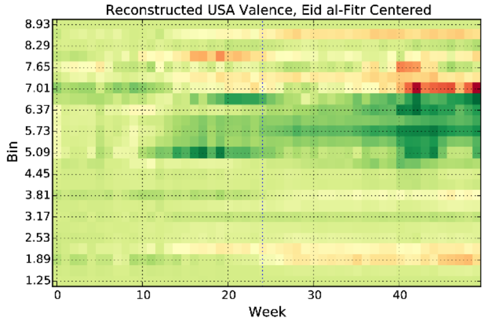
Reconstructed USA Valence, Eid al-Fitr Centered
8.93 8.29 7.65 7.01 6.37 5.73 5.09 4.45 3.81 3.17 2.53 1.89 1.25
Bin
0 10 20 30 40
Week

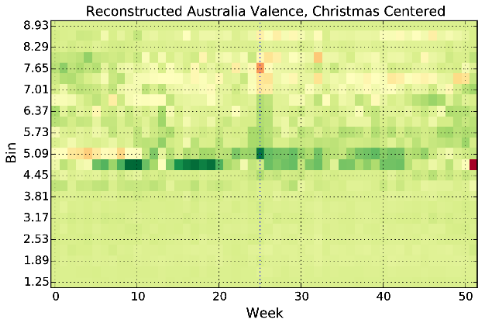
Reconstructed Australia Valence, Christmas Centered
8.93 8.29 7.65 7.01 6.37 5.73 5.09 4.45 3.81 3.17 2.53 1.89 1.25
Bin
0 10 20 30 40 50
Week

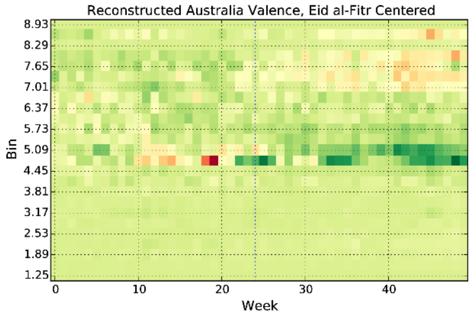
Reconstructed Australia Valence, Eid al-Fitr Centered
8.93 8.29 7.65 7.01 6.37 5.73 5.09 4.45 3.81 3.17 2.53 1.89 1.25
Bin
0 10 20 30 40
Week

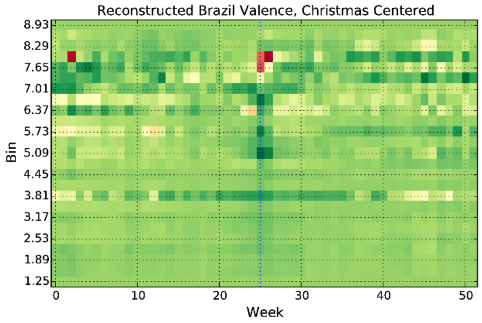
Reconstructed Brazil Valence, Christmas Centered
8.93 8.29 7.65 7.01 6.37 5.73 5.09 4.45 3.81 3.17 2.53 1.89 1.25
Bin
0 10 20 30 40 50
Week

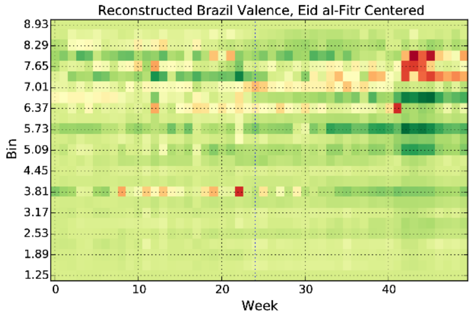
Reconstructed Brazil Valence, Eid al-Fitr Centered
8.93 8.29 7.65 7.01 6.37 5.73 5.09 4.45 3.81 3.17 2.53 1.89 1.25
Bin
0 10 20 30 40
Week

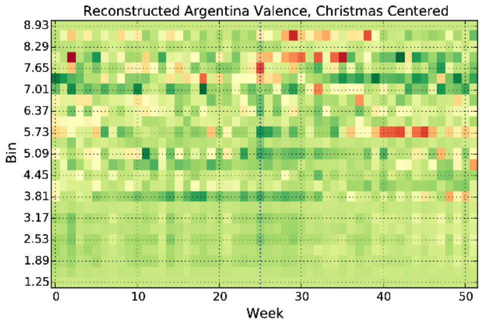
Reconstructed Argentina Valence, Christmas Centered
8.93 8.29 7.65 7.01 6.37 5.73 5.09 4.45 3.81 3.17 2.53 1.89 1.25
Bin
0 10 20 30 40 50
Week

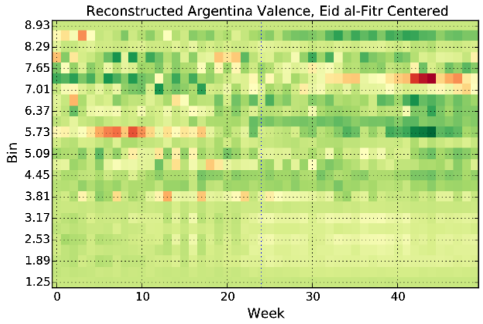
Reconstructed Argentina Valence, Eid al-Fitr Centered
8.93 8.29 7.65 7.01 6.37 5.73 5.09 4.45 3.81 3.17 2.53 1.89 1.25
Bin
0 10 20 30 40
Week

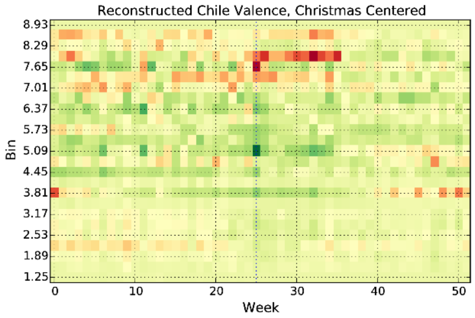
Reconstructed Chile Valence, Christmas Centered
8.93 8.29 7.65 7.01 6.37 5.73 5.09 4.45 3.81 3.17 2.53 1.89 1.25
Bin
0 10 20 30 40 50
Week

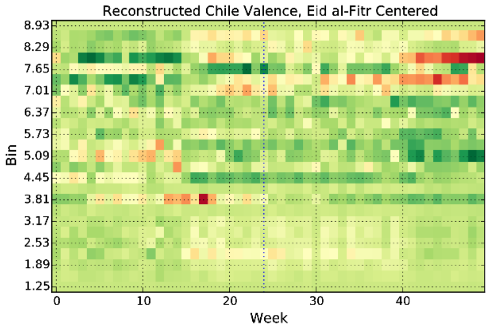
Reconstructed Chile Valence, Eid al-Fitr Centered
8.93 8.29 7.65 7.01 6.37 5.73 5.09 4.45 3.81 3.17 2.53 1.89 1.25
Bin
0 10 20 30 40
Week

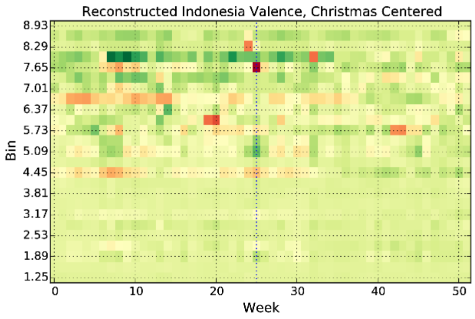
Reconstructed Indonesia Valence, Christmas Centered
Bin
Week
8.93
8.29
7.65
7.01
6.37
5.73
5.09
4.45
3.81
3.17
2.53
1.89
1.25
0
10
20
30
40
50

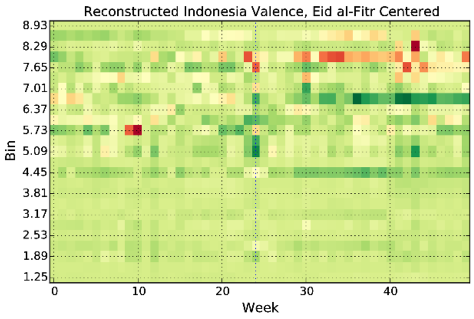
Reconstructed Indonesia Valence, Eid al-Fitr Centered
Bin
Week
8.93
8.29
7.65
7.01
6.37
5.73
5.09
4.45
3.81
3.17
2.53
1.89
1.25
0
10
20
30
40

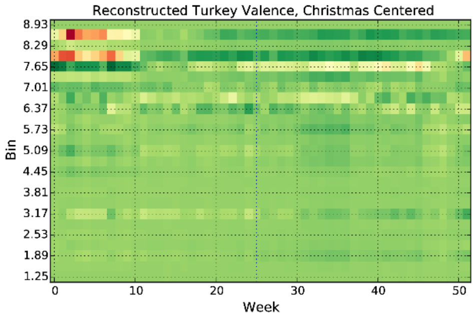
Reconstructed Turkey Valence, Christmas Centered
Bin
Week
8.93
8.29
7.65
7.01
6.37
5.73
5.09
4.45
3.81
3.17
2.53
1.89
1.25
0
10
20
30
40
50

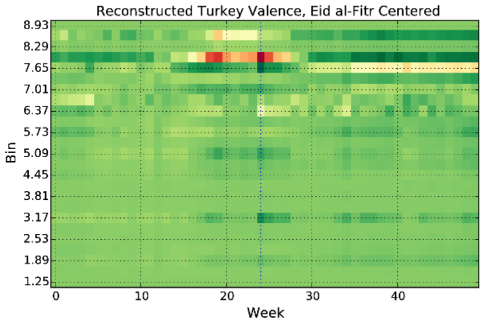
Reconstructed Turkey Valence, Eid al-Fitr Centered
Bin
Week
8.93
8.29
7.65
7.01
6.37
5.73
5.09
4.45
3.81
3.17
2.53
1.89
1.25
0
10
20
30
40

Fig. S15. Eigenmood projections and regressions. Projections show all yearly data points, projected into the space formed by the selected eigenweeks; regressions show the average year's sex searches and similarity to the holiday center.

USA Christmas

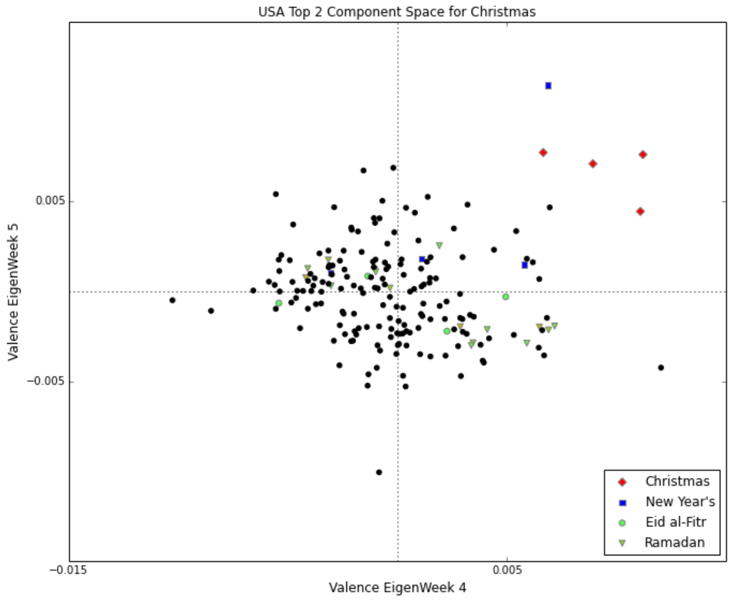

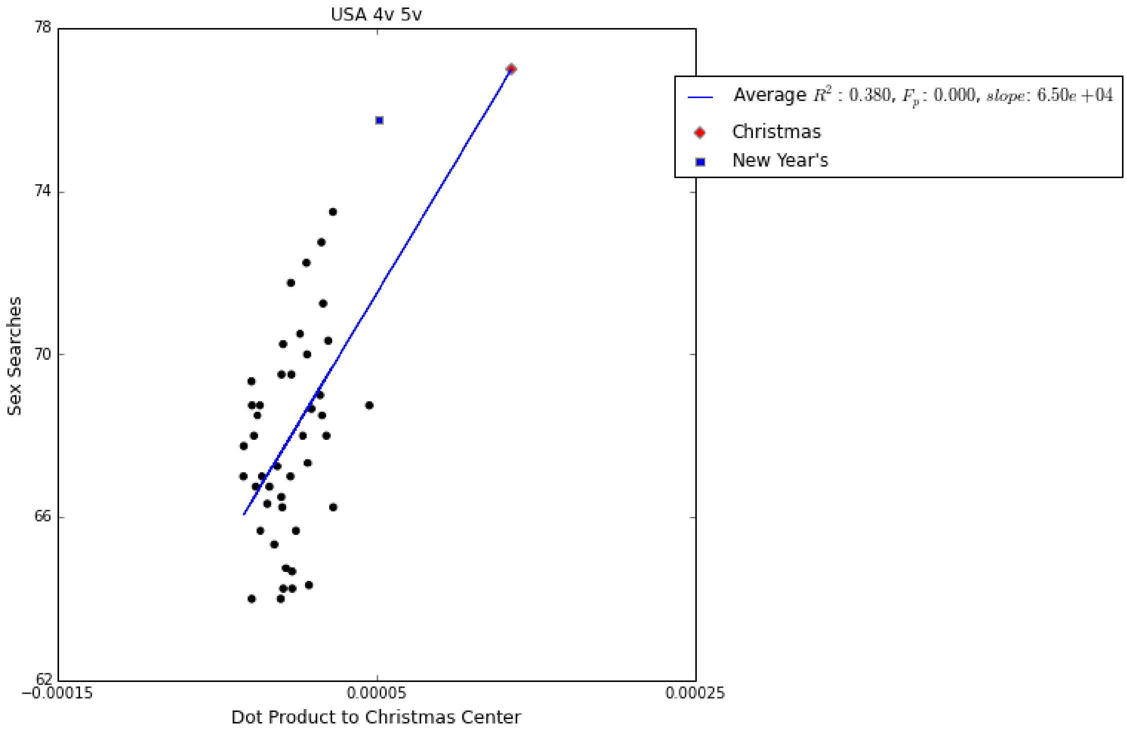

USA Eid-al-Fitr

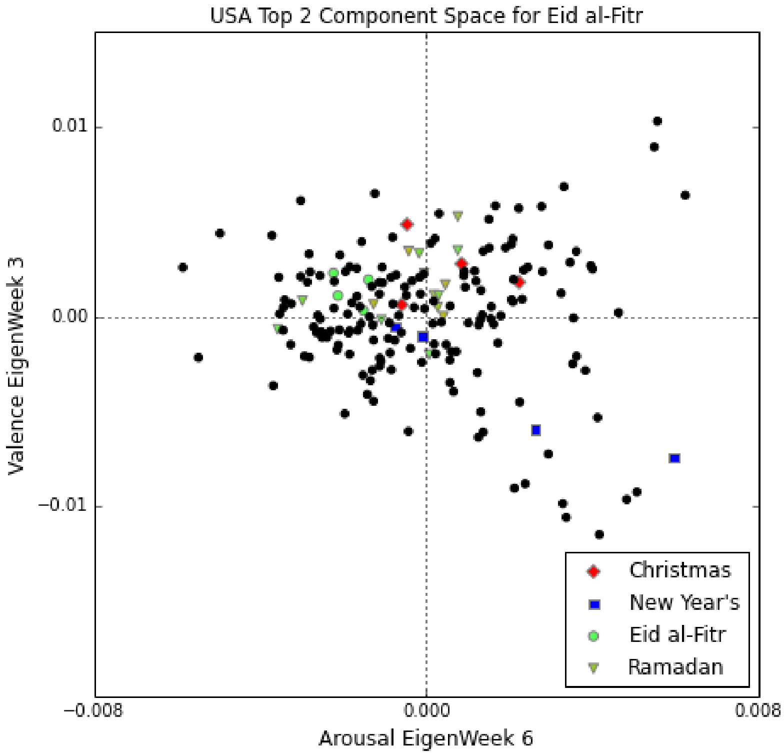

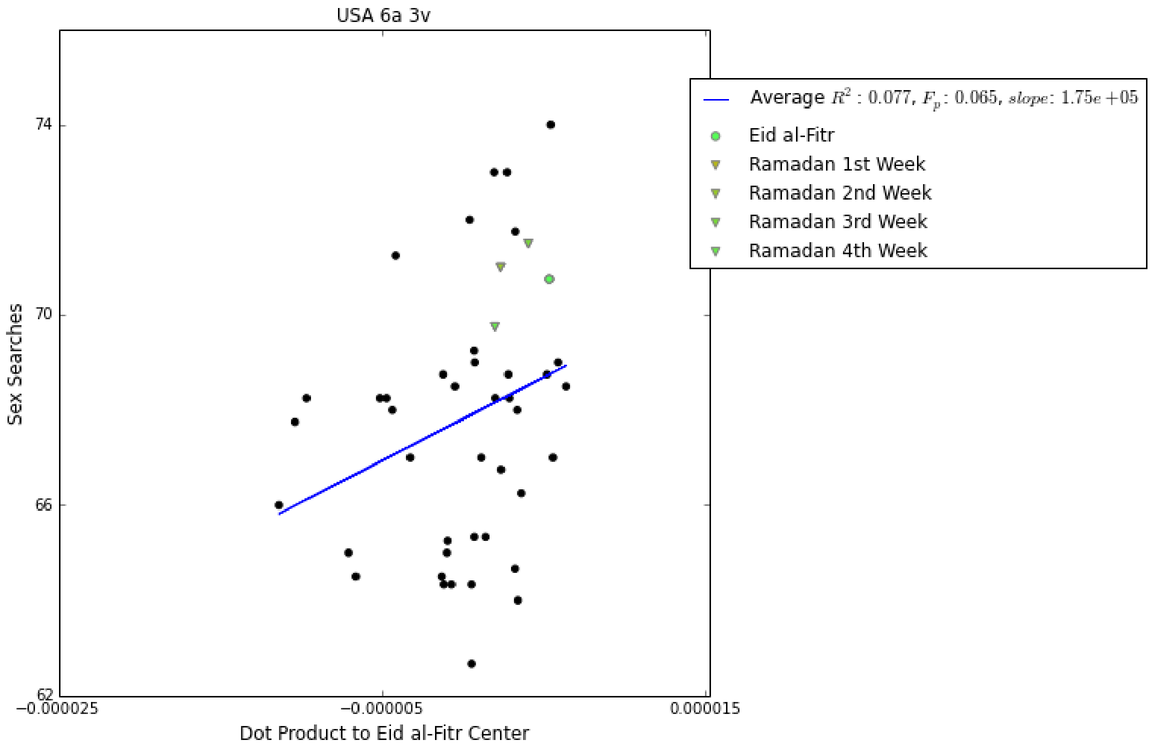

Australia Christmas

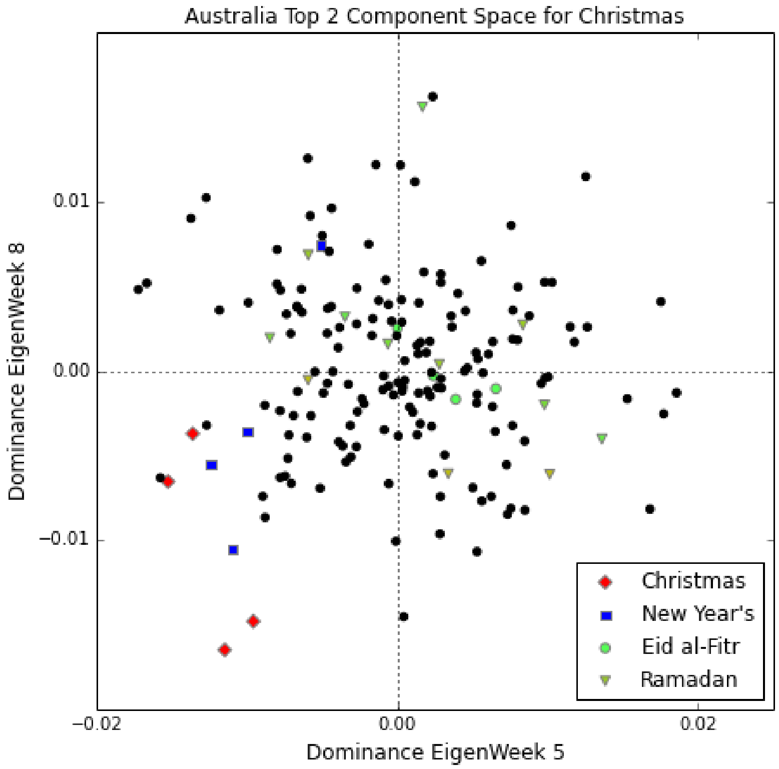

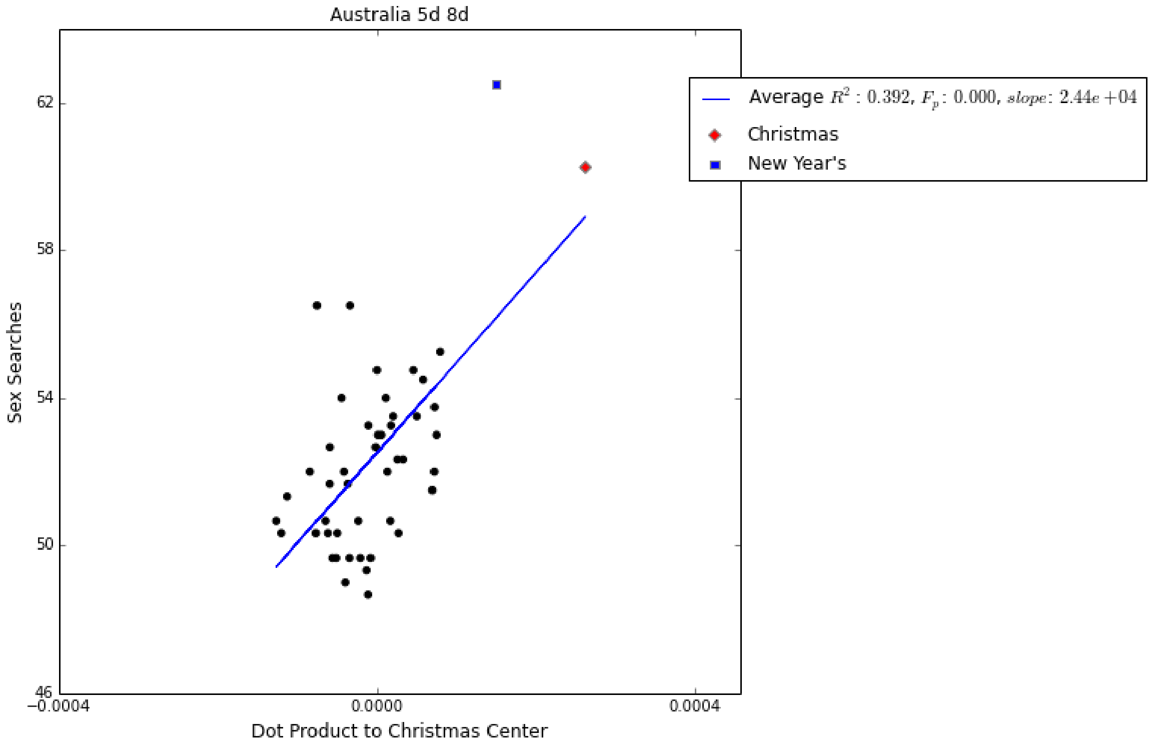

Australia Eid-al-Fitr

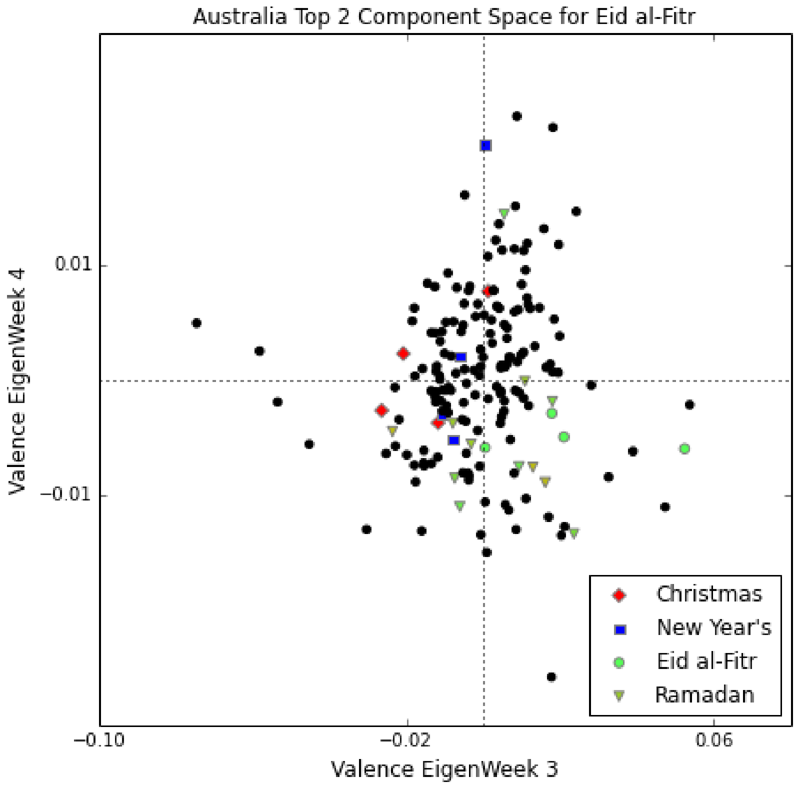

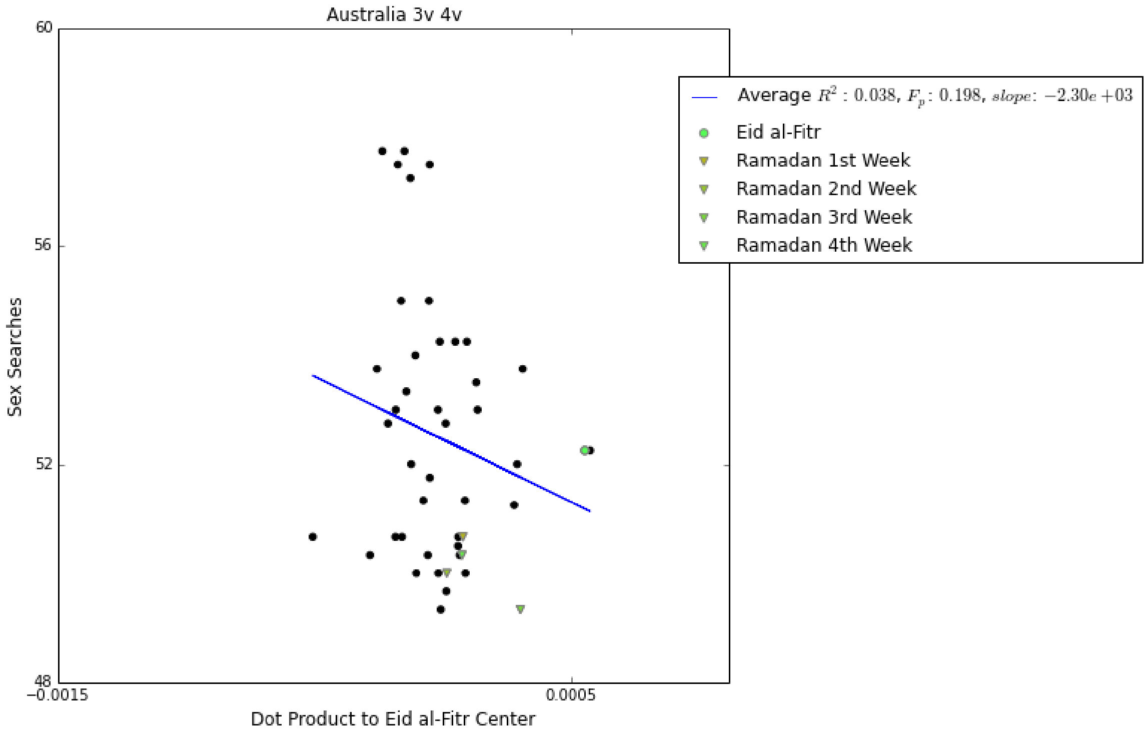

Brazil Christmas

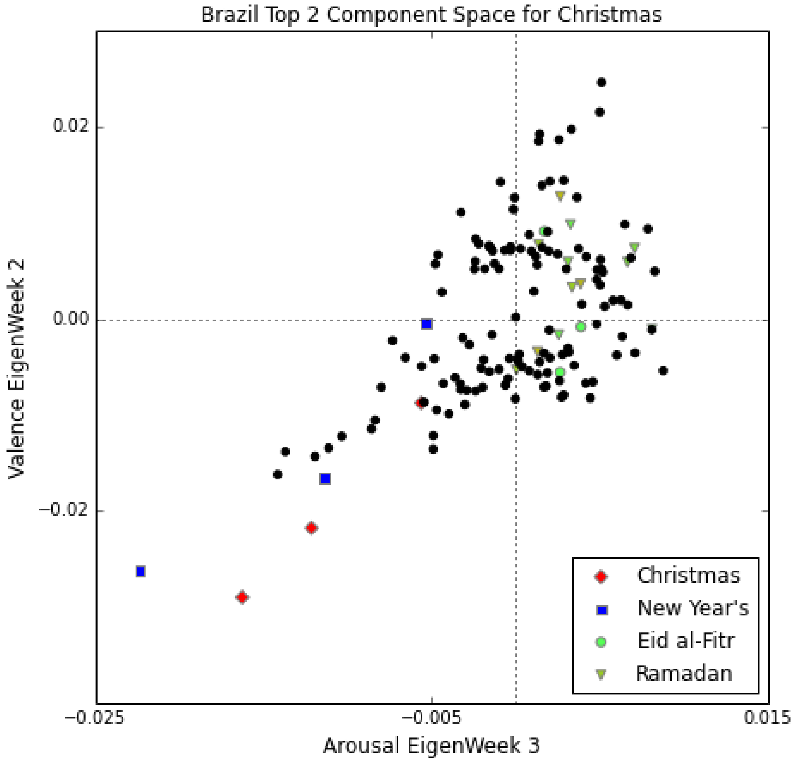

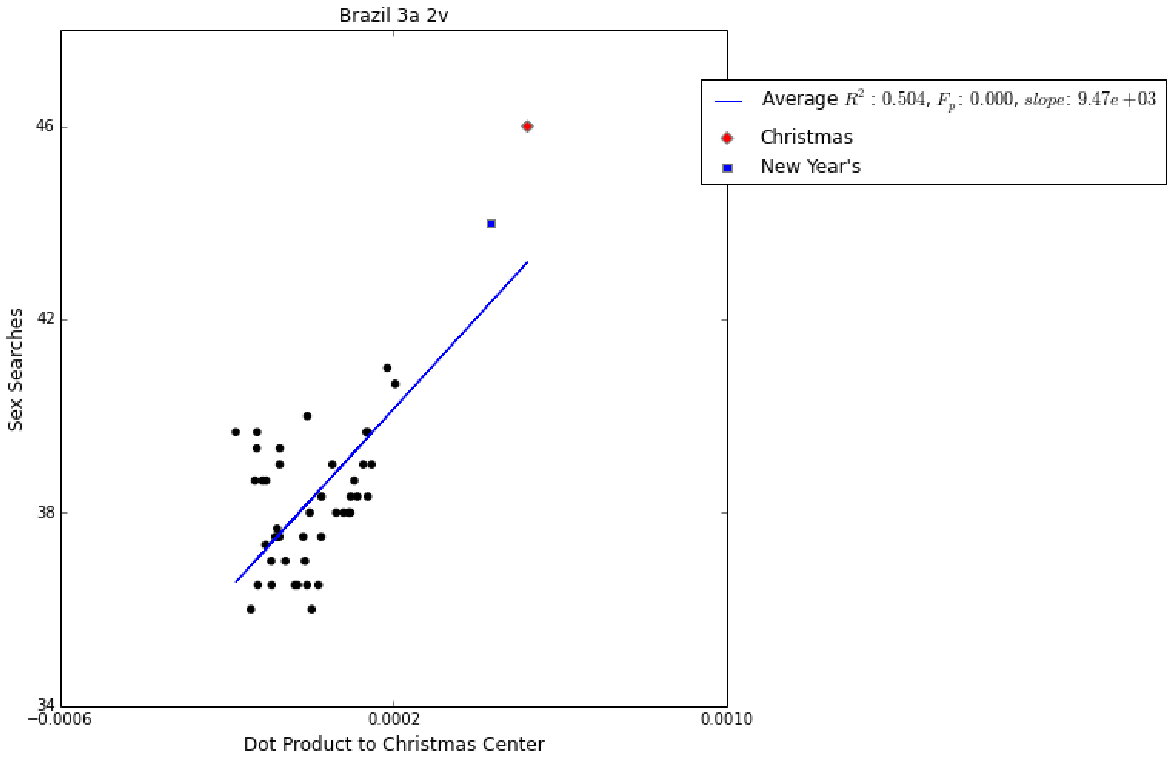

Brazil Eid-al-Fitr

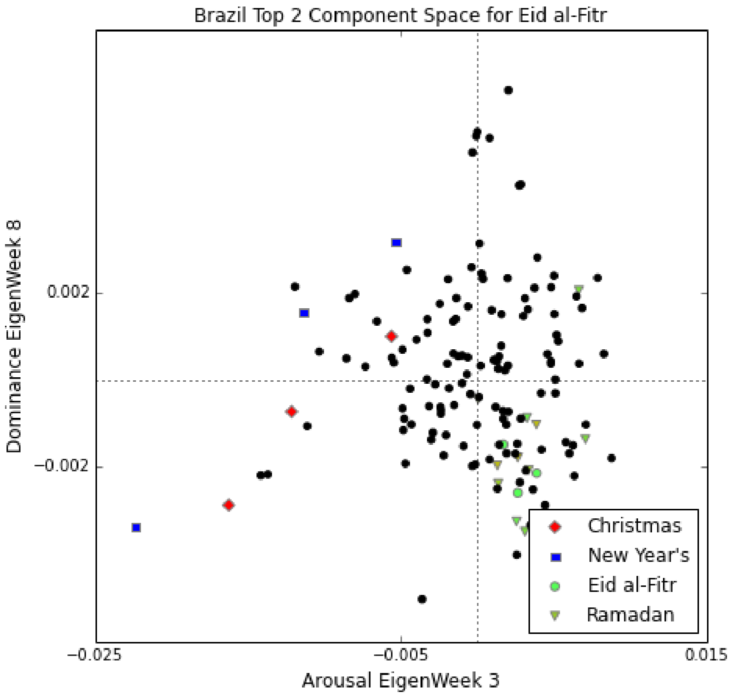

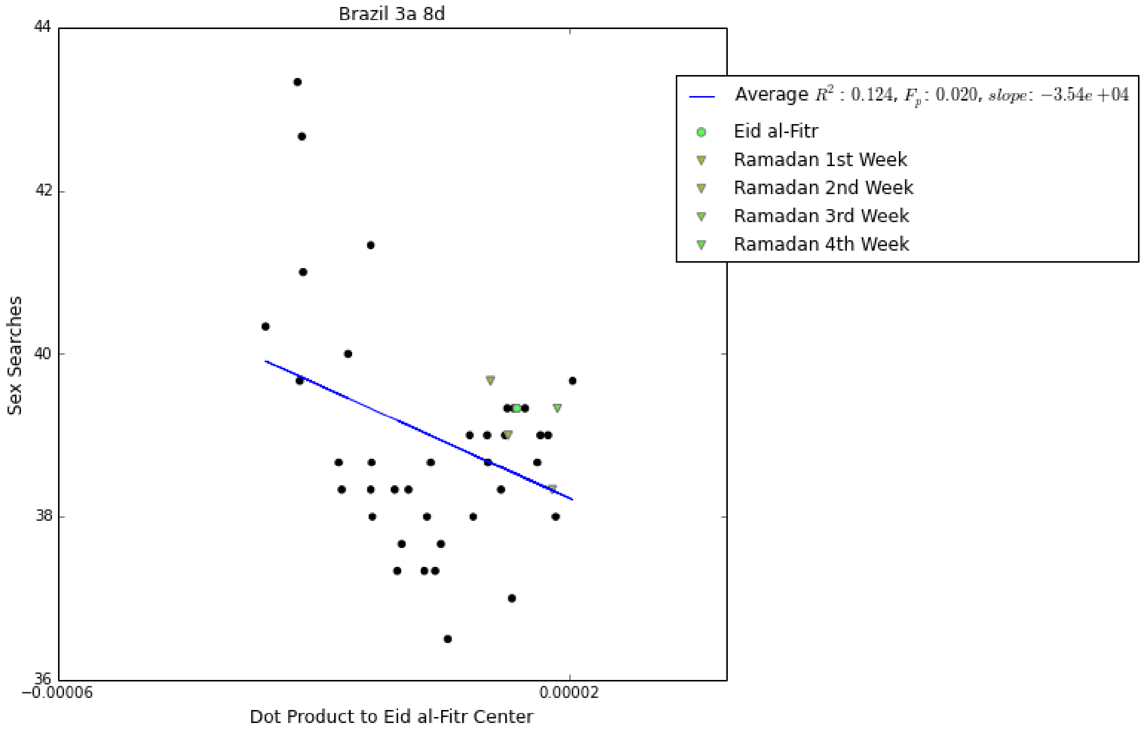

Argentina Christmas

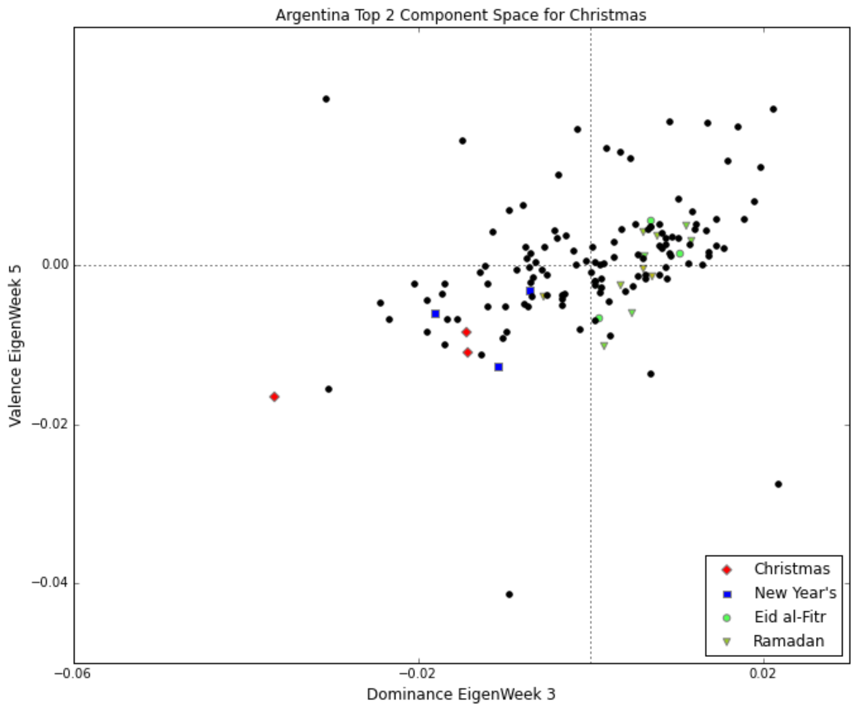

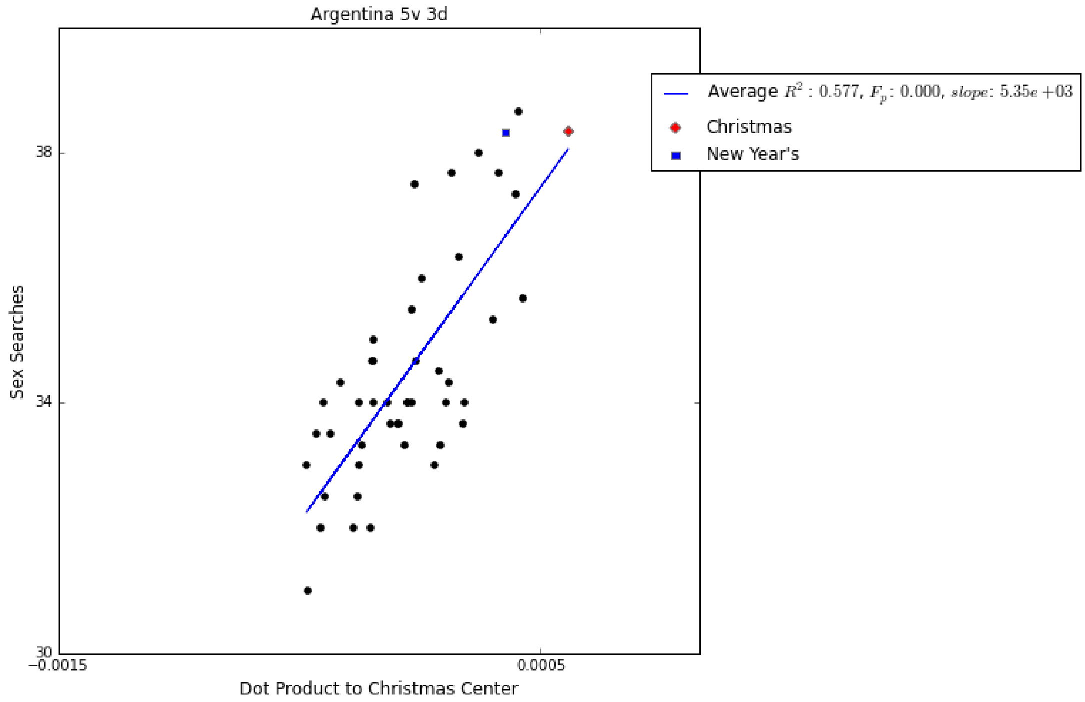

Argentina Eid-al-Fitr

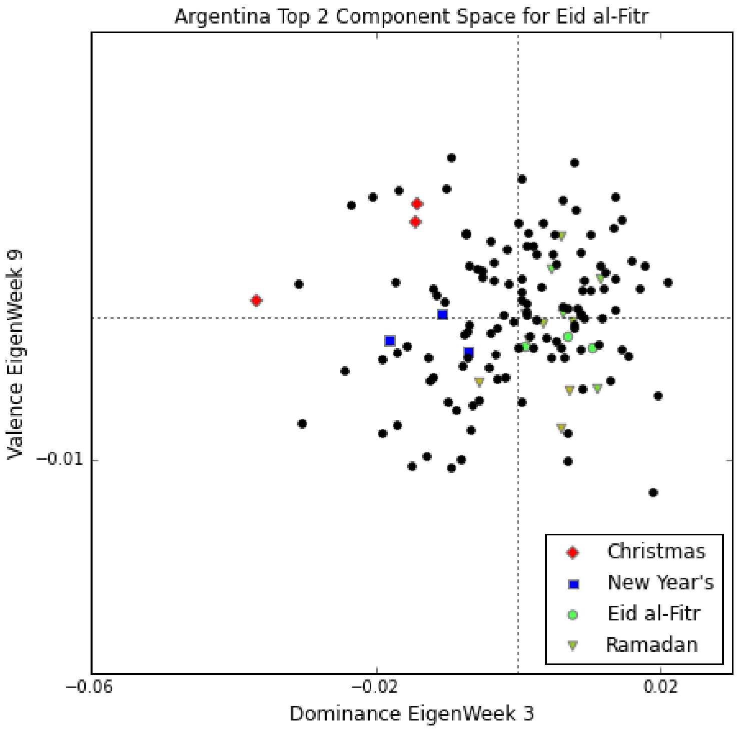

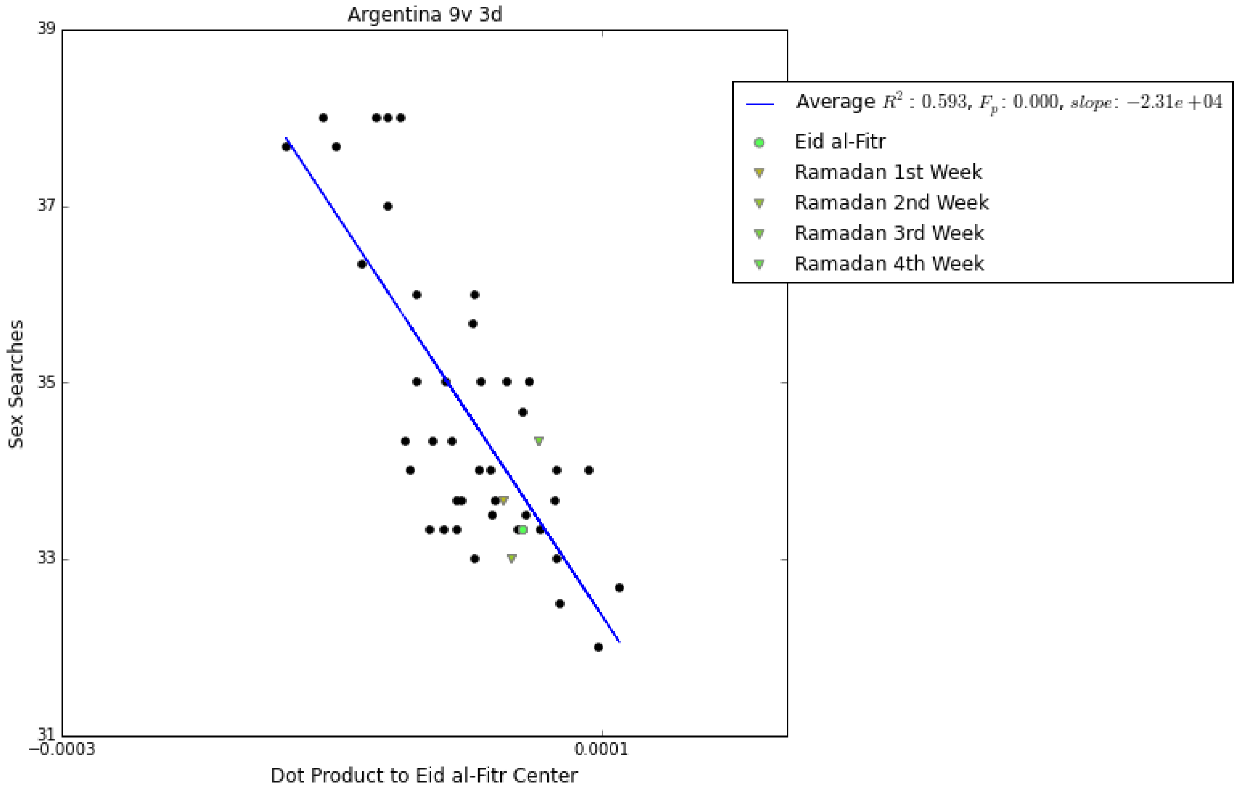

Chile Christmas

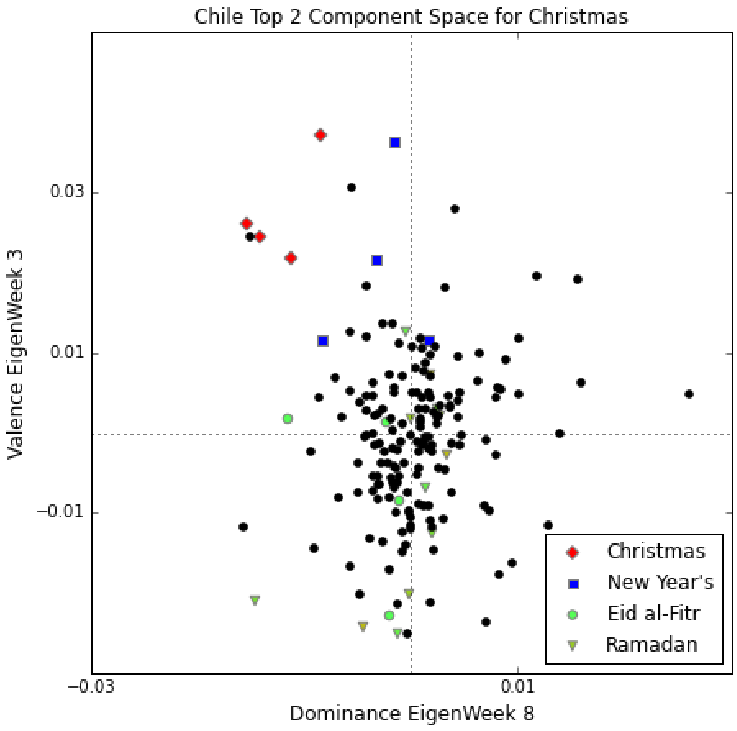

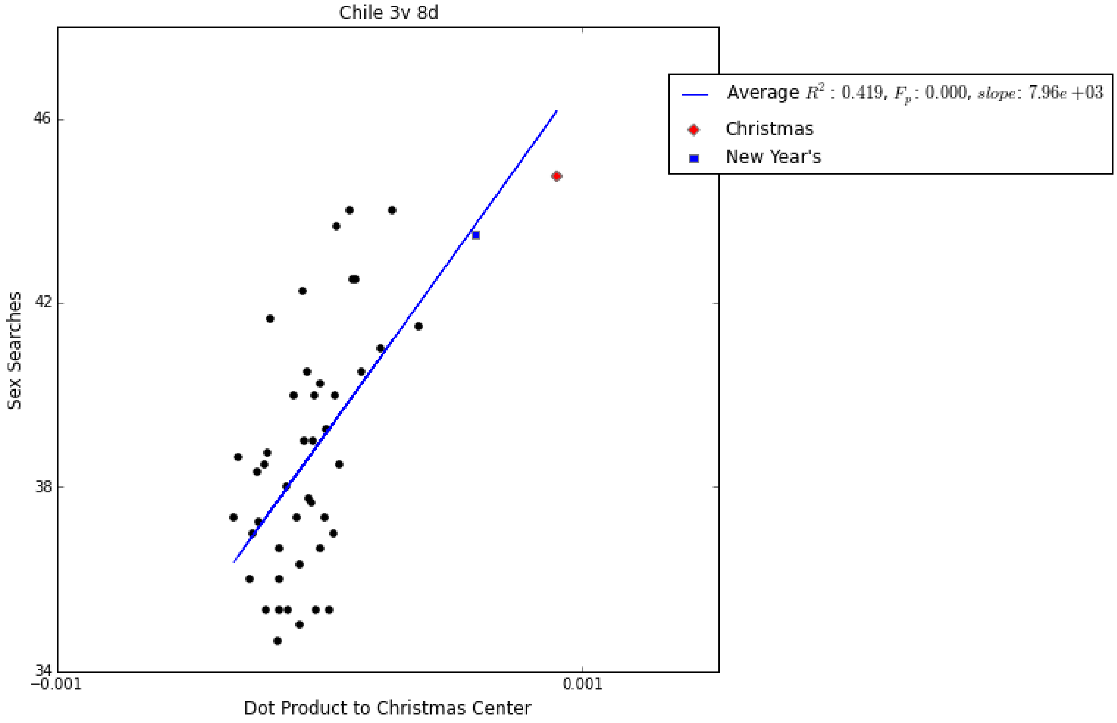

Chile Eid-al-Fitr

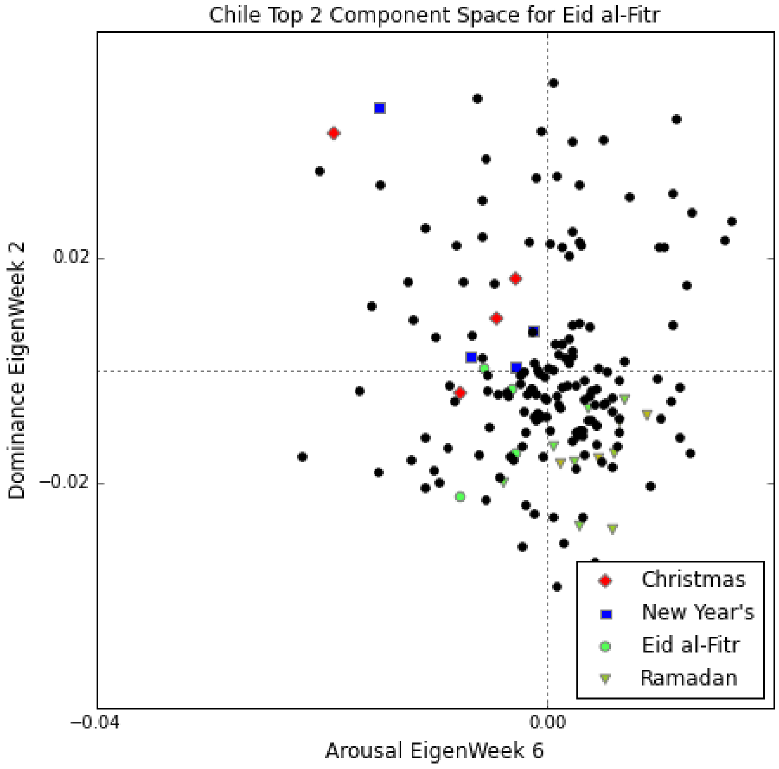

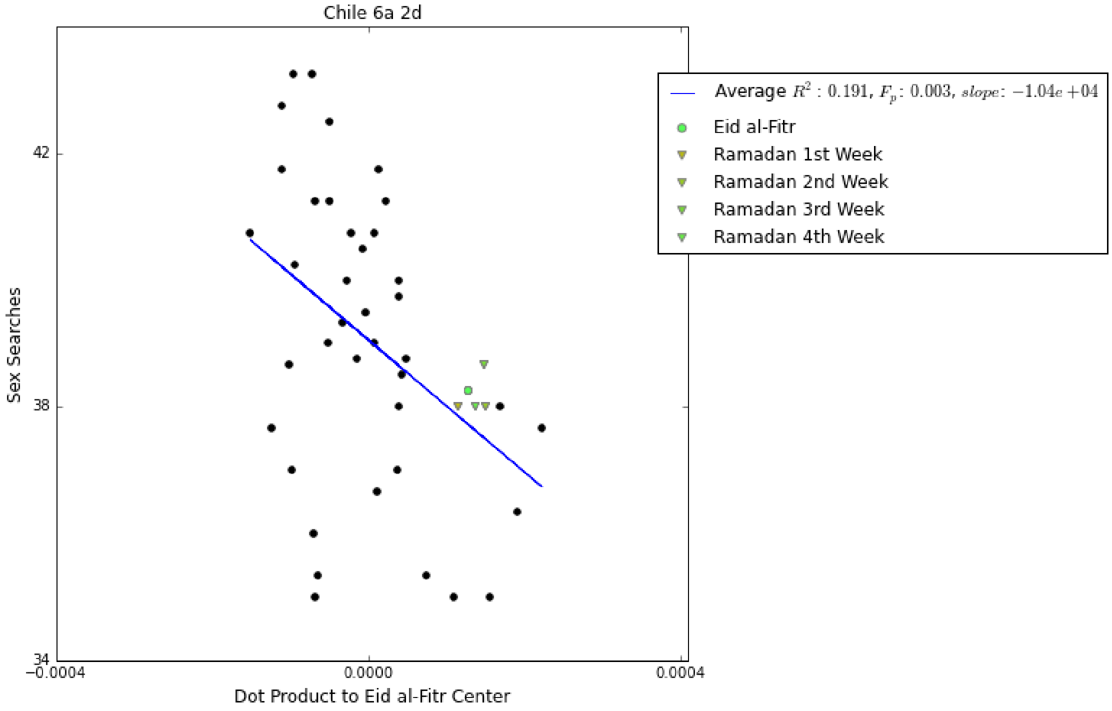

Indonesia Christmas

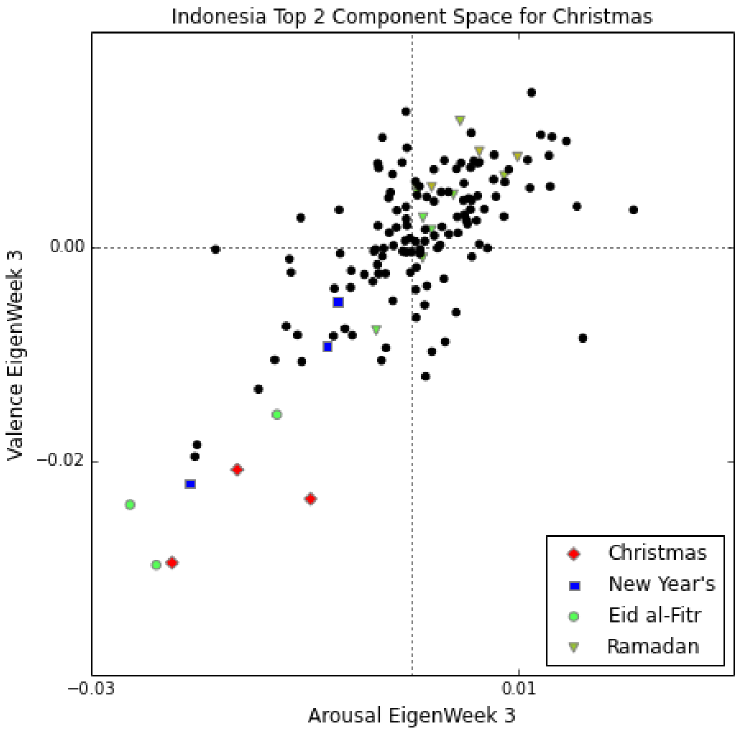

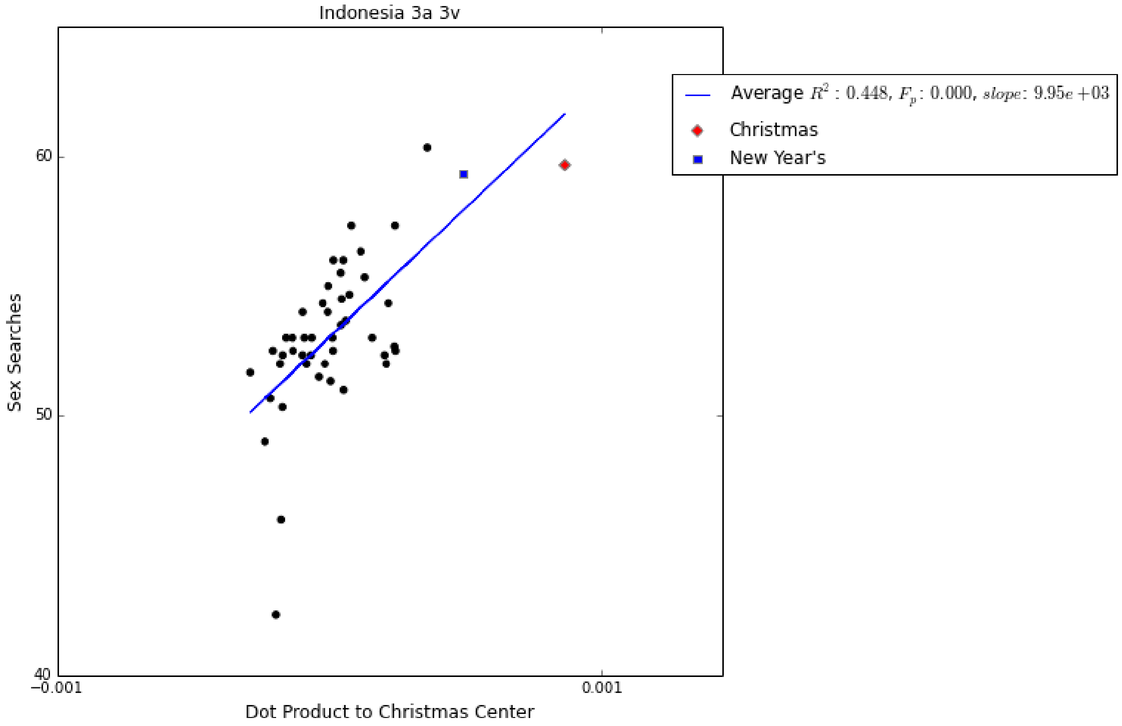

Indonesia Eid-al-Fitr

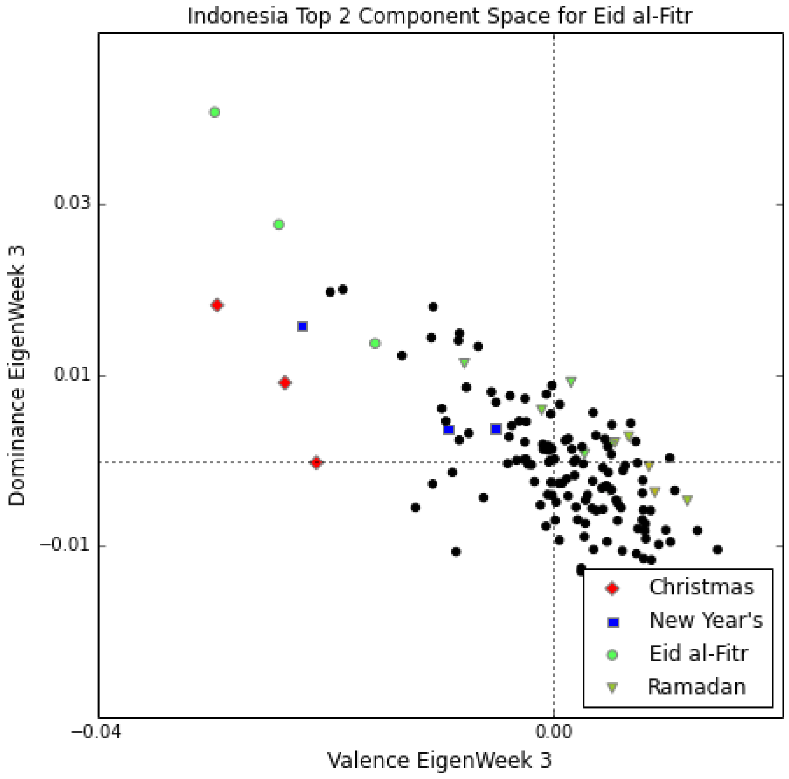

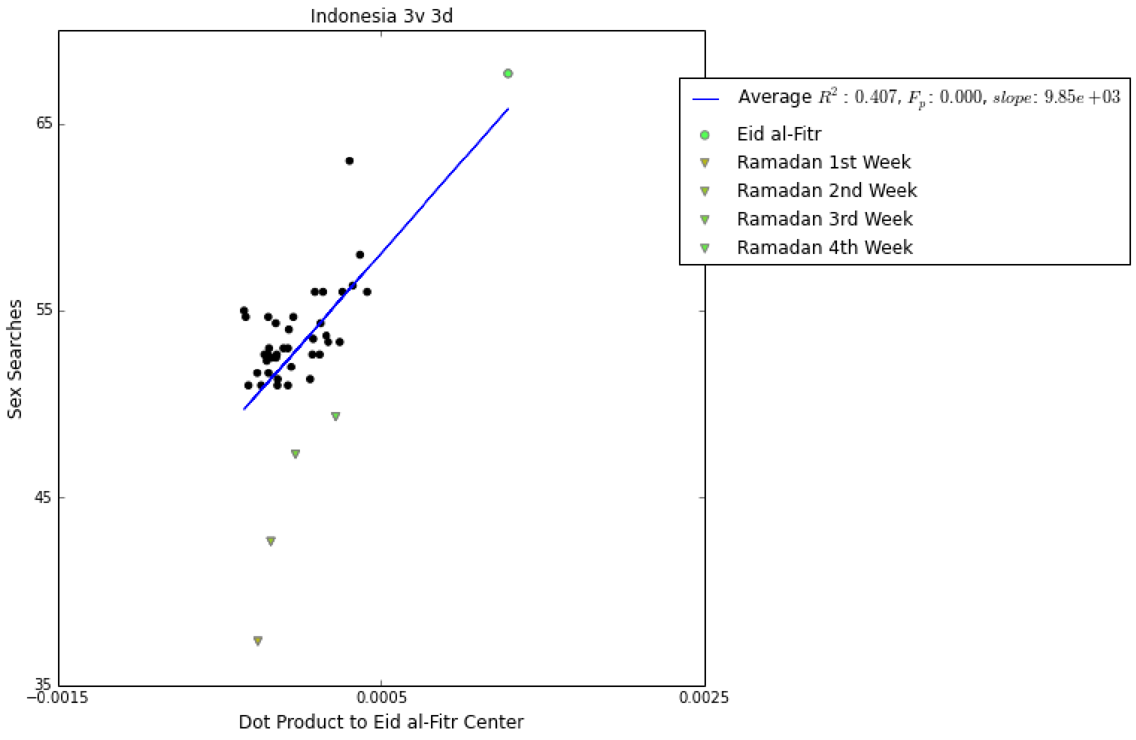

Turkey Christmas

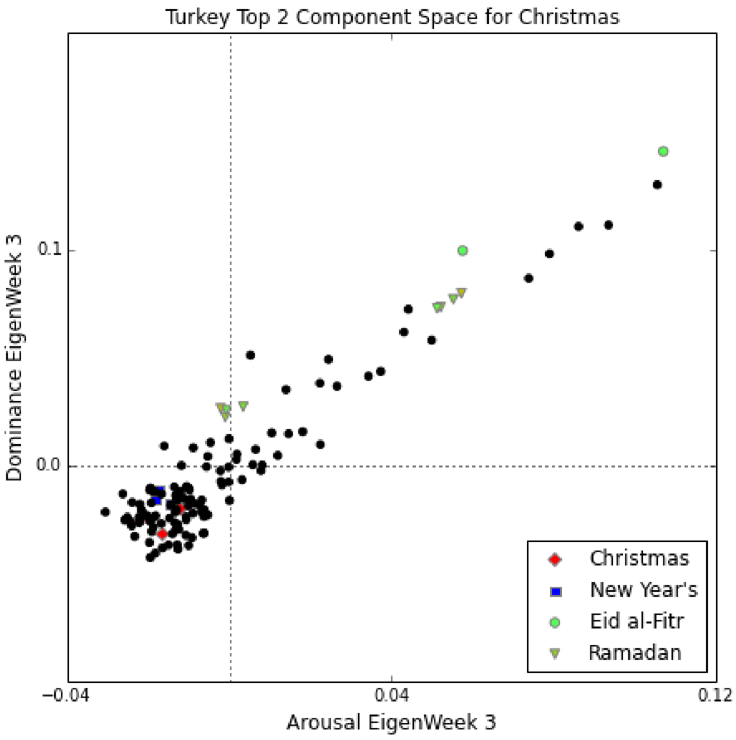

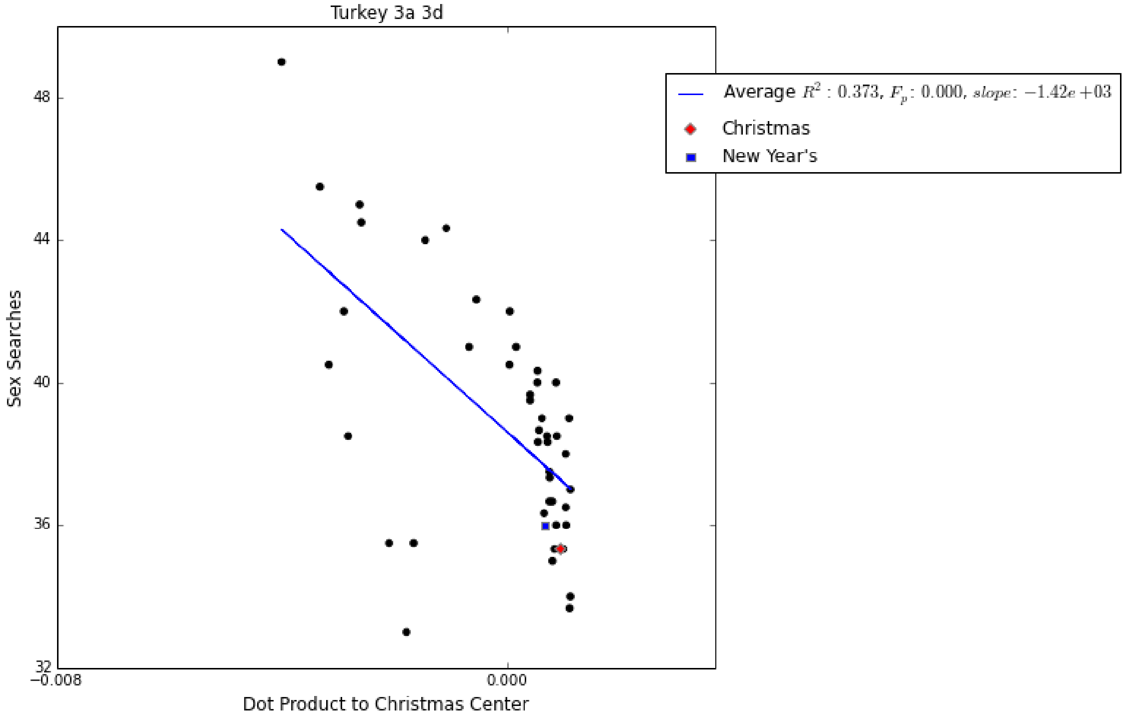

Turkey Eid-al-Fitr

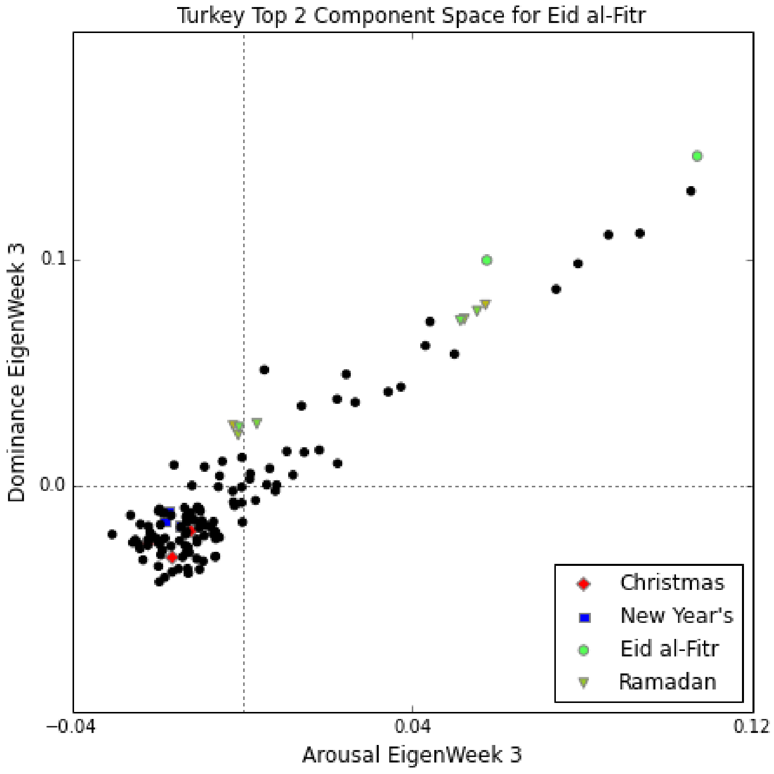

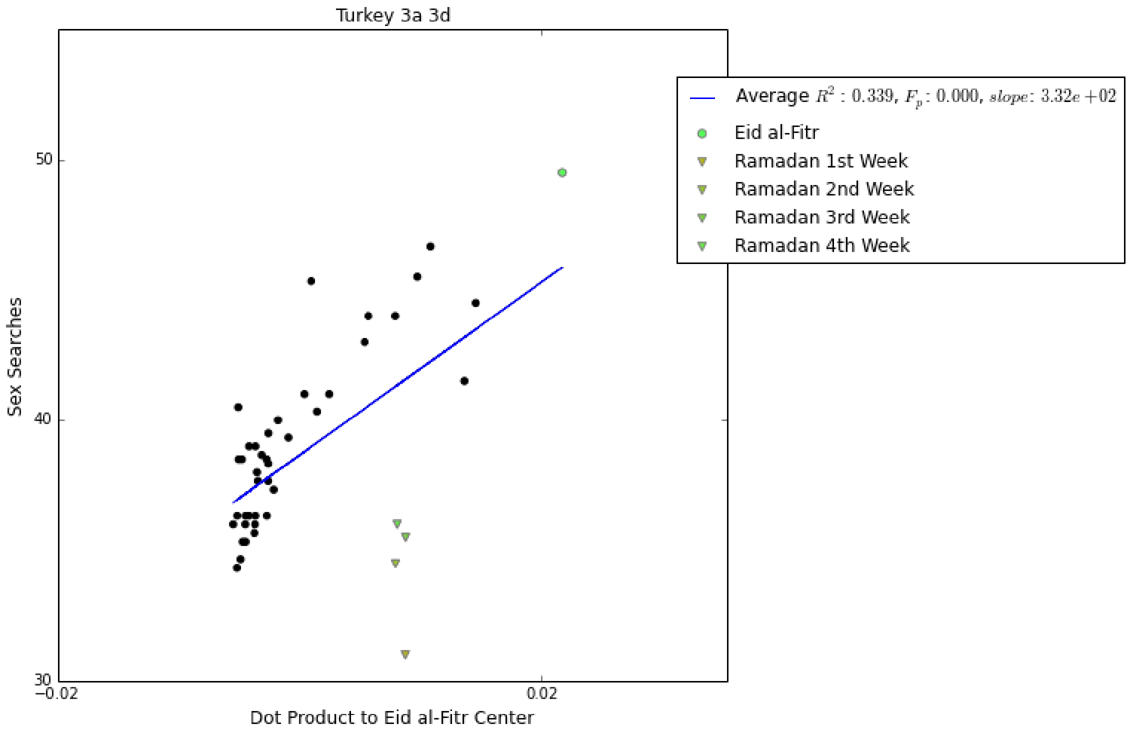

Supplementary Tables

Table S1. Searches for “sex” in select countries. Search queries for “sex” are issued in select countries, representing sexual interest in different cultures, hemispheres, and languages. Google Trends ™ allows the retrieval of search volume time series for multiple search terms. We downloaded GT data for 2 search queries: (1) for the term “sex” and (2) for its translation in the local language as detailed in Supplementary Methods. Table S1 shows the 25 countries and languages that retrieved a sufficiently significant search volume in the local language to support our analysis. From left to right, columns show the: “Countries” for which the analysis was performed; “Search term” in GT; the “Top 5 words associated with the search term”, provided and ranked by Google Trends; the “Search Volume Ratio”, calculated as the number of searches for “sex” divided by the number of searches for the corresponding translation; and the “Correlation between the two time series (“sex” and the translated word).
The English word ”sex” is either more searched for than the corresponding word in the local language (blue to red in the 4th column) or there is a strong correlation between the search terms (red in the 5th column). This is consistent with the fact that the top 5 broad searches most associated with “sex” returned by GT refer to interest in sexual content and pornography in every country (3rd column) and that sexual materials and pornography are widely available in English. The two exceptions are Russia and Israel and neither of these countries is relevant to our analysis.

Country	Search Term	Top 5 Words associated with search term	Search volume ratio	Correlation
Argentina	sex	sex free, sex videos, porn sex, porn, video sex		
	sexo	videos, **sexo** videos, sexo gratis, videos de sexo, porno	0.27	0.81
Australia	sex	free sex, porn sex, porn, sex stories, sex videos		
Brazil	sex	videos sex, sexo, videos, sex video, sex shop		
	sexo	videos sexo, videos, videos de sexo, sexo video, sexo gratis	0.17	0.71
Bulgaria	sex	porno, sex porno, sex free, sex video, sex bg		
	секс	**секс** порно (sex porn), порно (porn), секс игри (sex game), секс клипове (sex videos), клипове (videos)	5.11	-0.08
Chile	sex	sex free, sex videos, video sex, sex porn, sexo		
	sexo	videos, **sexo** videos, sexo gratis, videos de sexo, porno	0.23	0.81
Egypt	sex	sex free, sex arab, sex tube, sex movies, hot sex		
	جنس	سكس (sex), سكس جنس (sex sex), افلام جنس (sex movies), افلام (films), قصص جنس (sex stories)	8.04	0.83
	سكس	سكس سكس (sex sex), افلام سكس (sex videos), افلام (films), صور (picture), صور سكس (photo)	1.15	-0.56
France	sex	sex video, sex free, free, porn sex, porn		
	sexe	video **sexe,** video, sexe gratuit, sexe amateur, amateur	0.98	0.82
Germany	sex	sex free, video sex, sex videos, porn, sex porn		
Greece	sex	sex free, video sex, sex videos, porn, sex porn		
	σεξ	**σεξ** βιντεο (sex video), βιντεο (video), σεξ πορνο (sex porn), πορνο (porn), ιστοριες σεξ (sex stories)	15.60	0.43
India	sex	indian sex, sex videos, free sex, sex stories, hot sex		
Indonesia	sex	cerita sex, cerita, video sex, video, foto sex		
	seks	cerita (story), cerita **seks,** video seks, video, foto seks	7.18	0.20
Israel	sex	sex free, , sex video, sex porn, porn		
	סקס	סקס (sex), סרטי סקס (sex videos), סקס חינם (free sex), , , סרטי סקס חינם (free sex movies)	0.72	0.24
Italy	sex	video, sex video, free sex, porno, porno sex		
	sesso	video **sesso,** video, porno, sesso porno, sesso gratis	1.22	0.54
Japan	sex	sex xxx, xxx, sex, sex, sex		
	セック	セックス動画 (sex video), セックス画像 (sex image), エロ (hello), 無料セックス (free sex)	1.70	-0.30
New Zealand	sex	sex free, porn, sex porn, sex stories, sex videos		
Portugal	sex	free sex, videos sex, porn sex, porn, sex video		
	sexo	videos sexo, videos, videos de sexo, sexo gratis, sexo filmes	0.52	0.85
Russia	sex	sex video, sex free, porno, porno sex, porn sex		
	секс	порно секс (porno sex), порно (porn), видео секс (sex video), фото секс (photo sex), онлайн секс (online sex)	0.61	-0.48
South Africa	sex	porn sex, porn, free sex, sex videos, sex pics		
Spain	sex	sex free, videos, videos sex, sex porn, porn		
	sexo	sexo gratis, videos, videos sexo, videos de sexo, porno	0.46	0.68
Sweden	sex	free sex, porn, sex porn, sex video, sex tube		
Tunisia	sex	sex sex, porno sex, video sex, video, porno		
	جنس	**جنس** عربي (arabic sex), افلام جنس (sex movies), افلام (films), سكس (sex), قصص جنس (sex stories)	32.74	0.62
	سكس	**سكس** سكس (sex), سكس عربي (sex arabic), افلام (films), افلام سكس (sex videos), قصص سكس (sex stories)	19.32	0.71
Turkey	sex	porno sex, porno, sex izle, sex hikayeleri, porn		
	seks	porno **seks,** porno, seks hikayeleri (sex stories), seks izle (watch sex), sex	3.29	0.92
UK	sex	free sex, sex porn, porn, sex videos, sex tape		
USA	sex	free sex, sex videos, sex porn, porn, video sex		
Vietnam	sex	phim (movies), phim sex (porn movies), truyen sex (manga sex), truyen (manga), anh sex (he sex)		
Worldwide	sex	sex free, free, sex videos, sex porn, porn		

Table S2. Countries analyzed and categorized according to religion and geographical location (hemisphere) The 1st column shows the international country code, the 2nd columns shows the Country name; the third column (Week) shows the first week for which we could find stable GT™ data. A country was considered "culturally Christian" when at least half of its population identified as Christian (Catholic, Protestant, Orthodox, or other) according to [13]. A country was considered "culturally Muslim" when at least half of its population identified as Muslim according to [14]. A country was labeled as "Other" when the majority of its population didn't identify as either Christian or Muslim. The 4th column, "Country Set" shows how each country was categorized and the 5th and 6th columns show the percentage of the population that identify as Christian or Muslim, respectively. The 7th and 8th columns show the continent and the Hemisphere to which each country belongs, according to Wikipedia.

Code	Country Name	First Week	Country Set	% Christian	% Muslim	Continent	Hemisphere
AE	United Arab Emirates	04-01-2004	Muslim	2.6 (2.6;)	76	Asia	North
AF	Afghanistan	12-11-2006	Muslim	0.02 (;)	99.8	Asia	North
AL	Albania	06-11-2005	Muslim	17 (7;10)	82.1	Europe	North
AR	Argentina	04-01-2004	Christian	90 (77;13)	2.5	South America	South
AT	Austria	04-01-2004	Christian	68.4 (62.4;6)	5.7	Europe	North
AU	Australia	04-01-2004	Christian	63 (25.8;37)	1.9	Oceania	South
AW	Aruba	04-06-2006	Christian	88 (80.8;7.8)	0	North America	South
BA	Bosnia and Herzegovina	04-01-2004	Christian	52 (15;37)	41.6	Europe	North
BD	Bangladesh	04-01-2004	Muslim	0.3 (0.3;)	90.4	Asia	North
BE	Belgium	04-01-2004	Christian	55.4 (57;7)	6	Europe	North
BG	Bulgaria	04-01-2004	Christian	84 (1;83)	13.4	Europe	North
BH	Bahrain	04-01-2004	Muslim	9 (;9)	81.2	Asia	North
BN	Brunei	08-01-2006	Muslim	11 (;)	51.9	Asia	North
BO	Bolivia	04-01-2004	Christian	89 (76;13)	2.5	South America	South
BR	Brazil	04-01-2004	Christian	90.2 (63;27)	0.1	South America	South
BS	Bahamas	05-06-2005	Christian	81 (13.5;67.6)	0	Central America	North
BY	Belarus	01-01-2006	Christian	55.4 (7.1;48.3)	0.2	Europe	North
CA	Canada	04-01-2004	Christian	67.3 (38.7;29)	2.8	North America	North
CH	Switzerland	04-01-2004	Christian	71 (38;33)	5.7	Europe	North
CL	Chile	04-01-2004	Christian	87.2 (67;20)	0	South America	South
CM	Cameroon	26-08-2007	Christian	65 (38.4;26.3)	18	Africa	North
CN	China	04-01-2004	Other	5 (1;4)	1.8	Asia	North
CO	Colombia	04-01-2004	Christian	90 (75;15)	0	South America	North
CR	Costa Rica	04-01-2004	Christian	83 (69;14)	0	Central America	North
CY	Cyprus	04-01-2004	Christian	79.3 (4.3;75)	22.7	Europe	North
CZ	Czech Republic	04-01-2004	Other	11.2 (10.4;0.8)	0	Europe	North
DE	Germany	04-01-2004	Christian	62 (30;32)	5	Europe	North
DJ	Djibouti	06-01-2008	Muslim	6 (1;5)	97	Africa	North
DK	Denmark	04-01-2004	Christian	81 (1;80)	4.1	Europe	North

Code	Country Name	First Week	Country Set	% Christian	% Muslim	Continent	Hemisphere
DO	Dominican Republic	04-01-2004	Christian	95 (95;)		North America	North
DZ	Algeria	04-01-2004	Muslim	2 (1;1)	98.2	Africa	North
EC	Ecuador	04-01-2004	Christian	94 (74;20)	0	South America	South
EE	Estonia	04-01-2004	Other	23.9 (0;23)	0.1	Europe	North
EG	Egypt	04-01-2004	Muslim	18 (0;18)	94.7	Africa	North
ES	Spain	04-01-2004	Christian	73 (71;2)	2.3	Europe	North
ET	Ethiopia	04-01-2004	Christian	63.4 (0;63.4)	33.8	Africa	North
FI	Finland	04-01-2004	Christian	81.6 (0;81)	0.8	Europe	North
FJ	Fiji	03-09-2006	Christian	64.4 (8.9;55.5)	6.3	Oceania	South
FR	France	04-01-2004	Christian	65 (63;2)	7.5	Europe	North
GE	Georgia	01-05-2005	Christian	88.6 (0.9;87.7)	10.5	Europe	North
GH	Ghana	16-10-2005	Christian	68.8 (13.1;55.5)	16.1	Africa	North
GP	Guadalupe	09-03-2008	Christian	96 (95;1)		North America	North
GR	Greece	04-01-2004	Christian	97 (0;97)	4.7	Europe	North
GT	Guatemala	04-01-2004	Christian	87 (47;40)	0	Central America	North
GU	Guam	17-12-2006	Christian	85 (;)	0.1	Oceania	South
HN	Honduras	04-09-2005	Christian	87.6 (47;40)	0.1	Central America	North
HR	Croatia	04-01-2004	Christian	90(70;20)	1.3	Europe	North
HU	Hungary	04-01-2004	Christian	82.7 (70.1;11.6)	0.3	Europe	North
ID	Indonesia	04-01-2004	Muslim	10(3;7)	88.1	Asia	South
IE	Ireland	04-01-2004	Christian	94.1 (82;12)	0.9	Europe	North
IL	Israel	04-01-2004	Other	3.5(;3.5)	17.7	Asia	North
IN	India	04-01-2004	Other	2.6 (1.6;1)	14.6	Asia	North
IQ	Iraq	12-12-2004	Muslim	3(;3)	98.9	Asia	North
IR	Iran	04-01-2004	Muslim	0.4(;)	99.7	Asia	North
IS	Iceland	04-01-2004	Christian	95 (2.5;92.5)	0.1	Europe	North
IT	Italy	04-01-2004	Christian	85.1 (85;0)	2.6	Europe	North
JM	Jamaica	04-01-2004	Christian	65.3 (2;63.3)	0	Central America	North
JO	Jordan	04-01-2004	Muslim	6 (;)	98.8	Asia	North
JP	Japan	04-01-2004	Other	2 (1;1)	0.1	Asia	North
KE	Kenya	04-01-2004	Christian	85.1 (23.4;61.7)	7	Africa	North
KH	Cambodia	05-12-2004	Other	1 (0.15;0.85)	1.6	Asia	North
KR	South Korea	04-01-2004	Other	(;)	0.2	Asia	North
KW	Kuwait	04-01-2004	Muslim	15 (3.2;12.8)	86.4	Asia	North
KZ	Kazakhstan	01-10-2006	Muslim	51 (0.16;50)	56.4	Europe	North
LA	Laos	15-04-2007	Other	2.2 (1;1)	0	Asia	North

Code	Country Name	First Week	Country Set	% Christian	% Muslim	Continent	Hemisphere
LB	Lebanon	04-01-2004	Muslim	41 (26;15)	59.7	Asia	North
LK	Sri Lanka	04-01-2004	Other	7.5 (6.1;1.4)	8.5	Asia	North
LT	Lithuania	04-01-2004	Christian	84.9 (77.2;7.6)	0.1	Europe	North
LU	Luxemburg	04-01-2004	Christian	71 (69;2)	2.3	Europe	North
LV	Latvia	04-01-2004	Christian	57 (25;32.2)	0.1	Europe	North
MA	Morocco	04-01-2004	Muslim	2.1 (0.1;2)	99.9	Africa	North
MD	Moldova	02-10-2005	Christian	97.53 (0;93)	0.4	Europe	North
ME	Montenegro	13-11-2005	Christian	78.8 (3.4;72.07)	18.5	Europe	North
MK	Macedonia	04-01-2004	Christian	65.1 (0.3;64.8)	34.9	Europe	North
MM	Myanmar	04-12-2005	Other	7.9 (1;6.9)	3.8	Asia	North
MN	Mongolia	14-08-2005	Other	2.1 (;)	4.4	Asia	North
MT	Malta	04-01-2004	Christian	97 (;)	0.3	Europe	North
MU	Mauritius	10-07-2005	Other	32.2 (-;-)	16.6	Africa	South
MV	Maldives	04-01-2004	Muslim	41 (26;15)	98.4	Asia	North
MX	Mexico	04-01-2004	Christian	92 (;)	0.1	North America	North
MY	Malaysia	04-01-2004	Muslim	12.1 (;)	61.4	Asia	North
MZ	Mozambique	24-02-2008	Christian	56.1 (28.4;27.7)	22.8	Africa	South
NA	Namibia	27-06-2010	Christian	90 (13.7;76.3)	0.4	Africa	South
NG	Nigeria	04-01-2004	Christian	50.01 (14;36)	47.9	Africa	North
NI	Nicaragua	16-08-2009	Christian	89.6 (58.8;30.8)	0	Central America	North
NL	Netherlands	04-01-2004	Other	44 (24;20)	5.5	Europe	North
NO	Norway	04-01-2004	Christian	86.2 (3;83.5)	3	Europe	North
NP	Nepal	04-01-2004	Other	0.9 (0.1;0.8)	4.2	Asia	North
NZ	New Zealand	04-01-2004	Christian	55.6 (28.7;24.9)	0.9	South America	South
OM	Oman	04-01-2004	Muslim	2.5 (2.1;0.4)	87.7	Asia	North
PA	Panama	15-02-2004	Christian	92 (80;12)	0.7	Central America	North
PE	Peru	04-01-2004	Christian	96 (81;15)	0	South America	South
PH	Philippines	04-01-2004	Christian	93 (80;13)	5.1	Asia	North
PK	Pakistan	04-01-2004	Muslim	1.6 (0.8;0.8)	96.4	Asia	North
PL	Poland	04-01-2004	Christian	94.3 (86.3;8)	0.1	Europe	North
PR	Puerto Rico	04-01-2004	Christian	97 (50;47)	0	North America	North
PS	Palestine	04-01-2004	Muslim	(;)	97.5	Asia	North
PT	Portugal	04-01-2004	Christian	95.7 (81;14.7)	0.6	Europe	North
PY	Paraguay	12-02-2006	Christian	96 (88;7.9)	0	South America	South
QA	Qatar	04-01-2004	Muslim	13.8 (;)	77.5	Asia	North
RO	Romania	04-01-2004	Christian	99.5 (5.7;93.8)	0.3	Europe	North

Code	Country Name	First Week	Country Set	% Christian	% Muslim	Continent	Hemisphere
RS	Serbia	04-01-2004	Christian	93.5 (4.97;79.4)	3.7	Europe	North
RU	Russia	04-01-2004	Christian	60 (0;60)	11.7	Europe	North
SA	Saudi Arabia	04-01-2004	Muslim	5.5 (3.5;2)	97.1	Asia	North
SD	Sudan	11-01-2004	Muslim	2 (;)	71.4	Africa	North
SE	Sweden	04-01-2004	Christian	67.2 (2;65)	4.9	Europe	North
SG	Singapore	04-01-2004	Other	18 (5.7;12)	14.9	Asia	North
SI	Slovenia	04-01-2004	Christian	79.2 (57;22.2)	2.4	Europe	North
SK	Slovakia	04-01-2004	Christian	86.5 (75.2;11.3)	0.1	Europe	North
SV	El Salvador	04-01-2004	Christian	81.9 (52.6;29.3)	0	Central America	North
SY	Syria	04-01-2004	Muslim	10 (0;10)	92.8	Asia	North
TH	Thailand	04-01-2004	Other	0.7 (0.4;0.3)	5.8	Asia	North
TN	Tunisia	04-01-2004	Muslim	0.2 (;0.2)	99.8	Africa	North
TR	Turkey	04-01-2004	Muslim	0.2 (;)	98.6	Europe	North
TT	Trinidad and Tobago	04-01-2004	Christian	57.6 (21.5;33.4)	5.8	Central America	North
TW	Taiwan	04-01-2004	Other	3.9 (2.6;1.3)	0.1	Asia	North
TZ	Tanzania	04-01-2004	Christian	62 (;)	29.9	Africa	South
UA	Ukraine	04-01-2004	Christian	83.8 (5.9;76.7)	0.9	Europe	North
UG	Uganda	08-01-2006	Christian	88.6 (41.9;46.7)	12	Africa	North
UK	United Kingdom	04-01-2004	Christian	59.3 (8.9;50)	4.6	Europe	North
US	United States of America	04-01-2004	Christian	73 (22;51)	0.8	North America	North
UY	Uruguay	04-01-2004	Christian	58.4 (47;11)	0	South America	South
UZ	Uzbekistan	17-10-2004	Muslim	2.6 (2.6;)	96.5	Asia	North
VE	Venezuela	04-01-2004	Christian	87 (79;8)	9.3	South America	North
VN	Vietnam	04-01-2004	Other	8 (7;1)	0.2	Asia	North
YE	Yemen	04-01-2004	Muslim	0.0013 (0.0013;)	99	Asia	North
ZA	South Africa	04-01-2004	Christian	80 (5;75)	1.5	Africa	South
ZM	Zambia	06-05-2007	Christian	97.6 (25;72)	0.4	Africa	South
ZW	Zimbabwe	05-03-2006	Christian	85 (7;77)	0.9	Africa	South

Table S3. Correlation Table for the averaged time series of all countries grouped either by hemisphere (Northern or Southern) or by religion (Muslim or Christian).
Table S3a shows R^2 and Table S3b shows the corresponding p-values.

Table S3a.

	Northern	Southern	Christian	Muslim
Northern	1			
Southern	0.536811	1		
Christian	0.890322	0.627146	1	
Muslim	0.415906	0.309619	0.192213	1

Table S3b.

	Northern	Southern	Christian	Muslim
Northern	1			
Southern	5.89E-90	1		
Christian	1.3E-254	9.1E-115	1	
Muslim	2.07E-63	2.98E-44	3.27E-26	1

Table S4. The three major Muslim holidays, in regard to the Gregorian calendar, for the period under analysis.

Beginning of Ramadan	*Eid-al-Fitr*	*Eid al-Adha*
15 Oct 2004	14 Nov 2004	21 Jan 2005
4 Oct 2005	3 Nov 2005	10 Jan 2006
24 Sep 2006	23 Oct 2006	31 Dec 2006
13 Sep 2007	13 Oct 2007	20 Dec 2007
1 Sep 2008	1 Oct 2008	8 Dec 2008
22 Aug 2009	20 Sep 2009	27 Nov 2009
11 Aug 2010	10 Sep 2010	16 Nov 2010
1 Aug 2011	30 Aug 2011	6 Nov 2011
20 Jul 2012	19 Aug 2012	26 Oct 2012
9 Jul 2013	8 Aug 2013	15 Oct 2013

Table S5 - Starting day of the "Christian Calendar", starting day of the weeks that included December 25th – Christmas (always on week 26), the last week of each centered year and the discarded exception weeks after centering.

1	*26*	*week 52*	*exception week*
-		6/20/2004	-
6/27/2004	12/19/2004	6/19/2005	6/26/2005
7/26/2005	12/25/2005	6/25/2006	
7/2/2006	12/24/2006	6/24/2007	-
7/1/2007	12/23/2007	6/22/2008	-
6/29/2008	12/21/2008	6/21/2009	-
6/28/2009	12/20/2009	6/20/2010	-
6/27/2010	12/19/2010	6/19/2011	26 June 2011
7/3/2011	12/25/2011	6/24/2012	-
7/1/2012	12/23/2012	6/23/2013	-
6/30/2013	12/22/2013	-	-

Table S6. Weeks that included Eid-al-Fitr and the discarded exception weeks after centering.

1	*25*	*week 50*	*exception week*
-		5/23/2004	-
5/30/2004	11/14/2004	5/8/2005	-
5/15/2005	10/30/2005	4/23/2006	4/30/2006
5/7/2006	10/22/2006	4/15/2007	-
4/22/2007	10/7/2007	3/30/2008	4/6/2008
4/13/2008	9/28/2008	3/22/2009	3/29/2009
4/5/2009	9/20/2009	3/14/2010	-
3/21/2010	9/5/2010	2/27/2011	3/6/2011
3/13/2011	8/28/2011	2/19/2012	2/26/2012
3/4/2012	8/19/2012	2/10/2013	-
2/17/2013	8/4/2013	1/26/2014	-
2/2/2014	-	-	-

Table S7. Z-scores on the corresponding centered week for all countries in the dataset, calculated from the each country's average for each week, as detailed in the Methods. When z>1 for both the Christmas and Eid-al-Fitr centered calendars, classification was based on the higher score (bold).

	Country	Country Set	Hemisphere	Christmas	Eid-al-Fitr	June Solstice	Dec Solstice
AE	United Arab Emirates	Muslim	North	1.877	**3.023**	0.179	1.313
AF	Afghanistan	Muslim	North	0.654	0.443	0.587	0.889
AL	Albania	Muslim	North	0.372	1.417	0.399	0.491
AR	Argentina	Christian	South	2.190	-2.066	0.395	1.146
AT	Austria	Christian	North	3.598	-0.089	-0.724	1.879
AU	Australia	Christian	South	3.598	-0.089	-0.724	1.879
AW	Aruba	Christian	South	**1.970**	1.960	-0.570	1.502
BA	Bosnia and Herzegovina	Christian	North	-0.312	0.883	0.658	-0.477
BD	Bangladesh	Muslim	North	1.544	**2.576**	0.701	1.062
BE	Belgium	Christian	North	1.713	0.315	0.770	0.350
BG	Bulgaria	Christian	North	0.843	0.476	1.169	-0.443
BH	Bahrain	Muslim	North	1.151	2.492	1.128	1.879
BN	Brunei	Muslim	North	1.183	2.075	0.912	2.005
BO	Bolivia	Christian	South	3.028	0.831	0.074	1.159
BR	Brazil	Christian	South	3.658	-0.580	0.231	1.921
BS	Bahamas	Christian	North	0.185	-0.069	0.298	0.069
BY	Belarus	Christian	North	0.403	0.217	0.106	-0.534
CA	Canada	Christian	North	2.397	0.327	1.159	0.868
CH	Switzerland	Christian	North	4.012	-0.374	0.553	0.984
CL	Chile	Christian	South	1.966	-2.006	-0.634	1.232
CM	Cameroon	Christian	North	1.410	0.926	1.021	0.650
CN	China	Other	North	-0.650	-0.349	0.300	-1.083
CO	Colombia	Christian	North	2.641	-1.164	0.596	1.995
CR	Costa Rica	Christian	North	3.671	-0.728	-0.038	2.110
CY	Cyprus	Christian	North	2.274	-0.376	0.057	0.390
CZ	Czech Republic	Other	North	2.718	-0.166	0.952	1.020
DE	Germany	Christian	North	3.800	0.043	0.759	0.974
DJ	Djibouti	Muslim	North	-0.506	1.507	0.692	-0.071
DK	Denmark	Christian	North	2.842	-0.558	0.602	0.844
DO	Dominican Republic	Christian	North	2.379	-0.861	0.649	1.240
DZ	Algeria	Muslim	North	0.503	0.872	1.611	0.153
EC	Ecuador	Christian	South	3.203	-0.521	0.513	2.062
EE	Estonia	Other	North	1.302	-0.344	1.598	0.541
EG	Egypt	Muslim	North	1.056	**2.278**	-0.302	0.841
ES	Spain	Christian	North	1.587	-0.063	0.391	0.056
ET	Ethiopia	Christian	North	-0.967	-0.585	-0.164	0.013
FI	Finland	Christian	North	2.260	-0.858	1.690	0.854

	Country	Country Set	Hemisphere	Christmas	Eid-al-Fitr	June Solstice	Dec Solstice
FJ	Fiji	Christian	South	3.087	-0.002	-0.437	1.683
FR	France	Christian	North	2.239	-0.050	0.600	1.242
GE	Georgia	Christian	North	-0.033	0.158	0.674	-0.964
GH	Ghana	Christian	North	3.869	-0.417	0.389	1.850
GP	Guadalupe	Christian	North	1.550	-0.059	1.814	1.751
GR	Greece	Christian	North	1.241	-0.158	-0.156	0.056
GT	Guatemala	Christian	North	3.170	-1.062	0.561	2.496
GU	Guam	Christian	South	0.028	1.379	1.080	-0.229
HN	Honduras	Christian	North	2.903	-0.713	0.279	2.062
HR	Croatia	Christian	North	0.953	0.236	1.712	-0.234
HU	Hungary	Christian	North	1.244	0.588	0.928	0.114
ID	Indonesia	Muslim	South	2.792	**3.584**	-0.415	1.337
IE	Ireland	Christian	North	3.498	0.072	0.477	1.052
IL	Israel	Other	North	-1.235	0.085	1.261	-1.446
IN	India	Other	North	1.850	1.315	-0.363	0.756
IQ	Iraq	Muslim	North	-0.833	0.514	-0.704	-0.066
IR	Iran	Muslim	North	-0.597	0.497	0.714	-1.260
IS	Iceland	Christian	North	1.913	-0.698	0.824	1.064
IT	Italy	Christian	North	1.811	0.107	0.056	0.266
JM	Jamaica	Christian	North	1.255	-0.357	1.799	1.190
JO	Jordan	Muslim	North	-0.169	2.317	1.463	-0.334
JP	Japan	Other	North	1.067	0.257	0.468	-0.734
KE	Kenya	Christian	North	**4.217**	1.686	-0.604	3.297
KH	Cambodia	Other	North	1.064	0.988	-0.475	-0.242
KR	South Korea	Other	North	0.994	-1.400	1.172	-0.305
KW	Kuwait	Muslim	North	1.730	**2.384**	0.145	1.855
KZ	Kazakhstan	Muslim	North	0.151	-0.458	1.537	-0.248
LA	Laos	Other	North	1.559	0.670	0.273	0.290
LB	Lebanon	Muslim	North	1.389	**2.497**	0.843	0.205
LK	Sri Lanka	Other	North	2.505	0.443	-0.390	0.970
LT	Lithuania	Christian	North	0.942	0.594	1.249	-0.277
LU	Luxemburg	Christian	North	4.643	-0.968	0.611	1.418
LV	Latvia	Christian	North	1.087	-0.082	2.154	-0.139
MA	Morocco	Muslim	North	0.148	0.484	1.173	-0.669
MD	Moldova	Christian	North	0.648	-0.115	0.626	-0.154
ME	Montenegro	Christian	North	0.004	-0.514	0.145	0.773
MK	Macedonia	Christian	North	-0.789	-0.233	0.786	-0.920
MM	Myanmar	Other	North	1.753	1.324	-1.771	1.998
MN	Mongolia	Other	North	0.087	-0.694	0.785	-0.143
MT	Malta	Christian	North	1.547	-0.059	1.718	1.145
MU	Mauritius	Other	South	2.627	-0.528	-0.212	1.745
MV	Maldives	Muslim	North	-0.475	0.704	-0.215	0.133

	Country	Country Set	Hemisphere	Christmas	Eid-al-Fitr	June Solstice	Dec Solstice
MX	Mexico	Christian	North	3.092	-1.378	0.739	1.967
MY	Malaysia	Muslim	North	1.838	**3.709**	0.174	0.602
MZ	Mozambique	Christian	South	2.243	-0.531	-0.048	1.702
NA	Namibia	Christian	South	3.757	-1.345	0.064	2.812
NG	Nigeria	Christian	North	**4.650**	1.208	-0.227	3.060
NI	Nicaragua	Christian	North	1.199	-0.917	-0.321	2.106
NL	Netherlands	Other	North	1.692	0.031	0.891	0.197
NO	Norway	Christian	North	3.694	-1.155	0.932	2.015
NP	Nepal	Other	North	1.095	**1.588**	-0.454	0.281
NZ	New Zealand	Christian	South	3.230	-0.254	-0.495	1.660
OM	Oman	Muslim	North	0.873	1.943	0.611	1.054
PA	Panama	Christian	North	1.955	0.914	0.009	1.456
PE	Peru	Christian	South	2.317	-2.338	-0.130	1.514
PH	Philippines	Christian	North	2.444	0.981	-1.614	1.819
PK	Pakistan	Muslim	North	**2.282**	2.126	-0.124	1.787
PL	Poland	Christian	North	1.414	0.083	1.341	0.215
PR	Puerto Rico	Christian	North	2.606	-1.690	1.211	2.274
PS	Palestine	Muslim	North	1.152	**1.609**	0.458	0.215
PT	Portugal	Christian	North	2.226	-0.074	0.699	0.859
PY	Paraguay	Christian	South	1.952	-2.259	-1.242	1.278
QA	Qatar	Muslim	North	1.783	**2.986**	-1.061	0.835
RO	Romania	Christian	North	1.458	0.401	0.960	-0.073
RS	Serbia	Christian	North	-0.163	0.474	1.130	-0.390
RU	Russia	Christian	North	0.042	-0.455	1.443	-0.371
SA	Saudi Arabia	Muslim	North	0.271	2.698	-0.037	0.330
SD	Sudan	Muslim	North	0.460	1.662	0.602	0.682
SE	Sweden	Christian	North	1.764	-0.609	1.547	0.383
SG	Singapore	Other	North	**2.238**	1.525	1.339	1.140
SI	Slovenia	Christian	North	0.742	-0.170	1.275	0.018
SK	Slovakia	Christian	North	2.172	0.125	0.913	0.123
SV	El Salvador	Christian	North	3.076	0.144	-0.263	1.603
SY	Syria	Muslim	North	0.136	2.361	0.845	0.101
TH	Thailand	Other	North	0.658	-0.094	-0.761	-0.361
TN	Tunisia	Muslim	North	0.083	2.042	0.523	1.618
TR	Turkey	Muslim	North	-1.084	2.988	1.447	-1.123
TT	Trinidad Tobago	Christian	North	3.526	1.158	-0.016	1.704
TW	Taiwan	Other	North	1.458	-0.249	0.185	0.382
TZ	Tanzania	Christian	South	2.475	-0.365	1.200	1.710
UA	Ukraine	Christian	North	0.497	0.158	0.270	-0.051
UG	Uganda	Christian	North	3.703	0.921	-1.054	2.327
UK	United Kingdom	Christian	North	3.982	0.208	-0.086	1.559

	Country	Country Set	Hemisphere	Christmas	Eid-al-Fitr	June Solstice	Dec Solstice
US	United States of America	Christian	North	3.100	-0.306	1.009	1.137
UY	Uruguay	Christian	South	2.140	-0.462	-1.259	0.879
UZ	Uzbekistan	Muslim	North	-0.590	2.098	1.472	-0.960
VE	Venezuela	Christian	North	3.768	-0.982	-0.292	2.287
VN	Vietnam	Other	North	-0.033	1.300	0.436	-0.380
YE	Yemen	Muslim	North	-0.367	1.963	0.325	-0.181
ZA	South Africa	Christian	South	3.815	0.048	-0.108	2.375
ZM	Zambia	Christian	South	1.804	0.915	-0.098	2.308
ZW	Zimbabwe	Christian	South	3.783	-0.146	1.001	2.569

Table S8A. **Correlation between the Z-scores' time series for all countries in the data set**. Calendars were centered around each of the events and the z-cores calculated, as detailed in the Methods. The high correlation between the Z-score variation around Christmas and around the December Solstice is due to the fact that Christmas often falls on the same week or very close to the December Solstice.

	Christmas	*Eid-al-Fitr*	*June Solstice*	*December Solstice*
Christmas	1.00			
Eid-al-Fitr	-0.28	1.00		
June Solstice	-0.29	-0.06	1.00	
December Solstice	0.80	-0.15	-0.36	1.00

Table S8B. Percentage of countries that were originally classified as Christian, Muslim, or as being located in one of the hemispheres (rows) that showed increased sex-searches (z-scores>1) during Christmas, Eid-al-Fitr or the Solstices (columns).

Identified as	**Increased sex-searches around:** Christmas	Eid-al-Fitr	June Sltc	Dec Sltc
Christian	80%	6%	25%	56%
Muslim	40%	77%	23%	30%
Southern Hemisphere	95%	14%	14%	90%
Northern Hemisphere	64%	28%	26%	36%

Table S9. Monthly birth data available for countries from Supplementary Table 2. First column, countries that belong to the ”Other” country set are marked with a blue background, countries that belong to the ”Muslim” country set with a green background, and countries belonging to the ”Christian” country set with a white background. At the bottom of the table are the only four countries from the Southern Hemisphere for which we could find birth data, and all four were classified as Christian. Dark shaded area coincides with the period for which we have GT data and these were the years used in all birth plots.

	2013	2012	2011	2010	2009	2008	2007	2006	2005	2004	2003	2002	2001	2000	1999	1998	1997	1996	1995	1994	1993	1992	1991	1990	1989	1988	1987	1986	1985	1984	1983	1982	1981	1980	1979	1978	1977	1976	1975	1974	1973	1972	1971	1970	1969
Czech Republic																																													
Estonia																																													
Israel																																													
Japan																																													
Singapore																																													
South Korea																																													
Sri Lanka																																													
Albania																																													
Bahrain																																													
Egypt																																													
Kazakhstan																																													
Kuwait																																													
Lebanon																																													
Malaysia																																													
Maldives																																													
Pakistan																																													
Palestine																																													
Qatar																																													
Tunisia																																													
Turkey																																													
Austria																																													
Belgium																																													
Bosnia and Herzegovina																																													
Bulgaria																																													
Canada																																													
Costa Rica																																													
Croatia																																													
Cyprus																																													
Denmark																																													
Finland																																													
France																																													
Germany																																													
Hungary																																													
Iceland																																													
Italy																																													
Latvia																																													
Lithuania																																													
Luxemburg																																													
Macedonia																																													
Malta																																													
Mexico																																													
Moldova																																													
Netherlands																																													
Norway																																													
Panama																																													
Poland																																													
Portugal																																													
Puerto Rico																																													
Romania																																													
Russia																																													
Serbia																																													
Slovakia																																													
Slovenia																																													
Spain																																													
Sweden																																													
Switzerland																																													
Ukraine																																													
USA																																													
Venezuela																																													
Australia																																													
Chile																																													
New Zealand																																													
South Africa																																													

Table S10. Multiple linear regression statistics with all three ANEW dimensions, using weekly ANEW means as independent variables and sex search volume as dependent variable. A) Regression over all years of data. B) Regression over an average year centered on Christmas (USA, Australia, Brazil, Argentina, Chile) and Eid-al-Fitr (Indonesia and Turkey) – Independent variables are: [mean ANEW values averaged across years – the holiday center] (i.e, Christmas is 0,0,0), dependent variable is the number of sex-searches averaged across years of data. R^2 columns indicate the coefficient of determination for the regression, F_p columns indicate the p-value for the F-statistic of the overall model, B columns indicate the coefficients for the independent variables in the regression. t-test p columns indicate the individual t-test p values for the independent variables. Bold values denote significance at $\alpha=0.05$, italicized values denote Bonferroni corrected significance over countries per variable choice $\alpha=0.05/7= 0.00714$.

A

Country	R^2	Valence B	Dominance B	Arousal B	F_p	Valence t-test p	Dominance t-test p	Arousal t-test p
USA	0.399	197.69	-379.75	-0.36	***1.18E-20***	***4.76E-18***	***1.06E-12***	0.972
Australia	0.274	55.77	-92.18	-25.05	***2.91E-12***	***1.10E-07***	***4.22E-06***	***9.79E-06***
Brazil	0.401	12.47	37.78	90.74	***1.19E-15***	0.416	0.423	***4.79E-08***
Argentina	0.388	39.22	-36.67	-8.79	***1.40E-14***	***2.59E-09***	***1.91E-03***	0.0786
Chile	0.240	4.93	26.63	-28.93	***1.68E-10***	0.602	0.280	***6.36E-10***
Indonesia	0.187	72.13	-127.96	-12.95	***1.87E-06***	***1.24E-07***	***5.28E-04***	0.366
Turkey	0.135	6.66	-1.72	16.11	***4.22E-04***	0.128	0.893	***1.83E-04***

B

Country	R^2	Valence B	Dominance B	Arousal B	F_p	Valence t-test p	Dominance t-test p	Arousal t-test p
USA	0.426	193.002	-427.958	96.678	***6.20E-06***	***2.94E-07***	***2.77E-05***	0.0632
Australia	0.566	95.519	-128.290	19.318	***8.40E-09***	***4.67E-08***	***1.44E-03***	0.225
Brazil	0.488	90.086	-148.254	35.561	***4.15E-07***	***3.06E-05***	0.0340	0.116
Argentina	0.530	57.468	-65.493	-2.228	***5.51E-08***	***3.61E-07***	0.0145	0.871
Chile	0.697	70.497	-81.955	12.632	***1.73E-12***	***8.21E-08***	0.0123	0.0606
Indonesia	0.267	144.696	-272.516	-53.604	***2.34E-03***	**8.40E-03**	0.0213	0.271
Turkey	0.260	7.835	-81.301	41.880	***2.94E-03***	0.503	0.0103	**0.0220**

Table S11. Linear regression statistics for individual ANEW dimensions, using weekly ANEW means as independent variables and sex search volume as dependent variable. A: Regression over all years of data. B: Regression over an average year centered on Christmas (USA, Australia, Brazil, Argentina, Chile) and Eid-al-Fitr (Indonesia and Turkey) – Independent variables are: [mean ANEW value averaged across years – the holiday center] (i.e, Christmas is 0), dependent variable is the number of sex-searches averaged across years of data. Independent variables from top to bottom: Valence, Dominance, and Arousal. R^2 columns indicate the coefficient of determination for the regression, F_p columns indicate the p-value for the F-statistic of the overall model, B columns indicate the coefficients for the independent variables in the regression. Bold values denote significance at α=0.05, italicized values denote Bonferroni corrected significance over countries per variable choice α=0.05/7 = 0.00714.

A

Country	**Valence R^2**	**Valence F_p**	**Valence B**
USA	0.057	***8.99E-04***	54.80
Australia	0.065	***5.34E-04***	-10.74
Brazil	0.004	0.434	6.57
Argentina	0.255	***1.78E-10***	13.25
Chile	0.019	0.0680	8.52
Indonesia	0.091	***2.29E-04***	27.35
Turkey	0.008	0.3.07	3.16

Country	**Dominance R^2**	**Dominance F_p**	**Dominance B**
USA	0.052	***1.46E-03***	-101.86
Australia	0.120	***1.84E-06***	-30.11
Brazil	0.141	***3.21E-06***	115.42
Argentina	0.143	***3.67E-06***	15.86
Chile	0.001	0.711	4.19
Indonesia	0.007	0.302	21.45
Turkey	0.031	**4.84E-02**	18.09

Country	**Arousal R^2**	**Arousal F_p**	**Arousal B**
USA	0.102	***6.20E-06***	-41.47
Australia	0.148	***9.78E-08***	-15.07
Brazil	0.347	***6.28E-15***	90.83
Argentina	0.026	0.0573	6.66
Chile	0.186	***1.48E-09***	-23.98
Indonesia	0.007	0.323	11.16
Turkey	0.105	***1.95E-04***	13.97

B

Country	Valence R^2	Valence F_p	Valence B
USA	0.166	***2.76E-03***	80.924
Australia	0.459	***3.39E-08***	56.364
Brazil	0.437	***9.41E-08***	51.052
Argentina	0.418	***2.25E-07***	31.673
Chile	0.652	***4.82E-13***	43.522
Indonesia	0.008	0.541	19.959
Turkey	0.043	0.150	8.871

Country	Dominance R^2	Dominance F_p	Dominance B
USA	0.002	0.778	-20.481
Australia	0.167	***2.66E-03***	74.668
Brazil	0.214	***5.48E-04***	121.891
Argentina	0.138	**6.68E-03**	39.978
Chile	0.426	***1.55E-07***	97.791
Indonesia	0.049	0.123	-94.162
Turkey	0.005	0.612	-11.387

Country	Arousal R^2	Arousal F_p	Arousal B
USA	0.000	0.945	-3.900
Australia	0.165	***2.85E-03***	42.919
Brazil	0.010	0.490	-14.894
Argentina	0.006	0.598	7.270
Chile	0.000	0.948	-0.640
Indonesia	0.146	***6.16E-03***	-112.497
Turkey	0.125	0.0119	28.478

Table S12 – Ordinary least squares linear regression statistics for sex-searches v.s proximity in eigenmood to Christmas. The components selected were the two components (eigenbins) that most distinguish the holiday week from other weeks (see Methods S11). In the Components column, v stands for valence, d for dominance, and a for arousal. R^2 is the coefficient of determination, F_p is the p-value of the overall F-test for the regression, and the Slope is the slope of regressions. ρ is the Pearson's correlation coefficient between proximity and sex searches, ρ_D is the Brownian distance correlation coefficient, and $DCov_p$ is the p-value for the Brownian distance covariance calculated from a permutation test of the data. Bold denotes significance at α=0.05, italicized values denote Bonferroni corrected significance over countries per variable choice α=0.05/7 = 0.00714, underlined denote Bonferroni corrected significance over all table possibilities α=0.05/21 = 0.00238.

Christmas

Country	Components	R²	Fp	Slope	ρ	ρD	DCovp
USA	v4, v5	0.38	***5.08E-06***	6.50E+04	0.616	0.559	0.001
Australia	d5, d8	0.392	***2.52E-06***	2.44E+04	0.626	0.576	0.001
Brazil	a3, v2	0.504	***3.35E-08***	9.47E+03	0.71	0.624	0.001
Argentina	v5, d3	0.577	***6.11E-10***	5.35E+03	0.759	0.712	0.001
Chile	v3, d8	0.419	***1.16E-06***	7.96E+03	0.647	0.646	0.001
Indonesia	a3, v3	0.448	***2.66E-07***	9.95E+03	0.67	0.657	0.001
Turkey	a3, d3	0.373	***6.46E-06***	-1.42E+03	-0.611	0.618	0.001

Eid-al-Fitr without Ramadan

Country	Components	R²	Fp	Slope	ρ	ρD	DCovp
USA	a6, v3	0.065	0.107	1.57E+05	0.256	0.328	0.118
Australia	v3, v4	0.02	0.381	-1.62E+03	-0.141	0.317	0.154
Brazil	a3, d8	0.147	**0.0147**	-4.07E+04	-0.383	0.539	0.001
Argentina	v9, d3	0.598	***3.08E-09***	-2.32E+04	-0.773	0.735	0.001
Chile	a6, d2	0.189	***5.00E-03***	-1.15E+04	-0.435	0.461	0.005
Indonesia	v3, d3	0.637	***6.87E-10***	8.70E+03	0.798	0.712	0.001
Turkey	a3, d3	0.737	***6.94E-13***	4.81E+02	0.859	0.858	0.001

Eid-al-Fitr

Country	Components	R²	Fp	Slope	ρ	ρD	DCovp
USA	a6, v3	0.077	0.0645	1.75E+05	0.278	0.343	0.061
Australia	v3, v4	0.038	0.198	-2.30E+03	-0.196	0.333	0.085
Brazil	a3, d8	0.124	**0.0204**	-3.54E+04	-0.353	0.516	0.001
Argentina	v9, d3	0.593	***6.23E-10***	-2.31E+04	-0.77	0.73	0.001
Chile	a6, d2	0.191	***3.03E-03***	-1.04E+04	-0.437	0.489	0.001
Indonesia	v3, d3	0.407	***3.19E-06***	9.85E+03	0.638	0.621	0.001
Turkey	a3, d3	0.339	***3.42E-05***	3.32E+02	0.582	0.634	0.001

Table S13– List of words and expressions removed from the Twitter/ANEW analysis.

"merry christmas"
"merry xmas"
"happy christmas"
"happy xmas"
"happy new year"
"happy newyear"
"happy thanksgiving"
"happy ramadan"
"happy easter"
"happy holidays"
"happy hanukkah"
"happy hanukah"
"happy ramadan"
"happy eid"
"happy halloween"
"happy valentines day"
"happy valentine's day"
"feliz natal"
"feliz ano"
"feliz pascoa"
"pascoa feliz"
"feliz thanksgiving"
"feliz navidad"
"feliz ano nuevo"
"feliz ano novo"
"feliz ramadan"
"feliz año"
"feliz páscoa"
"páscoa feliz "
"feliz año nuevo"
"happy anzac day"
"feliz anzac day"
"happy adelaide cup"
"feliz adelaide cup"
"happy all saints day"
"feliz all saints day"
"happy all souls day"
"feliz all souls day"
"happy andalucia day"
"feliz andalucia day"
"happy arafat day"
"feliz arafat day"
"happy armistice day"
"feliz armistice day"
"happy army day"
"feliz army day"
"happy asahna bucha day"
"feliz asahna bucha day"
"happy ascension day"
"feliz ascension day"
"happy ash monday"
"feliz ash monday"
"happy ash wednesday"
"feliz ash wednesday"
"happy ashura"
"feliz ashura"
"happy assumption day"
"feliz assumption day"
"happy asturias"
"feliz asturias"
"happy auckland province"
"feliz auckland province"
"happy august bank holiday"
"feliz august bank holiday"
"happy august holiday"
"feliz august holiday"
"happy australia day"
"feliz australia day"
"happy australia day holiday"
"feliz australia day holiday"
"happy autumnal equinox day"
"feliz autumnal equinox day"
"happy awal muharram"
"feliz awal muharram"
"happy balearic islands"
"feliz balearic islands"
"happy bank holiday"
"feliz bank holiday"
"happy bastille day"
"feliz bastille day"
"happy battle of the boyne"
"feliz battle of the boyne"
"happy benito juarezs birthday"
"feliz benito juarezs birthday"
"happy berchtolds day"
"feliz berchtolds day"
"happy bettagsmontag"
"feliz bettagsmontag"
"happy bhogi"
"feliz bhogi"
"happy bicentennial of the constituent assembly of 1813"
"feliz bicentennial of the constituent assembly of 1813"
"happy birthday of muhammad iqbal"
"feliz birthday of muhammad iqbal"
"happy birthday of prophet muhammad"
"feliz birthday of prophet muhammad"
"happy birthday of quaid-e-azam muhammad ali jinnah"
"feliz birthday of quaid-e-azam muhammad ali jinnah"
"happy birthday of spb yang di pertuan agong"
"feliz birthday of spb yang di pertuan agong"
"happy birthday of the sultan of selangor"
"feliz birthday of the sultan of selangor"

"happy boxing day"
"feliz boxing day"
"happy bridge public"
"feliz bridge public"
"happy buddha purnima"
"feliz buddha purnima"
"happy buddhas birthday"
"feliz buddhas birthday"
"happy canada day"
"feliz canada day"
"happy canary islands"
"feliz canary islands"
"happy canberra day"
"feliz canberra day"
"happy canterbury"
"feliz canterbury"
"happy carnival"
"feliz carnival"
"happy castile-la mancha"
"feliz castile-la mancha"
"happy catalonia"
"feliz catalonia"
"happy celebration of the golden spurs"
"feliz celebration of the golden spurs"
"happy ceuta"
"feliz ceuta"
"happy chanukah"
"feliz chanukah"
"happy chatham islands"
"feliz chatham islands"
"happy childrens day"
"feliz childrens day"
"happy chinese new year"
"feliz chinese new year"
"happy chinese new year eve"
"feliz chinese new year eve"
"happy ching ming"
"feliz ching ming"
"happy christmas day"
"feliz christmas day"
"happy christmas eve"
"feliz christmas eve"
"happy christmas eve day"
"feliz christmas eve day"
"happy christmas"
"feliz christmas"
"happy chulalongkorn day"
"feliz chulalongkorn day"
"happy chung yeung festival"
"feliz chung yeung festival"
"happy cinco de mayo"
"feliz cinco de mayo"
"happy civic day"
"feliz civic day"
"happy columbus day"
"feliz columbus day"
"happy coming of age day"
"feliz coming of age day"
"happy community day"
"feliz community day"
"happy community festival of madrid"
"feliz community festival of madrid"
"happy constitution day"
"feliz constitution day"
"happy constitution memorial day"
"feliz constitution memorial day"
"happy corpus christi"
"feliz corpus christi"
"happy culture day"
"feliz culture day"
"happy day after christmas"
"feliz day after christmas"
"happy day after new years day"
"feliz day after new years day"
"happy day of atonement"
"feliz day of atonement"
"happy day of good will"
"feliz day of good will"
"happy day of national sovereignty"
"feliz day of national sovereignty"
"happy day of reconciliation"
"feliz day of reconciliation"
"happy day of reformation"
"feliz day of reformation"
"happy day of unity"
"feliz day of unity"
"happy day of respect for cultural diversity"
"feliz day of respect for cultural diversity"
"happy day of the battle of salta"
"feliz day of the battle of salta"
"happy day of the constitution of the slovak republic"
"feliz day of the constitution of the slovak republic"
"happy day of the dead"
"feliz day of the dead"
"happy day of the establishment of the slovak republic"
"feliz day of the establishment of the slovak republic"
"happy day of the german-speaking community of belgium"
"feliz day of the german-speaking community of belgium"
"happy day of the virgin of guadalupe"
"feliz day of the virgin of guadalupe"
"happy day of victory over fascism"
"feliz day of victory over fascism"

"happy declaration of independence"
"feliz declaration of independence"
"happy deepavali"
"feliz deepavali"
"happy deewali"
"feliz deewali"
"happy defence of the motherland"
"feliz defence of the motherland"
"happy discovery day"
"feliz discovery day"
"happy double ninth day"
"feliz double ninth day"
"happy dragon boat festival"
"feliz dragon boat festival"
"happy dussehra"
"feliz dussehra"
"happy early may bank holiday"
"feliz early may bank holiday"
"happy easter"
"feliz easter"
"happy easter monday"
"feliz easter monday"
"happy easter sunday"
"feliz easter sunday"
"happy eid al adha"
"feliz eid al adha"
"happy eid al fitr"
"feliz eid al fitr"
"happy eid milad un-nabi"
"feliz eid milad un-nabi"
"happy eid ul-azha day 1"
"feliz eid ul-azha day 1"
"happy eid ul-azha day 2"
"feliz eid ul-azha day 2"
"happy eid-ul-fitr"
"feliz eid-ul-fitr"
"happy emancipation day"
"feliz emancipation day"
"happy epiphany"
"feliz epiphany"
"happy extremadura"
"feliz extremadura"
"happy family & community day"
"feliz family & community day"
"happy family day"
"feliz family day"
"happy fathers day"
"feliz fathers day"
"happy feast of st ambrose"
"feliz feast of st ambrose"
"happy feast of st anthony"
"feliz feast of st anthony"
"happy feast of st john the baptist"
"feliz feast of st john the baptist"
"happy federal territory day"
"feliz federal territory day"
"happy fiesta de san isidro"
"feliz fiesta de san isidro"
"happy foundation day"
"feliz foundation day"
"happy foundation of the independent czechoslovak state"
"feliz foundation of the independent czechoslovak state"
"happy freedom day"
"feliz freedom day"
"happy french community"
"feliz french community"
"happy ganesh chaturthi"
"feliz ganesh chaturthi"
"happy general prayer day"
"feliz general prayer day"
"happy german unity day"
"feliz german unity day"
"happy good friday"
"feliz good friday"
"happy greenery day"
"feliz greenery day"
"happy groundhog day"
"feliz groundhog day"
"happy guru nanak birthday"
"feliz guru nanak birthday"
"happy guy fawkes night"
"feliz guy fawkes night"
"happy h.m. kings birthday"
"feliz h.m. kings birthday"
"happy h.m. queens birthday"
"feliz h.m. queens birthday"
"happy hangeul day"
"feliz hangeul day"
"happy hari hol almarhum sultan iskandar"
"feliz hari hol almarhum sultan iskandar"
"happy hari raya haji"
"feliz hari raya haji"
"happy hari raya nyepi"
"feliz hari raya nyepi"
"happy hari raya puasa"
"feliz hari raya puasa"
"happy harvest festival"
"feliz harvest festival"
"happy hawkes bay"
"feliz hawkes bay"
"happy health-sports day"
"feliz health-sports day"
"happy heritage day"
"feliz heritage day"
"happy hijri new years day"
"feliz hijri new years day"

"happy hispanic day"
"feliz hispanic day"
"happy holi"
"feliz holi"
"happy holy spirit monday"
"feliz holy spirit monday"
"happy human rights day"
"feliz human rights day"
"happy idul adha"
"feliz idul adha"
"happy idul fitr"
"feliz idul fitr"
"happy idul juha"
"feliz idul juha"
"happy immaculate conception day"
"feliz immaculate conception day"
"happy independence day"
"feliz independence day"
"happy independence day of chile"
"feliz independence day of chile"
"happy independence day"
"feliz independence day"
"happy independence of cartagena"
"feliz independence of cartagena"
"happy isra miraj"
"feliz isra miraj"
"happy israa & miaraj night"
"feliz israa & miaraj night"
"happy jan hus day"
"feliz jan hus day"
"happy janmashtami"
"feliz janmashtami"
"happy june holiday"
"feliz june holiday"
"happy kannada rajyothsava"
"feliz kannada rajyothsava"
"happy kashmir day"
"feliz kashmir day"
"happy kings feast"
"feliz kings feast"
"happy knabenschiessen"
"feliz knabenschiessen"
"happy korean new year"
"feliz korean new year"
"happy la rioja"
"feliz la rioja"
"happy labor day"
"feliz labor day"
"happy labour day"
"feliz labour day"
"happy labour thanksgiving day"
"feliz labour thanksgiving day"
"happy labour day"
"feliz labour day"
"happy lady of aparecida"
"feliz lady of aparecida"
"happy lantern festival"
"feliz lantern festival"
"happy late mid autumn festival"
"feliz late mid autumn festival"
"happy liberation day"
"feliz liberation day"
"happy liberation day czech republic"
"feliz liberation day czech republic"
"happy maha shivratri"
"feliz maha shivratri"
"happy maharashtra day"
"feliz maharashtra day"
"happy mahatma gandhi birthday"
"feliz mahatma gandhi birthday"
"happy mahavir jayanti"
"feliz mahavir jayanti"
"happy makha bucha day"
"feliz makha bucha day"
"happy malaysia day"
"feliz malaysia day"
"happy malvinas day"
"feliz malvinas day"
"happy march 1st movement"
"feliz march 1st movement"
"happy marine day"
"feliz marine day"
"happy marlborough"
"feliz marlborough"
"happy martin luther king day"
"feliz martin luther king day"
"happy maulidur rasul"
"feliz maulidur rasul"
"happy maundy thursday"
"feliz maundy thursday"
"happy may bank holiday"
"feliz may bank holiday"
"happy may day"
"feliz may day"
"happy may day revolution"
"feliz may day revolution"
"happy melbourne cup day"
"feliz melbourne cup day"
"happy memorial day"
"feliz memorial day"
"happy mid autumn festival"
"feliz mid autumn festival"
"happy midsummer day"
"feliz midsummer day"
"happy milad-un-nabi"
"feliz milad-un-nabi"
"happy mothering sunday"
"feliz mothering sunday"

"happy mothers day"
"feliz mothers day"
"happy muharram"
"feliz muharram"
"happy murcia"
"feliz murcia"
"happy national day"
"feliz national day"
"happy national flag day"
"feliz national flag day"
"happy national foundation day"
"feliz national foundation day"
"happy national remembrance day"
"feliz national remembrance day"
"happy national sovereignty and children's day"
"feliz national sovereignty and children's day"
"happy national womens day"
"feliz national womens day"
"happy national holiday"
"feliz national holiday"
"happy navy day"
"feliz navy day"
"happy nelson"
"feliz nelson"
"happy new year"
"feliz new year"
"happy new years day"
"feliz new years day"
"happy new years eve"
"feliz new years eve"
"happy new years"
"feliz new years"
"happy orthodox christmas day"
"feliz orthodox christmas day"
"happy orthodox easter monday"
"feliz orthodox easter monday"
"happy orthodox good friday"
"feliz orthodox good friday"
"happy otago province"
"feliz otago province"
"happy our lady of mount carmel"
"feliz our lady of mount carmel"
"happy our lady of the almudena"
"feliz our lady of the almudena"
"happy pakistan day"
"feliz pakistan day"
"happy pancake tuesday"
"feliz pancake tuesday"
"happy parsi new year"
"feliz parsi new year"
"happy passover"
"feliz passover"
"happy peace memorial day"
"feliz peace memorial day"
"happy pentecost"
"feliz pentecost"
"happy picnic day"
"feliz picnic day"
"happy pongal"
"feliz pongal"
"happy portugal day"
"feliz portugal day"
"happy presidential elections"
"feliz presidential elections"
"happy presidents day"
"feliz presidents day"
"happy public holiday"
"feliz public holiday"
"happy purim"
"feliz purim"
"happy queens birthday"
"feliz queens birthday"
"happy race day"
"feliz race day"
"happy ram navami"
"feliz ram navami"
"happy ramazan feast"
"feliz ramazan feast"
"happy reformation day"
"feliz reformation day"
"happy remembrance day"
"feliz remembrance day"
"happy repentance day"
"feliz repentance day"
"happy republic day"
"feliz republic day"
"happy respect for the aged day"
"feliz respect for the aged day"
"happy restoration day"
"feliz restoration day"
"happy restoration day of the independent czech state"
"feliz restoration day of the independent czech state"
"happy restoration of independence"
"feliz restoration of independence"
"happy revolution day"
"feliz revolution day"
"happy sacred heart"
"feliz sacred heart"
"happy sacrifice feast"
"feliz sacrifice feast"
"happy saint leopold"
"feliz saint leopold"
"happy saint nicholas"
"feliz saint nicholas"
"happy saint peter and saint paul"
"feliz saint peter and saint paul"

"happy saint stephens day"
"feliz saint stephens day"
"happy sechselauten"
"feliz sechselauten"
"happy second day of christmas"
"feliz second day of christmas"
"happy showa day"
"feliz showa day"
"happy simchat torah"
"feliz simchat torah"
"happy slovak national uprising anniversary"
"feliz slovak national uprising anniversary"
"happy songkran festival"
"feliz songkran festival"
"happy south canterbury"
"feliz south canterbury"
"happy southland"
"feliz southland"
"happy special administration region (sar) day"
"feliz special administration region (sar) day"
"happy st andrews day"
"feliz st andrews day"
"happy st cyril and methodius day"
"feliz st cyril and methodius day"
"happy st davids day"
"feliz st davids day"
"happy st georges day"
"feliz st georges day"
"happy st james day"
"feliz st james day"
"happy st josephs day"
"feliz st josephs day"
"happy st martins day"
"feliz st martins day"
"happy st patricks day"
"feliz st patricks day"
"happy st stephens day"
"feliz st stephens day"
"happy st wenceslas day"
"feliz st wenceslas day"
"happy struggle for freedom and democracy day"
"feliz struggle for freedom and democracy day"
"happy sukkot"
"feliz sukkot"
"happy swiss federal fast"
"feliz swiss federal fast"
"happy taranaki"
"feliz taranaki"
"happy thaipusam"
"feliz thaipusam"
"happy thanksgiving"
"feliz thanksgiving"
"happy buddhas birthday"
"feliz buddhas birthday"
"happy emperors birthday"
"feliz emperors birthday"
"happy national holiday of quebec"
"feliz national holiday of quebec"
"happy ochi day"
"feliz ochi day"
"happy patron saint of turin"
"feliz patron saint of turin"
"happy thiruvalluvar day"
"feliz thiruvalluvar day"
"happy tiradentes day"
"feliz tiradentes day"
"happy tomb sweeping festival"
"feliz tomb sweeping festival"
"happy tomb sweeping holiday"
"feliz tomb sweeping holiday"
"happy truth and justice memorial day"
"feliz truth and justice memorial day"
"happy uae national day"
"feliz uae national day"
"happy ugadi"
"feliz ugadi"
"happy urs mubarak of hazrat data gunj bakhsh"
"feliz urs mubarak of hazrat data gunj bakhsh"
"happy v-e day"
"feliz v-e day"
"happy valencia"
"feliz valencia"
"happy vernal equinox day"
"feliz vernal equinox day"
"happy vesak day"
"feliz vesak day"
"happy veterans day"
"feliz veterans day"
"happy victoria day"
"feliz victoria day"
"happy victory day"
"feliz victory day"
"happy visakha bucha day"
"feliz visakha bucha day"
"happy waisak day"
"feliz waisak day"
"happy waitangi day"
"feliz waitangi day"
"happy wellington province"
"feliz wellington province"
"happy wesak day"
"feliz wesak day"
"happy westland"
"feliz westland"
"happy whitmonday"
"feliz whitmonday"
"happy womens day"

“feliz womens day”
“happy youth day”
“feliz youth day”
“happy zumbi dos palmares”
“feliz zumbi dos palmares”
“christmas”
“navidad”
“natal”
“valentine”
“san valentín”
“valentín”
“san valentin”
“valentin”
“valentim
